# Supplementary material for: Malfunction of airway basal stem cells plays a crucial role in pathophysiology of tracheobronchopathia osteoplastica
Source: Nat Commun. 2022 Mar 14;13:1309. doi: 10.1038/s41467-022-28903-7 (PMC8921516; doi:10.1038/s41467-022-28903-7)
Supplement: Supplementary file 1 — Supplementary information [file 41467_2022_28903_MOESM1_ESM.pdf]

# SUPPLEMENTARY INFORMATION

## Malfunction of Airway Basal Stem Cells plays a crucial role in Pathophysiology of Tracheobronchopathia Osteoplastica

Yue Hong*, Shan Shan, Ye Gu, Haidong Huang, Quncheng Zhang, Yang Han, Yongpin Dong, Zeyu Liu, Moli Huang, Tao Ren*

* Corresponding authors

**Content including:**

SUPPLEMENTARY FIGURES AND LEGENDS

SUPPLEMENTARY TABLES

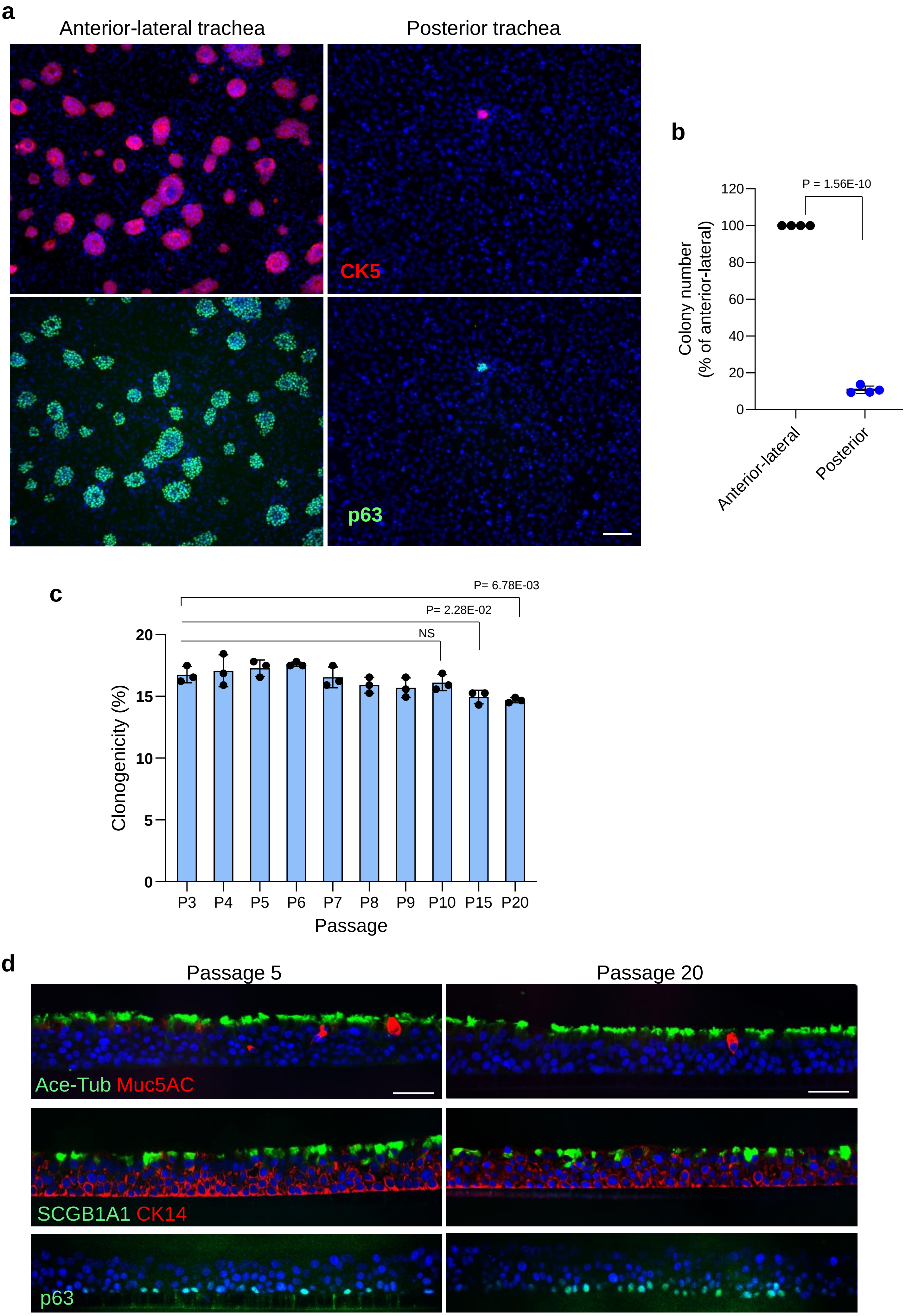
a
Anterior-lateral trachea
Posterior trachea
CK5
p63
b
P = 1.56E-10
Colony number
(% of anterior-lateral)
120
100
80
60
40
20
0
Anterior-lateral
Posterior
c
P= 6.78E-03
P= 2.28E-02
NS
Clonogenicity (%)
20
15
10
5
0
P3
P4
P5
P6
P7
P8
P9
P10
P15
P20
Passage
d
Passage 5
Passage 20
Ace-Tub Muc5AC
SCGB1A1 CK14
p63

**Supplementary Figure 1. Spatial distribution of proliferative basal stem cells in human tracheas, and stability of cell maintenance in vitro. (a)** Representative images of primary cultures of tracheal stem cells derived from anterior-lateral and posterior portions of normal donors, respectively. Cell character was confirmed by immunofluorescent staining with basal cell markers, CK5 and p63. Scale bar, 200μm. **(b)** Scatter plot depicting clone numbers counted from sub-regional cultures of 4 independent biological samples, as percentage values of anterior-lateral counterparts. Bars represent mean ± SD, and p value is indicated to show statistical significance. **(c)** Self-renewal ability of isolated normal basal stem cells was assessed by clonogenic assay, shown as percentage of plated cells that formed typical clones on Day 4-5 post-seeding from passage 3 to 20 (n=3 experimental repeats/passage). Results are represented as mean ± SD, and p values are indicated to show statistical significance ($^{NS}$ $p > 0.05$). **(d)** Representative images showing differentiation ability of passaged cells examined by immunofluorescent staining of ALI sections (n=3 experimental repeats) with functional cell markers, acetylated tubulin (ciliated), Muc5AC (goblet), SCGB1A1 (club) and CK14, p63 (basal). Scale bar, 40μm. (b, c) two-tailed Student's t-tests. Source data are provided as a Source Data file.

**a** P4 clones

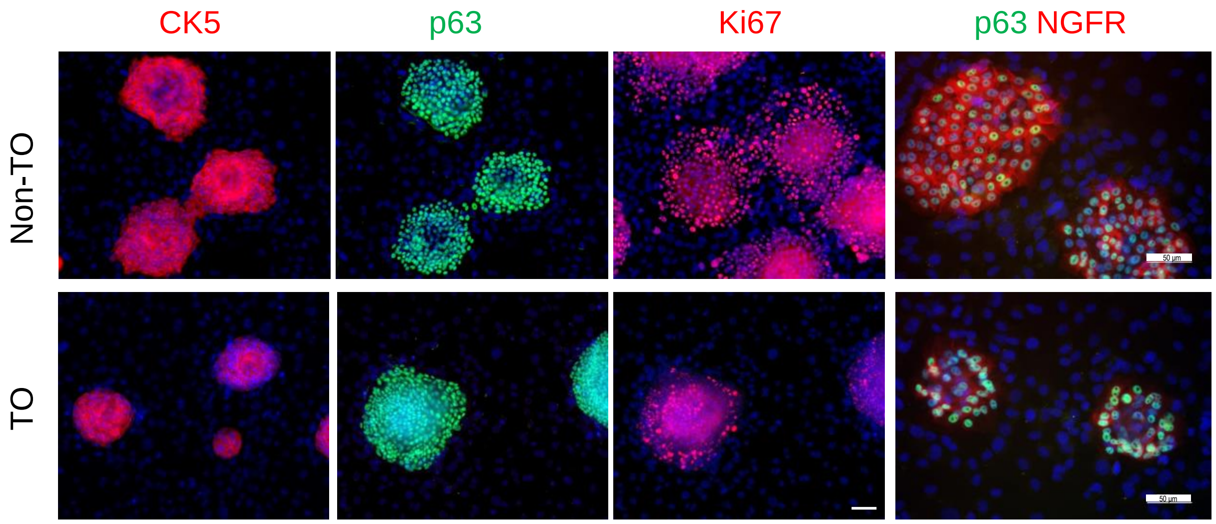


**b** P7 clones

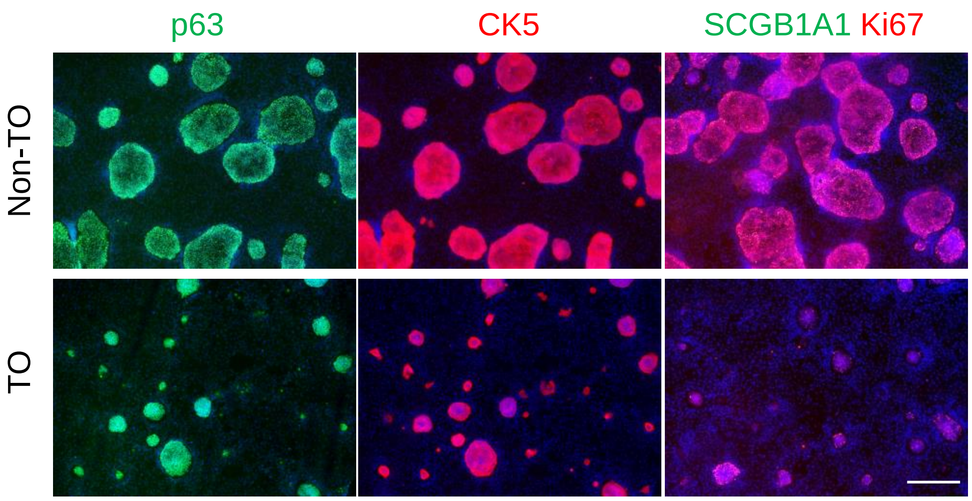


**c**

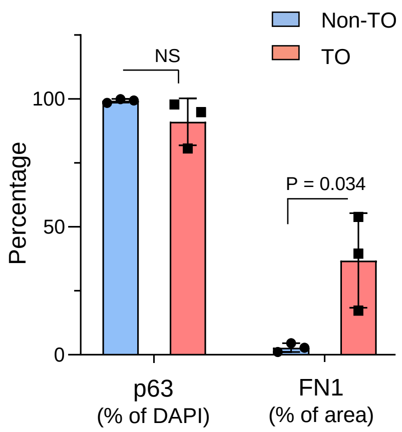


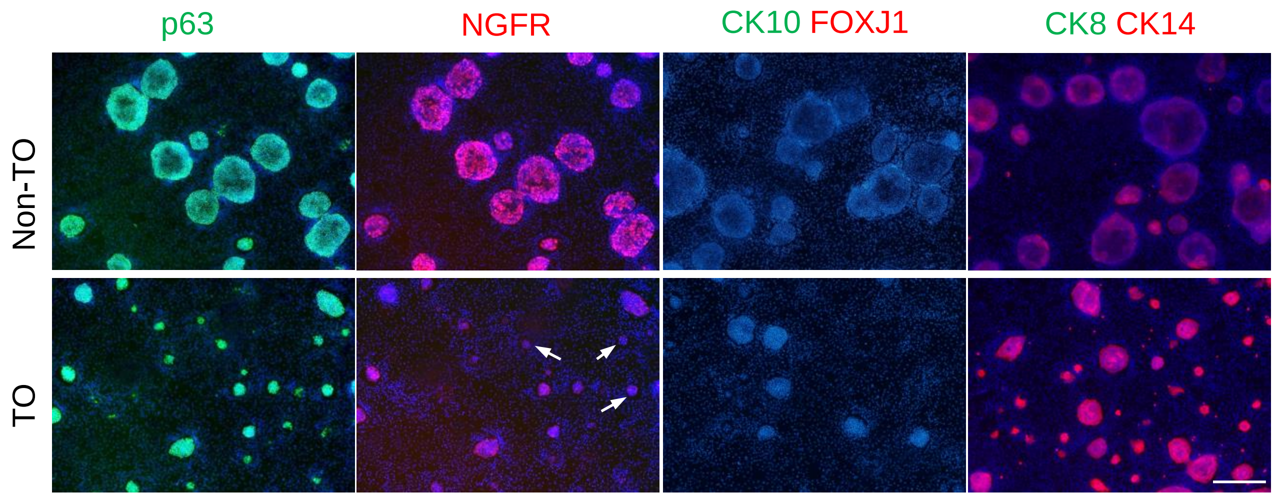

**Supplementary Figure 2. Characterization of isolated TBBCs derived from non-TO controls and TO patients. (a)** Representative immunofluorescent images showing the expression of key basal cell markers CK5, p63, NGFR and Ki67 on clones from P4 culture. Scale bar, 50µm. **(b)** Representative immunofluorescent images of clones from P7 culture stained with a panel of antibodies against basal cell markers (p63, CK5, NGFR and CK14), proliferation indicator (Ki67), differentiating marker (CK8) and cell-type specific markers (SCGB1A1, CK10, FOXJ1). Scale bar, 500µm. Cells with strong p63 immunoreactivity but almost no NGFR labeling are highlighted by white arrows as examples. **(c)** Quantification of immunofluorescence results from p63 and FN1 labelled TBBC monolayers using Fiji. For p63, results are presented as mean of percentage of positive cells in total DAPI nuclei via particle analysis; whilst for FN1, due to its diffuse pattern and deposition in extracellular space, results are presented as mean of area fraction. Error bars of ±SD and p-values are indicated in the graph ($^{NS}$ $p > 0.05$). (a, b, c) n=3 biologically independent experiments. (c) two-tailed Student's t-tests. Source data are provided as a Source Data file.

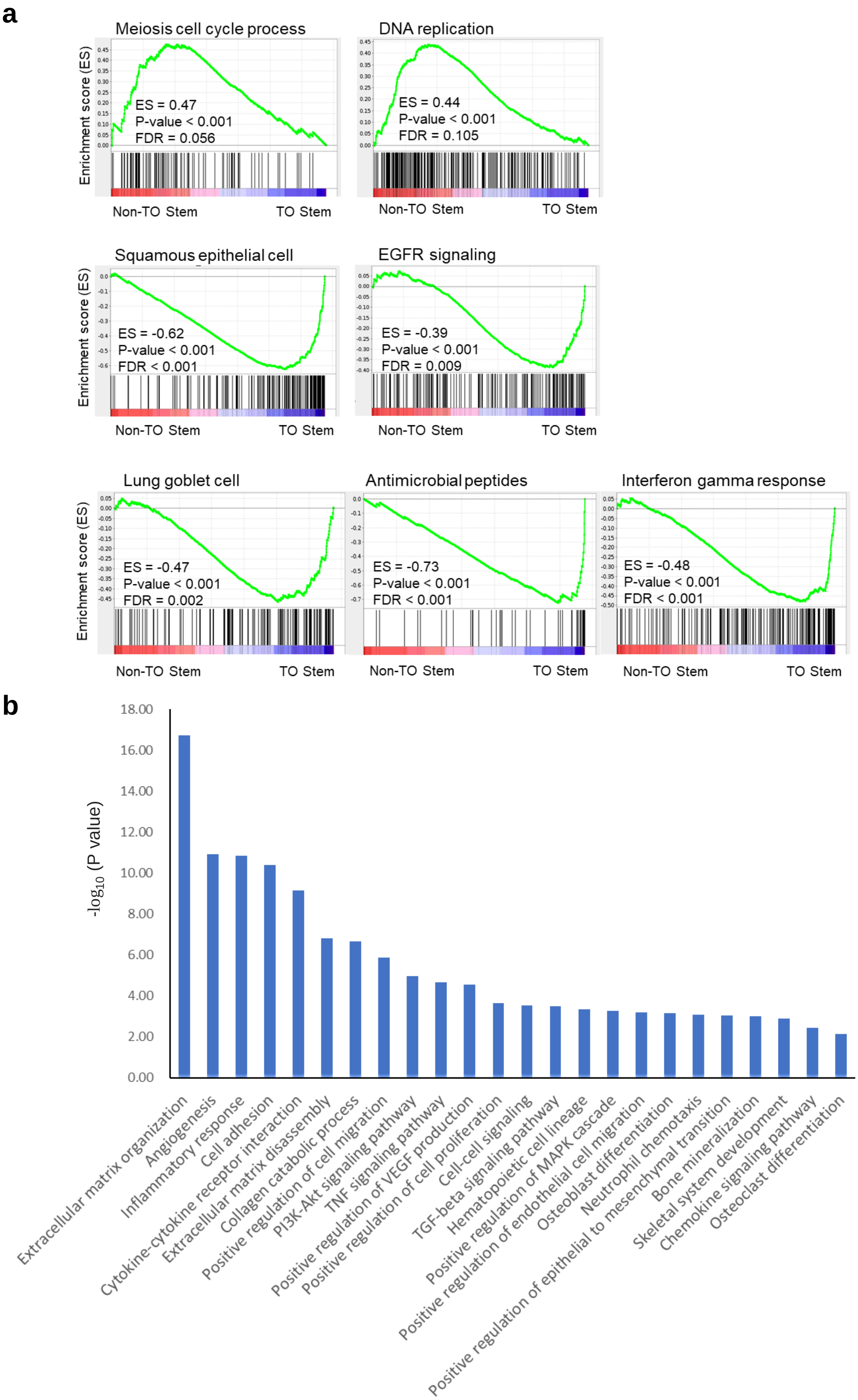


**Supplementary Figure 3. Enrichment analyses of RNA-Seq datasets generated from TBBC clones and corresponding ALI differentiated cells. (a)** Gene set enrichment analysis (GSEA) of basal cell RNA-Seq data. **(b)** GO and KEGG enrichment analysis of differentially expressing genes with further increment in severe TO cases (TO-02, 05, 06) compared to moderate cases (TO-01, -03, 04) according to ALI RNA-Seq data.

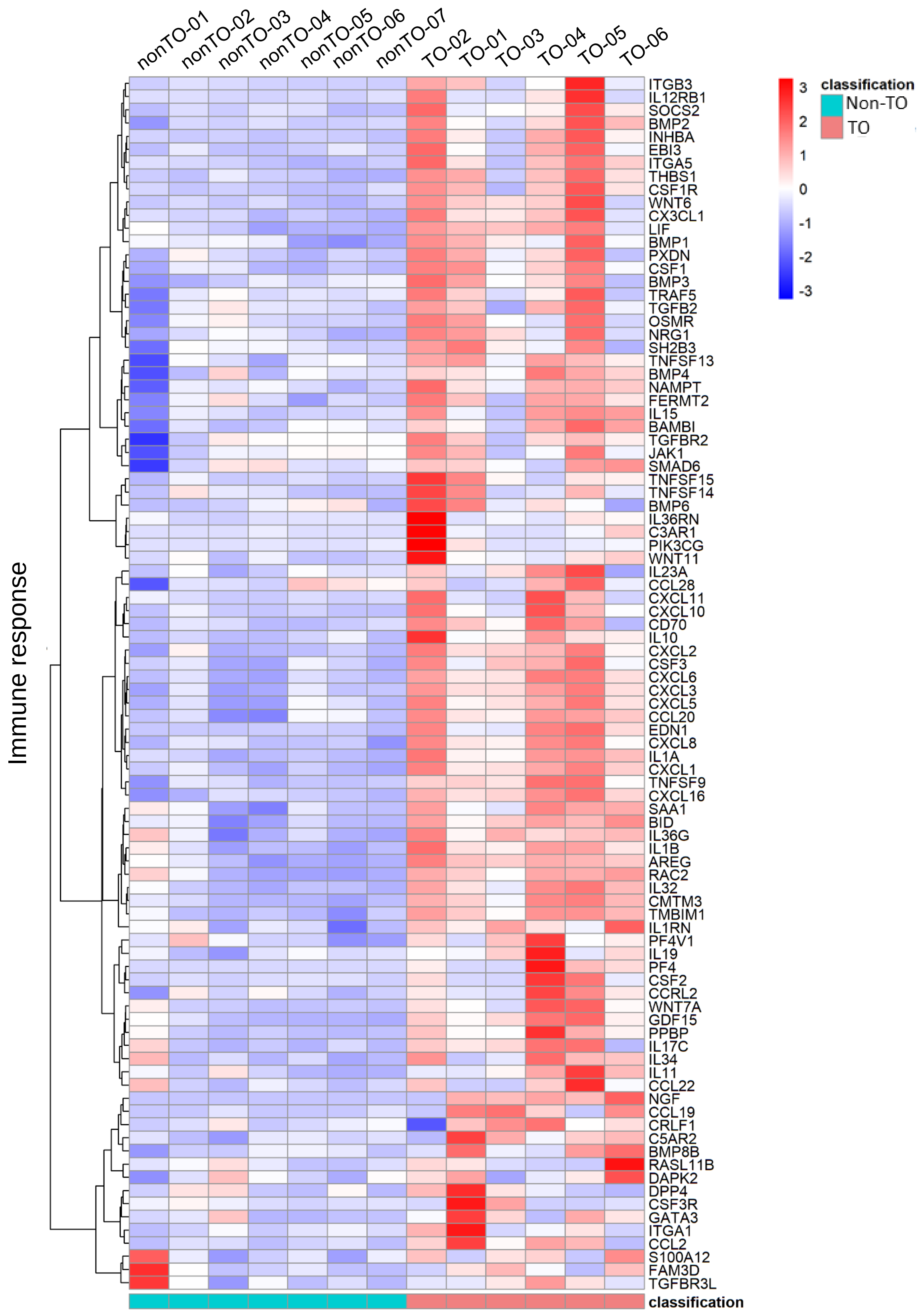


**Supplementary Figure 4. Comparative heatmap showing distinct expression profiles between non-TO and TO groups.** Differentially expressing genes involved in cytokine-cytokine receptor binding and chemotaxis events were selected to show in this heatmap across all non-TO controls and TO cases.

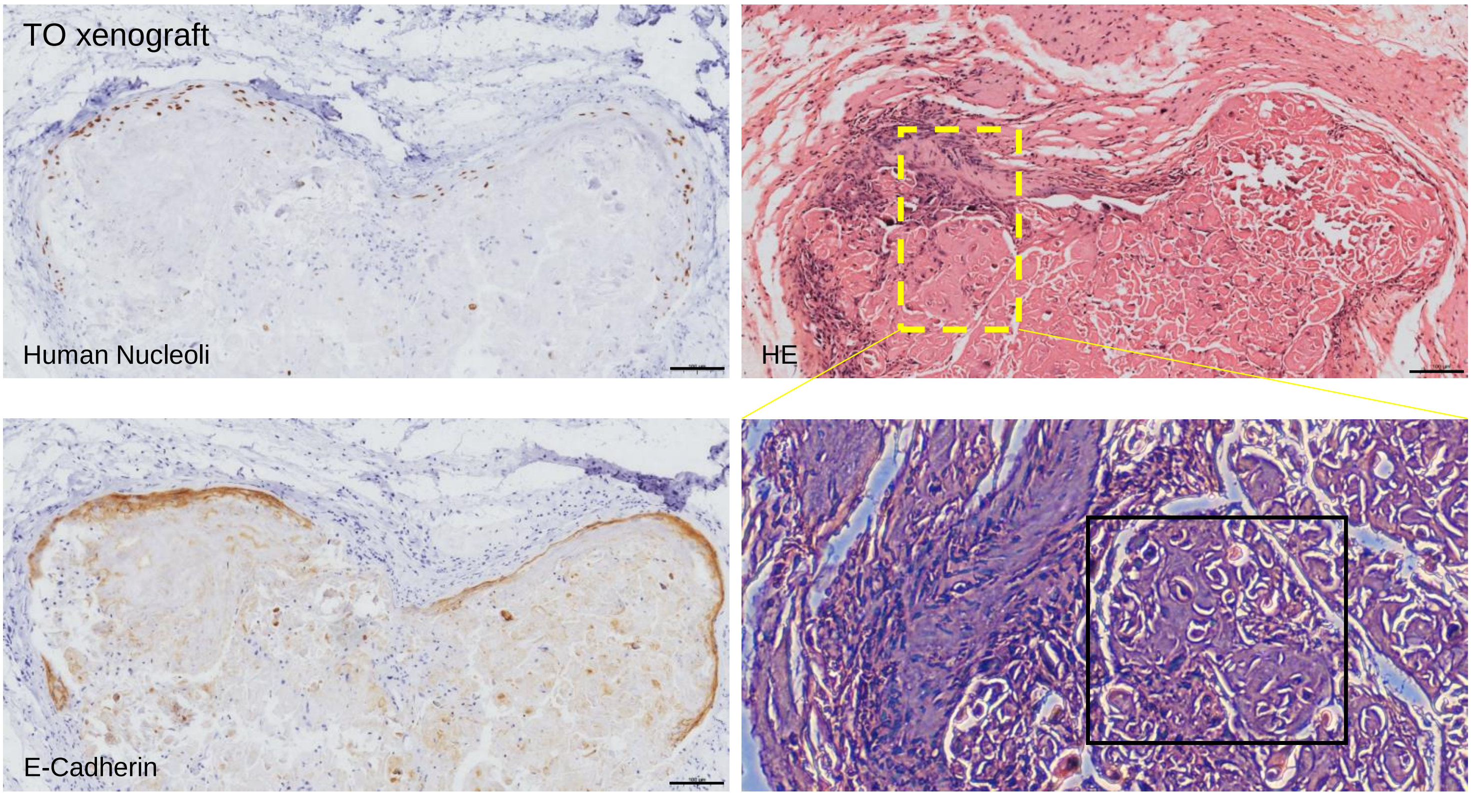


**Supplementary Figure 5. Histological sections showing a representative xenograft sample derived from TO-TBBCs transplantation, which contains a suspectable cartilaginous islet.** HE, immunohistochemical staining against anti-human nucleoli and E-Cadherin were performed on serial sections, to identify the transplanted epithelial portion. Yellow dotted box indicates a field that has been magnified into a separate image, and the black box is highlighting a cartilaginous-like islet. n=1 biological sample. Scale bar, 100µm.

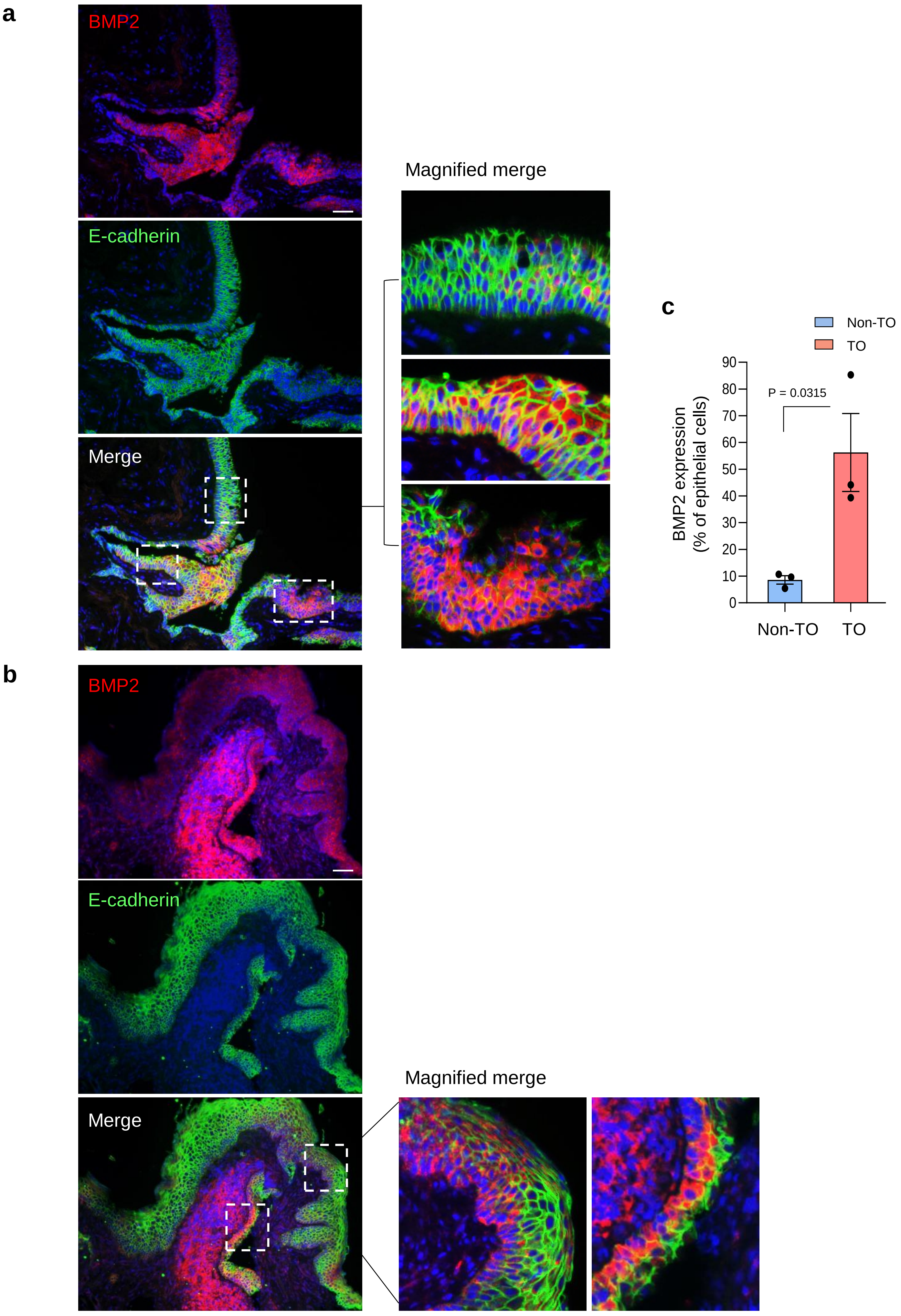
a
BMP2
E-cadherin
Merge
Magnified merge
b
BMP2
E-cadherin
Merge
Magnified merge
c
Non-TO
TO
BMP2 expression
(% of epithelial cells)
P = 0.0315
0
10
20
30
40
50
60
70
80
90
Non-TO
TO

**Supplementary Figure 6. Detection of BMP2 on patient-derived biopsies. (a-b)** Immunofluorescent images showing BMP2 tissue expression and distribution on paraffin sections from two TO cases. E-Cadherin was stained to locate epithelial compartment in biopsies. Magnified areas were highlighted in white dotted boxes and displayed in separate images. Scale bar, 100µm. **(c)** Quantification of BMP2 expression on tracheobronchial biopsy sections based on immunofluorescence results (non-TO n=3, TO n=3 biologically independent experiments). Results were shown as mean ± SD and statistical analysis was performed using two-tailed Student's t-test. P value is indicated to show statistical significance. Source data are provided as a Source Data file.

**a**

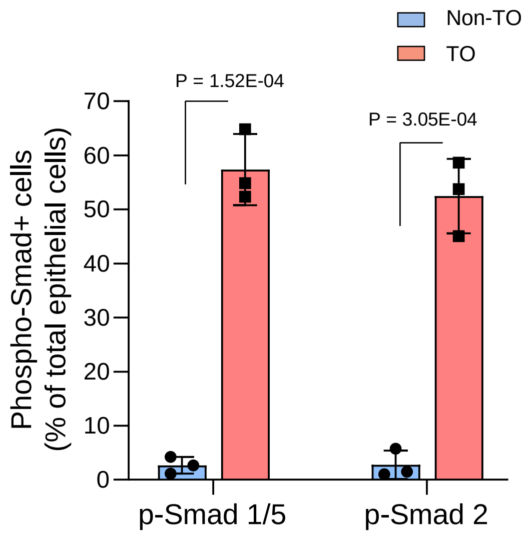


**b**

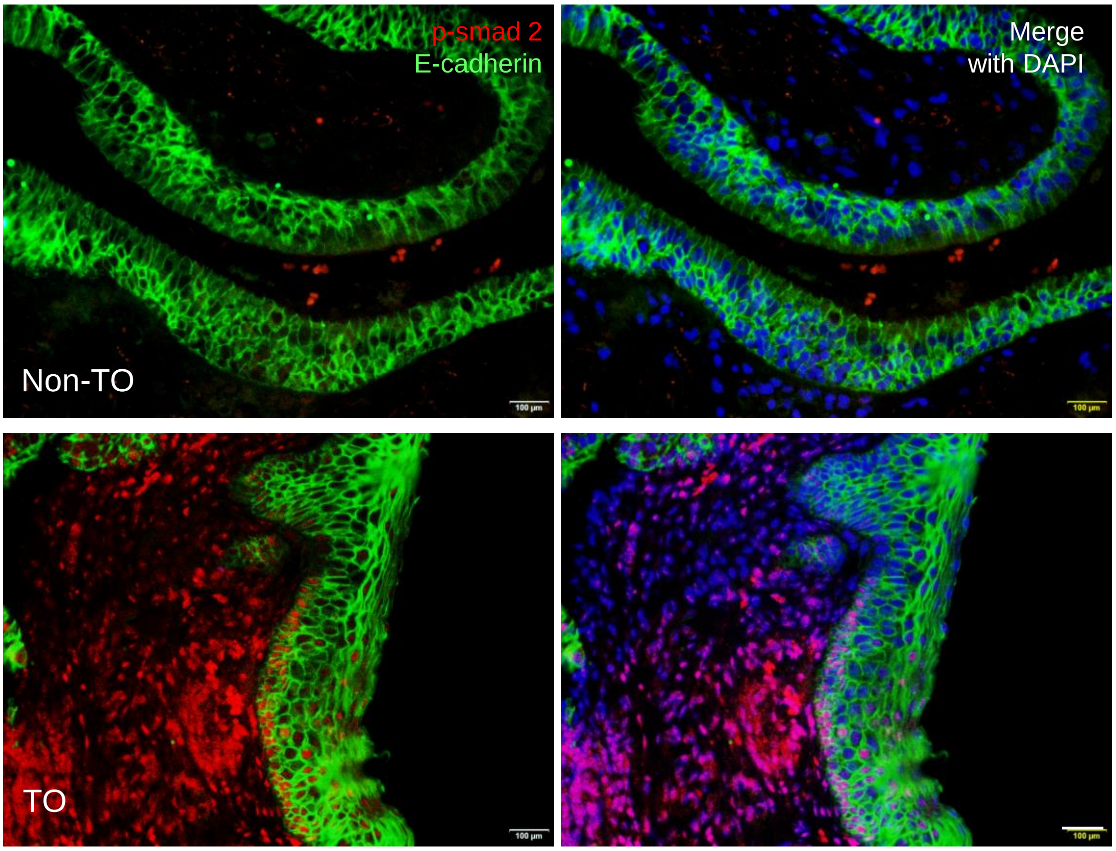


**Supplementary Figure 7. Detection of phosphorylated SMAD on patient-derived biopsies. (a)** Quantification of p-Smad2 and p-Smad1/5 in tissue epithelia based on immunofluorescence results (non-TO n=3, TO n=3 biologically independent experiments). Results were expressed as mean ± SD, and p values are indicated to show statistical significance based on two-tailed Student's t-tests. **(b)** Representative co-staining images showing p-Smad2 and E-Cadherin expression on patient biopsies that were proceeded with quantification analysis in (a). Scale bar, 50μm. Source data are provided as a Source Data file.

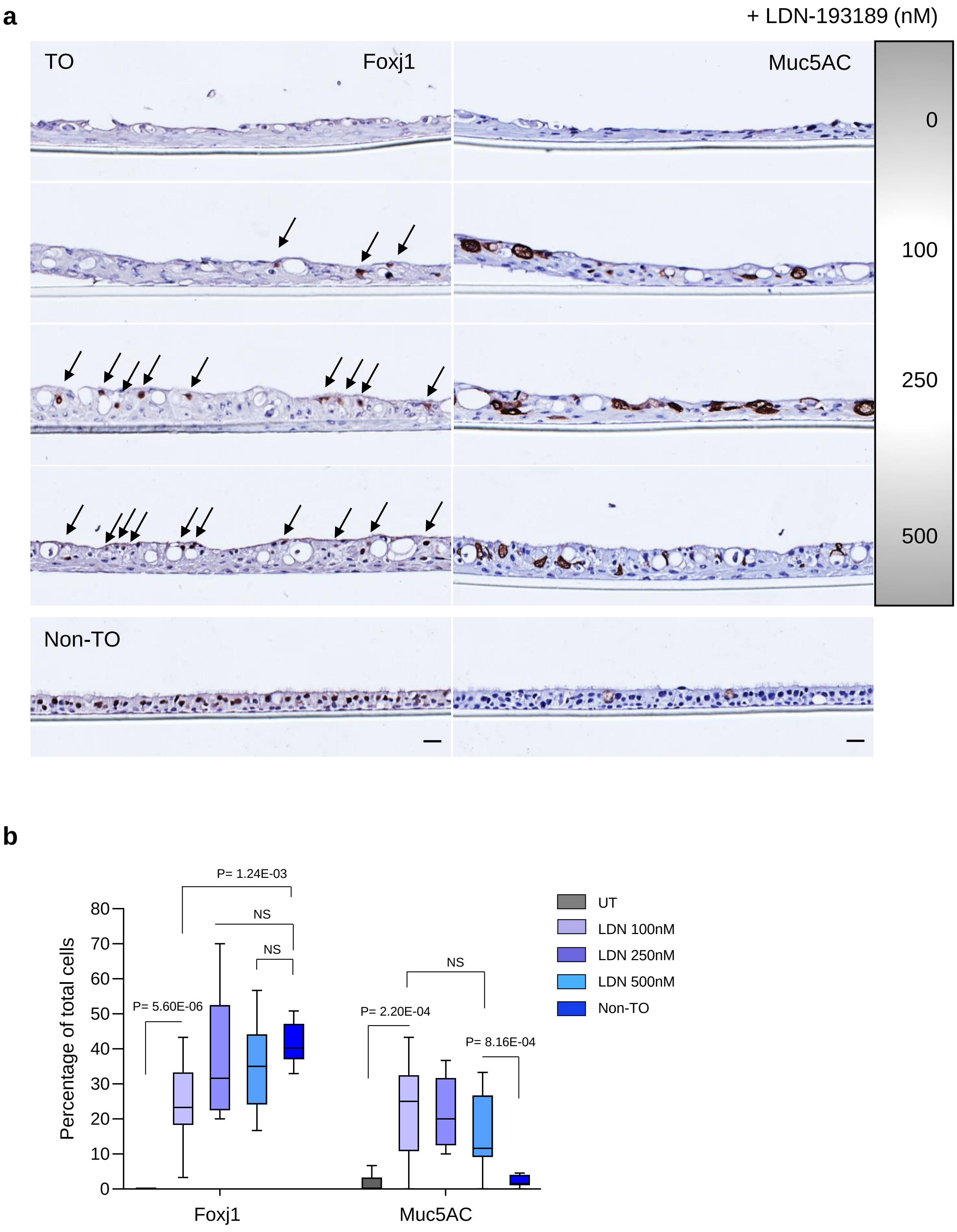
a
+ LDN-193189 (nM)
TO
Foxj1
Muc5AC
0
100
250
500
Non-TO
b
P= 1.24E-03
NS
NS
P= 5.60E-06
NS
P= 2.20E-04
P= 8.16E-04
Percentage of total cells
0
10
20
30
40
50
60
70
80
Foxj1
Muc5AC
UT
LDN 100nM
LDN 250nM
LDN 500nM
Non-TO

**Supplementary Figure 8. Effect of BMP inhibitor LDN-193189 on TO-basal cell differentiation.** **(a)** TO-derived basal cells were treated with LDN-193189 at concentrations ranging between 100-500nM since Day 0 of ALI differentiation. Day20 structures were fixed to proceed with paraffin sectioning and immunohistochemical staining for mucociliary markers Foxj1 and Muc5AC. ALIs of TO-derived and non-TO basal cells without inhibitor treatment were examined as controls. Foxj1+ cells were highlighted by black arrows on TO-ALI sections. Scale bar, 20μm. **(b)** Quantification of ciliated (Foxj1+) and goblet (Muc5AC+) cell frequencies on TO basal cell-derived ALIs. UT (untreated TO-ALI) and non-TO basal cell-derived ALI were examined as controls. Ten sections from 3 independent experiments were counted per group. Boxes denote 25th and 75th percentile, lines denote median and whiskers min-max. P values are indicated to show statistical significance ($^{NS}$ $p > 0.05$) based on two-tailed Student’s t-tests. Source data are provided as a Source Data file.

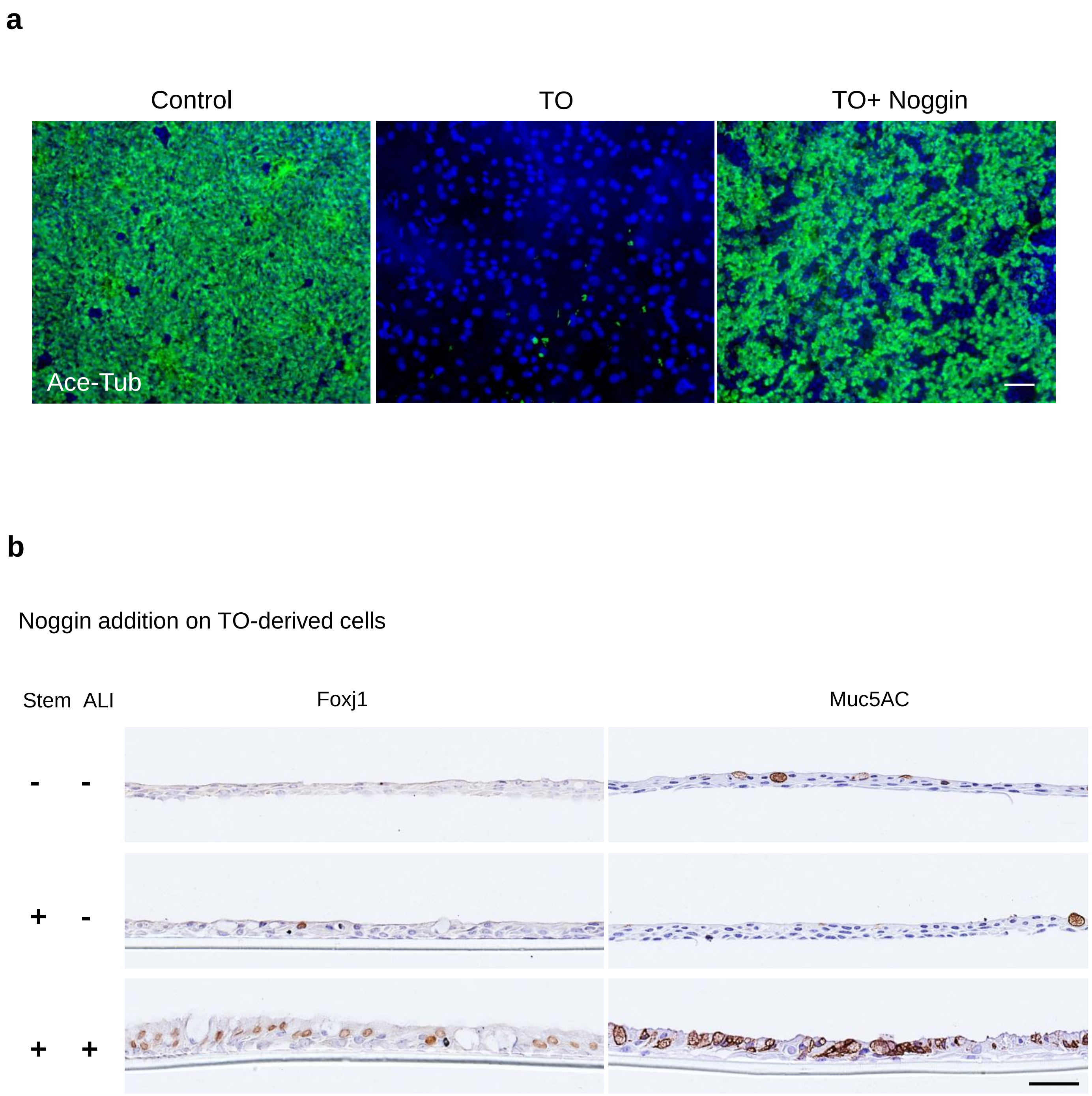


**Supplementary Figure 9. Effect of BMP antagonist Noggin on TO-basal cell differentiation. (a)** Representative ALI top-view images showing mucociliary differentiation of TO-derived basal cells under Noggin (200ng/ml) treatment since Day 0. TO-ALI without Noggin treatment and non-TO control ALI were examined in parallel. Day 20 structures were fixed to proceed with immunofluorescent staining (n=3 biologically independent experiments). Scale bar, 50μm. **(b)** An immunohistochemical comparison of Noggin on TO-TBBCs throughout ALI differentiation and Noggin withdrawal (n=2 biologically independent experiments). Differentiation efficiency was examined by Foxj1 and Muc5AC staining. Scale bar, 50μm.

**a**

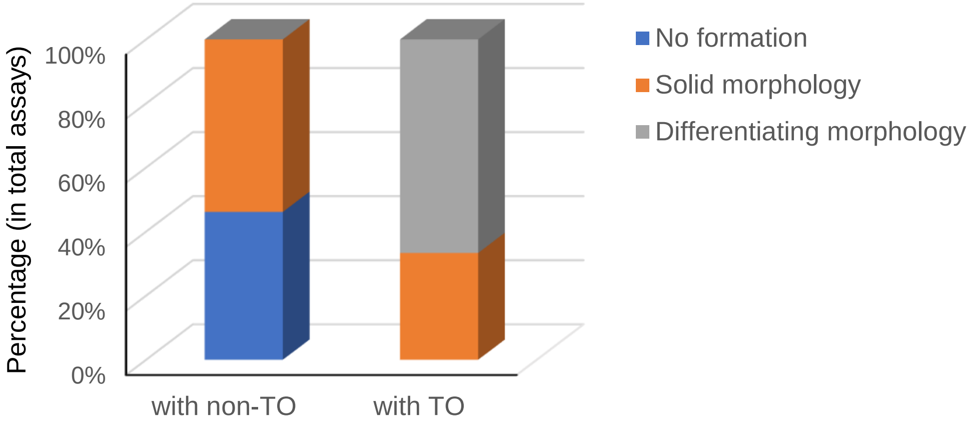


**b**

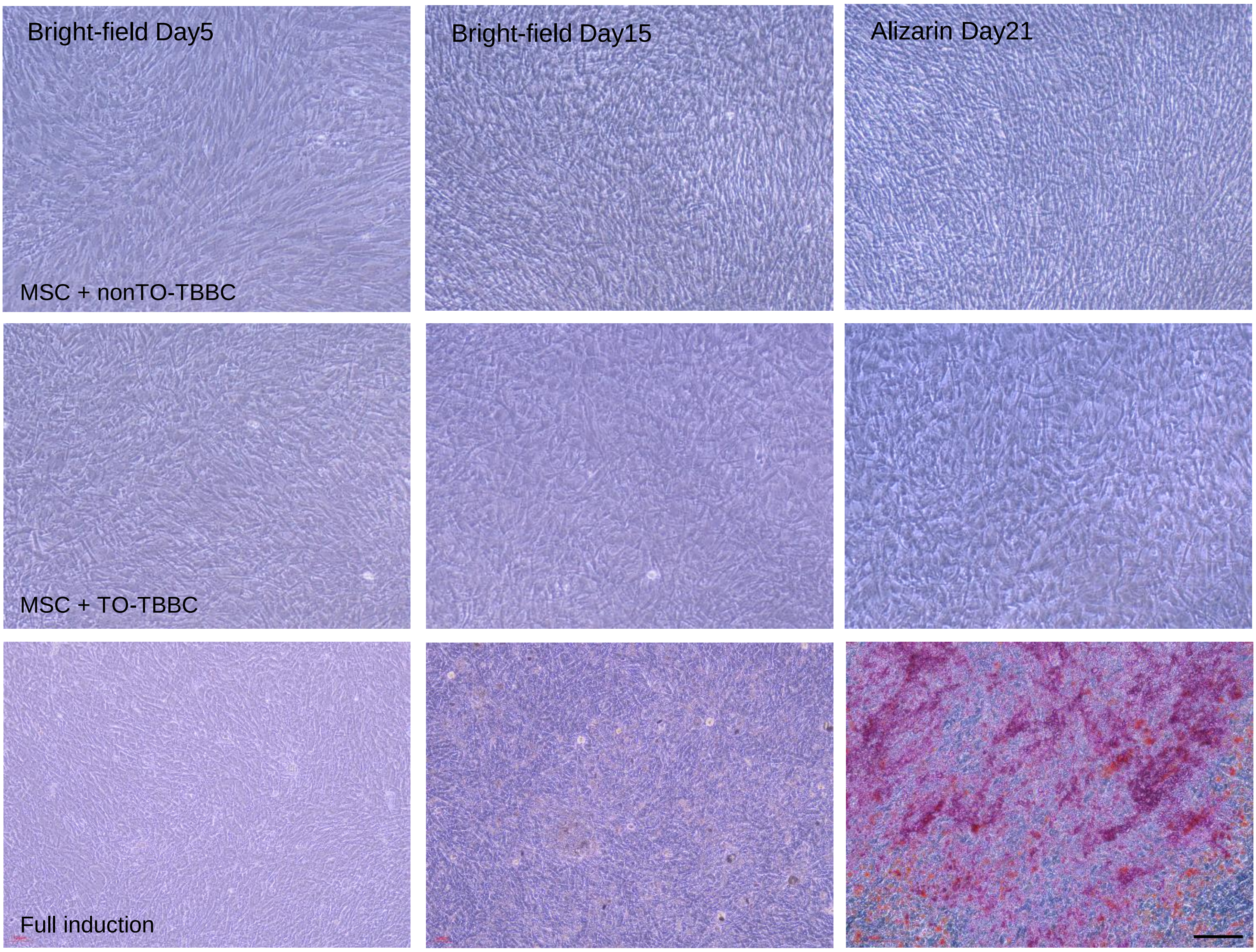


**Supplementary Figure 10. TBBC-MSC in vitro co-culture assays. (a)** A quantitative summary of MSC spheroid formation and differentiation based on observations from chondrogenic-lineage co-culture assays. Y-axis, percentage of total MSC pellets set for co-culture (non-TO n=13, TO n=7). Results were reached from two independent experiments involving a use of 3 non-TO cases and 2 TO-severe cases in examination. **(b)** A comparison of MSC osteogenic differentiation under co-culture conditions with non-TO controls and with TO-TBBCs derived ALI epithelia. MSCs undergone standard differentiation via full induction was examined in parallel. Calcium deposit was examined by Alizarin Red S Staining. Scale bar, 200µm. Independent experiments n=2 were performed for examination. Source data are provided as a Source Data file.

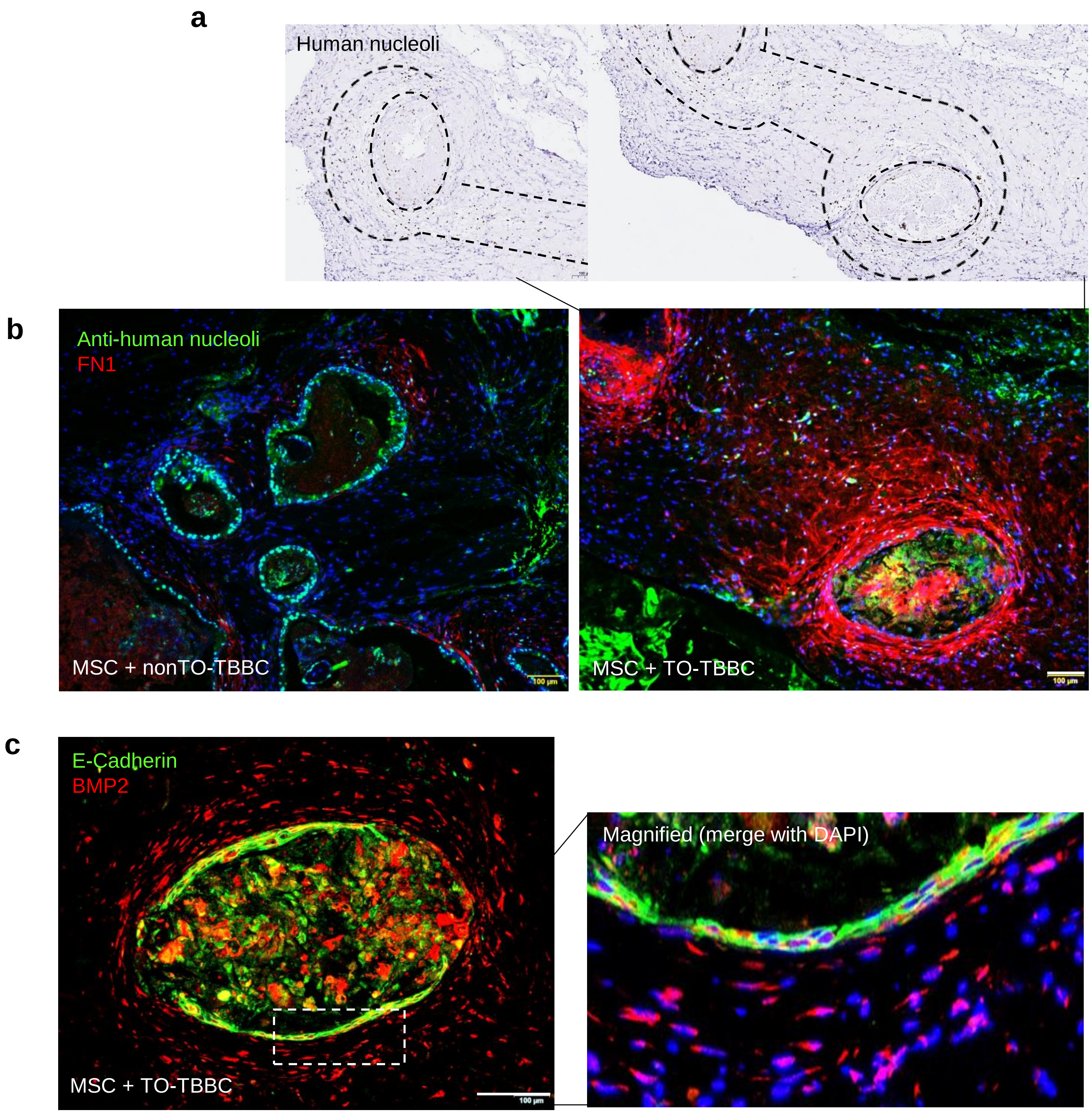


**Supplementary Figure 11. TBBC-MSC in vivo co-culture assays.** **(a)** Distribution of transplanted cells in xenografts was illustrated by human nucleoli staining, and circled in the pictures. **(b)** Immunofluorescent images showing FN1 expression in xenografts resulted from MSC growths when co-cultured with non-TO control cells or with TO-TBBCs. Scale bar, 100µm. **(c)** The serial sections of the same sets of xenografts were proceeded with BMP2 and E-Cadherin co-staining. Representative magnified image showing the area highlighted in white box. Scale bar, 100µm. Independent experiments n=2 were performed for examination. Source data are provided as a Source Data file.

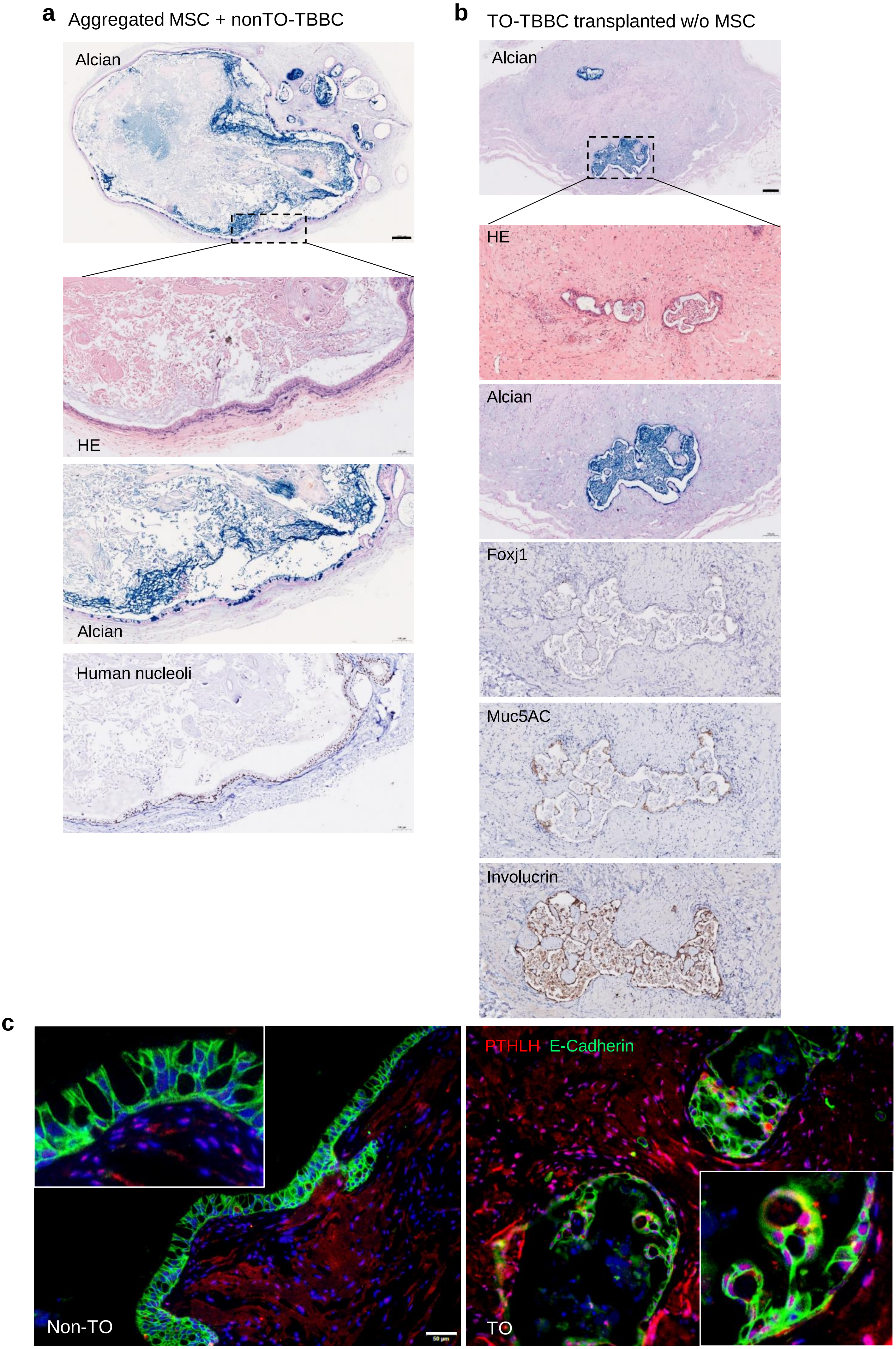
a
Aggregated MSC + nonTO-TBBC
Alcian
HE
Alcian
Human nucleoli
b
TO-TBBC transplanted w/o MSC
Alcian
HE
Alcian
Foxj1
Muc5AC
Involucrin
c
Non-TO
50 μm
PTHLH
E-Cadherin
TO

**Supplementary Figure 12. Controls for co-culture assay in vivo and PTHLH expression in non-TO and TO xenografts. (a)** Paraffin sections of 4-week xenografts formed by aggregated MSC spheroid + non-TO TBBC co-transplantation assay were examined by HE and Alcian blue staining. Human nucleoli distribution was confirmed by immunohistochemistry. Scale bar, 200µm. **(b)** Four-week xenografts formed by TO-TBBCs in the absence of co-transplanted MSC were examined by HE, Alcian blue and immunohistochemical staining for cell-type specific markers. Scale bar, 200µm. **(c)** Representative immunofluorescent images showing E-Cadherin and PTHLH co-staining on xeno-sections acquired from nonTO-TBBC and TO-TBBC transplantations. Independent experiments n= 2 were performed for examination. Scale bar, 50µm.

Supplementary Figure 13.

**PM phenotype: Normal-like**

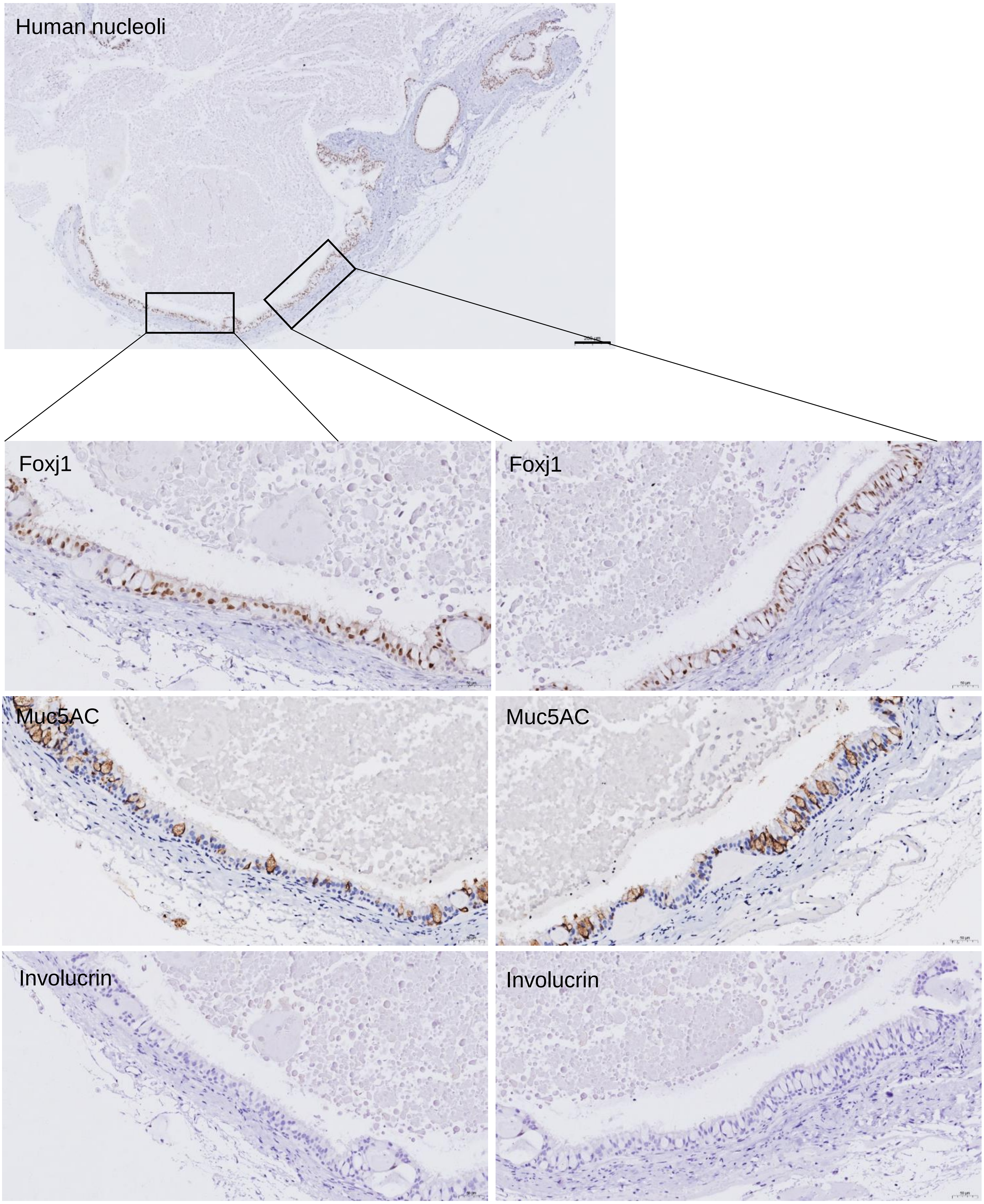

Supplementary Figure 14.

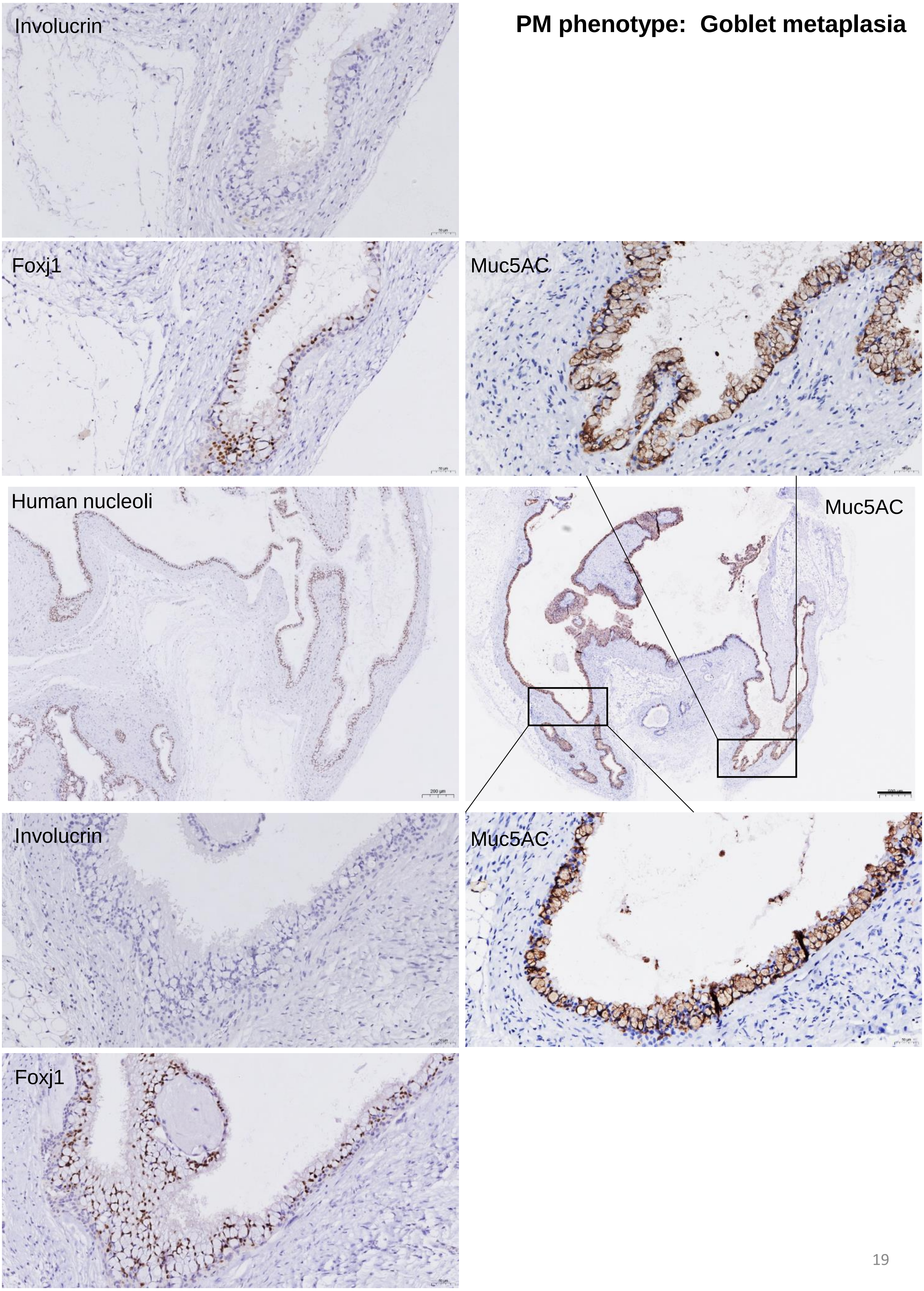

Supplementary Figure 15.

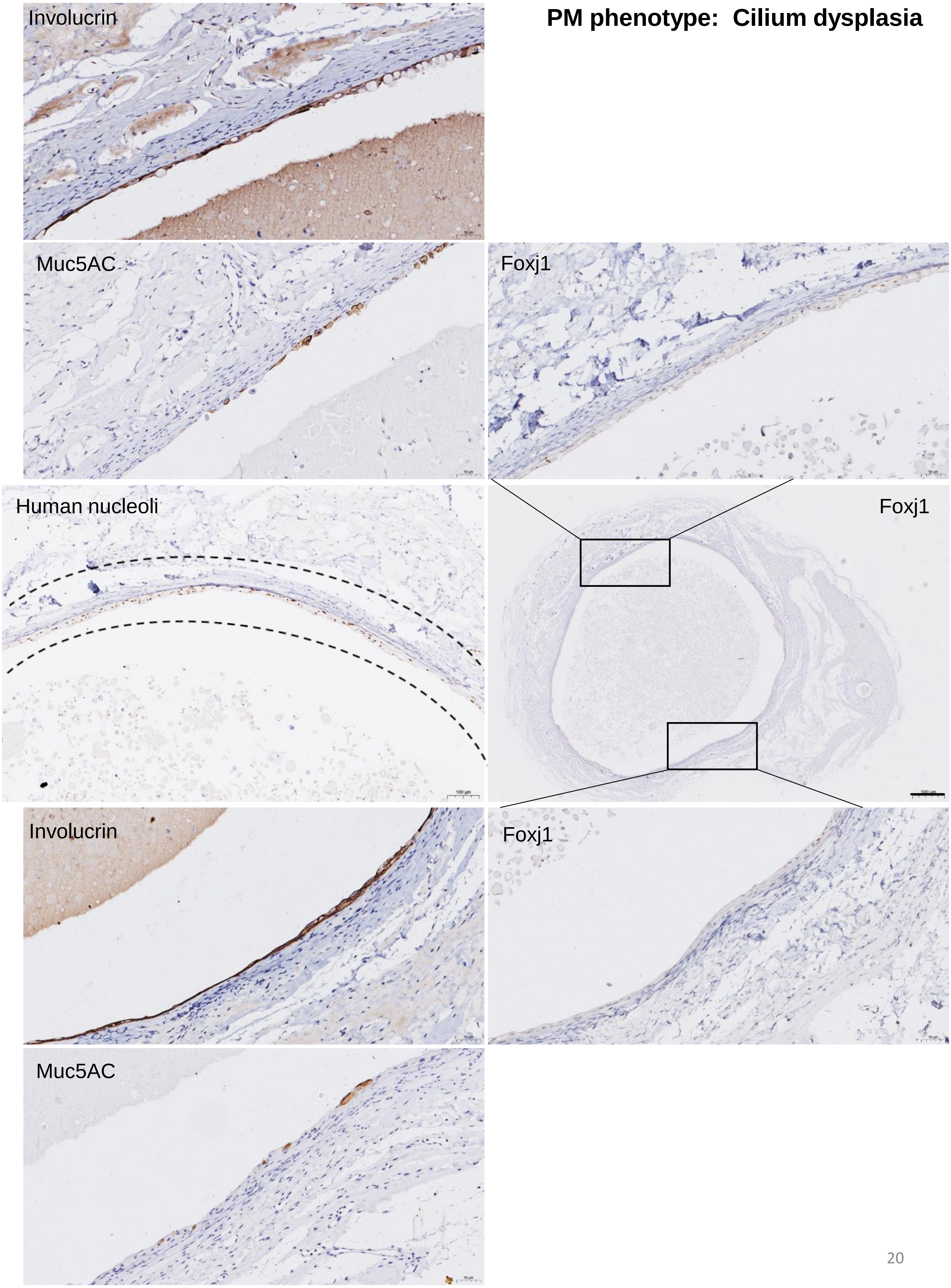

**Supplementary Figure 13-15. In vivo differentiation of patient-matched TBBCs isolated from the normal-looking regions of patients' trachea-bronchi.** Patient-matched (PM) TBBCs showed distinct differentiation potentials, referring to normal-like, goblet metaplasia and cilium dysplasia phenotypes observed on xenografts, which were collected at Week 4 post subcutaneous transplantation. Representative full-scale immunohistochemical images showing cell type frequencies using antibodies against Foxj1, Muc5AC and Involucrin (n=2 independent experiments). Human nucleoli antibody was used to confirm the location and distribution of transplanted cells. Scale bar, 500μm.

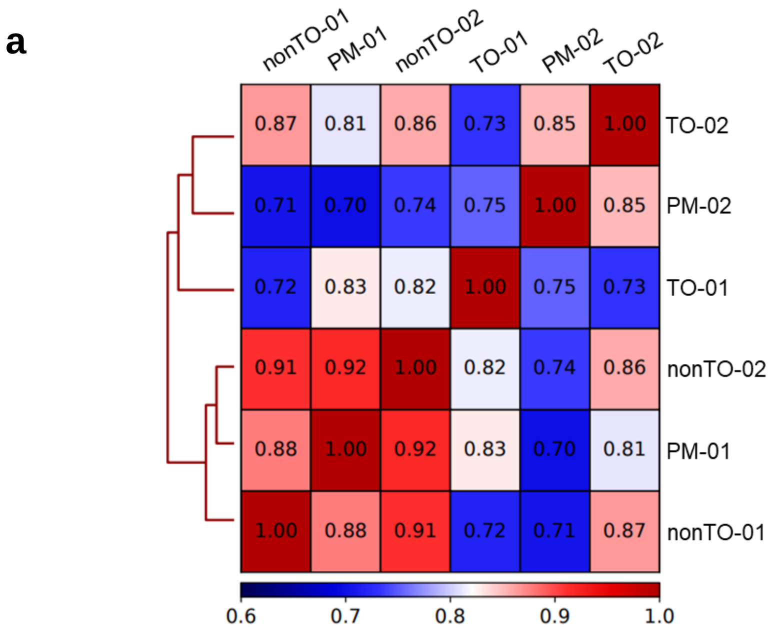


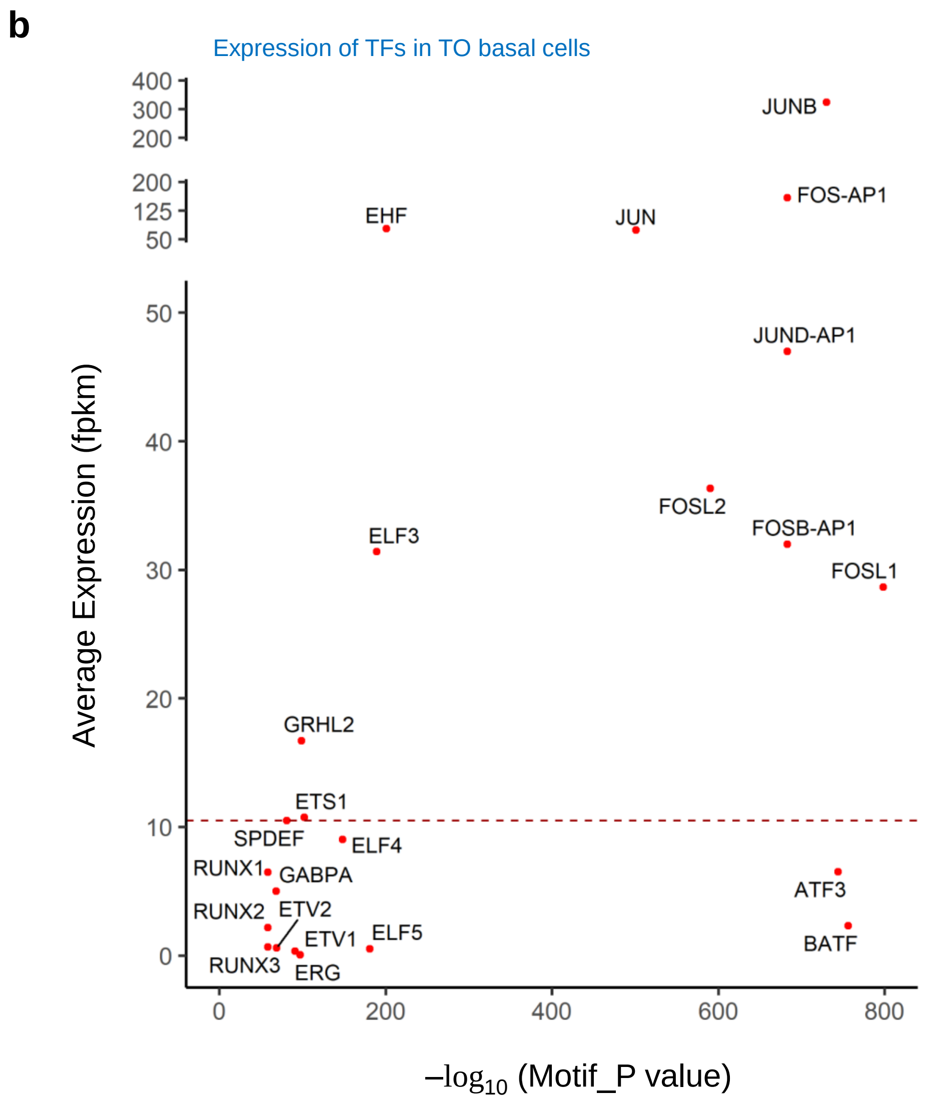


**Supplementary Figure 16. ATAC-Seq of patient-derived TBBCs and joint-analysis with RNA-Seq data. (a)** Pearson correlation result of ATAC-Seq data. **(b)** Correlation of motif enrichment with expression of corresponding transcription factors in TO-TBBCs. Red dotted line indicates the median value according to RNA-Seq data.

**a**

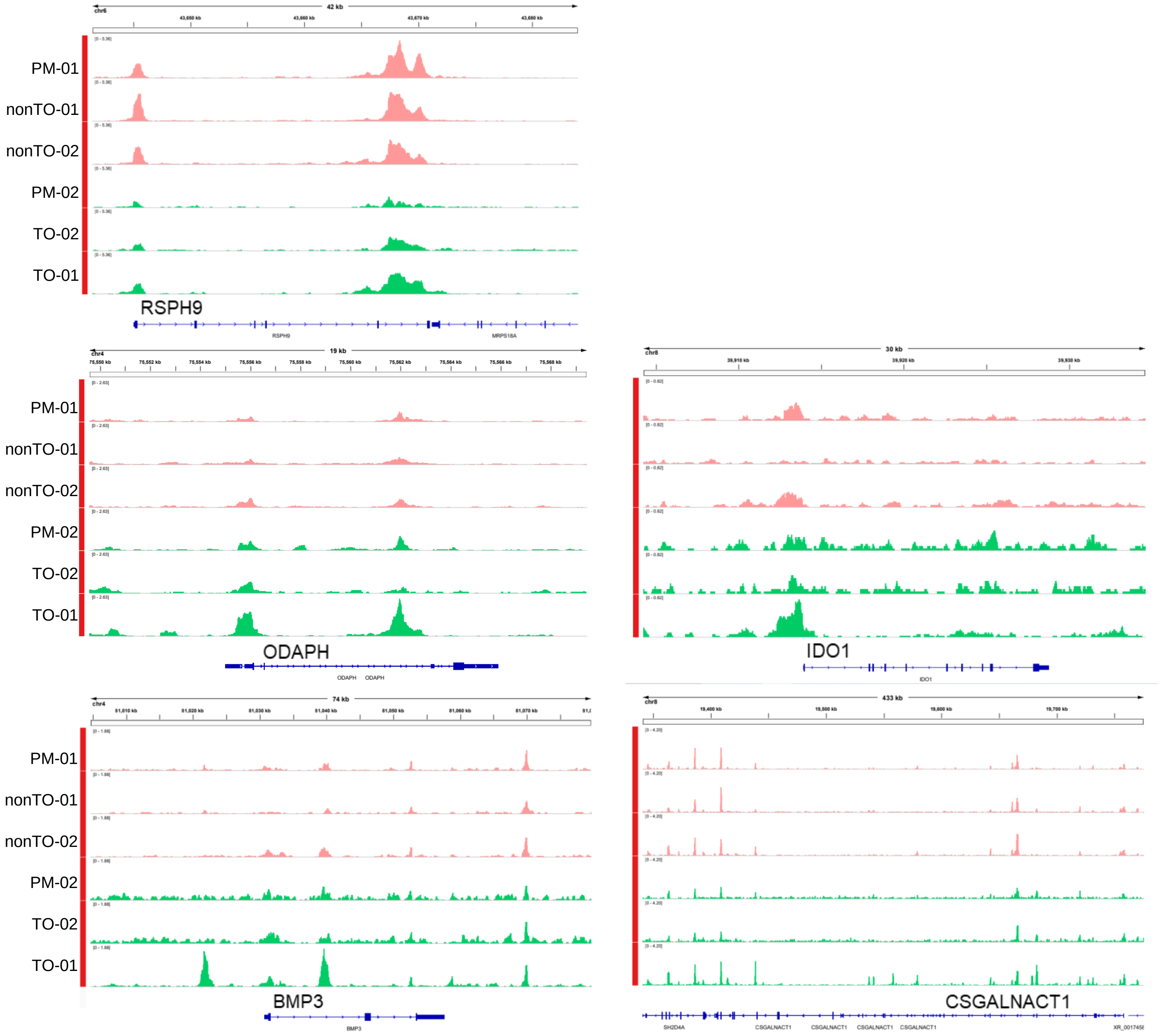

PM-01
nonTO-01
nonTO-02
PM-02
TO-02
TO-01
RSPH9
ODAPH
IDO1
BMP3
CSGALNACT1


**b**

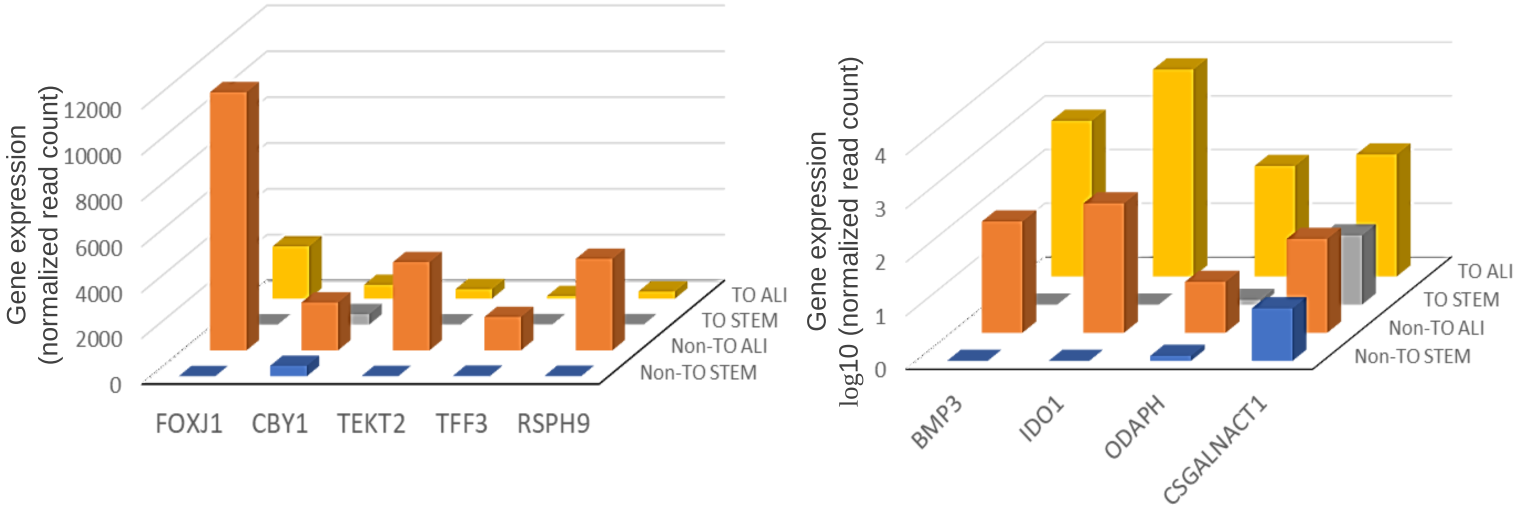

Gene expression (normalized read count)
12000
10000
8000
6000
4000
2000
0
FOXJ1
CBY1
TEKT2
TFF3
RSPH9
TO ALI
TO STEM
Non-TO ALI
Non-TO STEM
Gene expression log10 (normalized read count)
4
3
2
1
0
BMP3
IDO1
ODAPH
CSGALNACT1
TO ALI
TO STEM
Non-TO ALI
Non-TO STEM

**Supplementary Figure 17. Status of chromatin accessibility and gene expression at key loci.**
**(a)** IGV graphs visualizing chromatin accessibility at key loci related to cartilage development, inflammation and ciliogenesis. Peak height is positively correlated to accessibility. Genome scale is included and Y-axis is consistent for all loci. **(b)** Histogram showing transcriptional levels of selected genes between control and TO groups before and after differentiation, corresponding to IGV results shown in this figure and Fig. 7e.

**Supplementary Table 1** RNA-Seq data showing differentially expressing genes between non-TO and TO basal cells

| gene_id | log2FoldChange | pvalue | padj | nonTO-01 | nonTO-02 | nonTO-03 | nonTO-04 | nonTO-05 | nonTO-06 | nonTO-07 | TO-01 | TO-02 | TO-03 | TO-04 | TO-05 | TO-06 |
|---|---|---|---|---|---|---|---|---|---|---|---|---|---|---|---|---|
| VNN1 | -7.593689504 | 0.001346 | 0.160739 | 0 | 0 | 0 | 0 | 0 | 0 | 0 | 152.8883 | 9.497313 | 0 | 11.20352 | 20.37032 | 0 |
| PSG4 | -5.586023021 | 3.14E-05 | 0.013477 | 0 | 2.0028201 | 3.8646033 | 3.198896 | 1.042755 | 0.981502 | 0 | 234.0302 | 3.453568 | 1.215607 | 124.4835 | 91.06732 | 0 |
| FGF10 | -5.083834405 | 0.000425 | 0.072078 | 0 | 0 | 0 | 0 | 0 | 0 | 0 | 0.854125 | 12.08749 | 9.724853 | 7.469012 | 0 | 3.906223 |
| RPE65 | -5.076664789 | 6.31E-06 | 0.004376 | 0.6660006 | 1.3352134 | 0 | 0 | 1.042755 | 0 | 0 | 44.41449 | 10.3607 | 19.44971 | 4.979341 | 0 | 18.22904 |
| NELL2 | -4.829188551 | 7.87E-05 | 0.024935 | 0 | 0 | 0 | 0 | 1.042755 | 0 | 3.31977065 | 18.79075 | 1.726784 | 3.64682 | 3.734506 | 63.50747 | 5.208297 |
| PRKAR2B | -4.805790662 | 0.000414 | 0.071286 | 0 | 0 | 0 | 2.1325974 | 0 | 0 | 0 | 2.562374 | 4.31696 | 31.60577 | 4.979341 | 0 | 6.510371 |
| PHGR1 | -4.802798657 | 0.01146 | 0.4902 | 0 | 0 | 0 | 0 | 1.042755 | 0 | 0 | 30.74849 | 0 | 0 | 3.734506 | 0 | 2.604148 |
| ADAM23 | -4.504374426 | 0.000416 | 0.071286 | 1.3320013 | 0 | 0.9661508 | 2.1325974 | 0 | 0 | 1.10659022 | 11.10362 | 87.2026 | 0 | 1.244835 | 4.793017 | 1.302074 |
| EBF1 | -4.424426264 | 0.000121 | 0.033775 | 0 | 0 | 3.8646033 | 0 | 1.042755 | 0.981502 | 0 | 2.562374 | 34.53568 | 40.11502 | 18.67253 | 0 | 9.11452 |
| HGF | -4.418325715 | 1.67E-05 | 0.009005 | 0.6660006 | 0 | 0 | 0 | 2.08551 | 0.981502 | 1.10659022 | 0.854125 | 19.85802 | 34.03698 | 18.67253 | 2.396508 | 9.11452 |
| CRTAM | -4.414295992 | 0.018321 | 0.576919 | 0 | 1.3352134 | 0 | 0 | 0 | 0 | 0 | 20.49899 | 0 | 0 | 14.93802 | 2.396508 | 0 |
| HOXC13 | -4.387624369 | 1.26E-10 | 9.18E-07 | 5.3280051 | 2.0028201 | 0.9661508 | 2.1325974 | 0 | 0.981502 | 0 | 36.72737 | 87.2026 | 10.94046 | 33.61055 | 27.55985 | 24.73941 |
| MCOLN2 | -4.274412431 | 0.001992 | 0.200767 | 0 | 0 | 0 | 1.0662987 | 3.128265 | 0 | 1.10659022 | 0 | 39.71604 | 9.724853 | 31.12088 | 0 | 2.604148 |
| NPTX1 | -4.256513776 | 0.006266 | 0.358004 | 0 | 0 | 0 | 0 | 0 | 0 | 0 | 5.124749 | 1.726784 | 1.215607 | 8.713847 | 2.396508 | 0 |
| MCOLN3 | -4.194029849 | 0.000814 | 0.110386 | 0 | 0 | 0 | 4.2651947 | 0 | 1.963003 | 1.10659022 | 7.687123 | 68.20797 | 1.215607 | 27.38638 | 0 | 5.208297 |
| KLHL4 | -4.142050584 | 0.001665 | 0.183791 | 0 | 0.6676067 | 0 | 2.1325974 | 0 | 0 | 0 | 23.06137 | 4.31696 | 1.215607 | 3.734506 | 0 | 9.11452 |
| GNG2 | -4.096292202 | 0.002577 | 0.229754 | 0 | 0 | 0.9661508 | 0 | 0 | 0 | 0 | 6.832998 | 6.907137 | 1.215607 | 3.734506 | 0 | 3.906223 |
| KRT1 | -4.045199651 | 0.007413 | 0.388152 | 28.638027 | 4.6732468 | 0 | 0 | 0 | 2.944505 | 1.10659022 | 1.70825 | 56.98388 | 3.64682 | 13.69319 | 7.189525 | 451.8198 |
| S100A7 | -4.03813663 | 7.44E-06 | 0.004929 | 1.9980019 | 4.0056401 | 0.9661508 | 3.198896 | 1.042755 | 0.981502 | 3.31977065 | 138.3682 | 1.726784 | 1.215607 | 7.469012 | 41.9389 | 28.64563 |
| GREM2 | -4.016717832 | 0.005104 | 0.322914 | 0 | 0 | 0.9661508 | 0 | 0 | 0.981502 | 1.10659022 | 0 | 3.453568 | 19.44971 | 1.244835 | 0 | 15.62489 |
| CAPN13 | -4.006752312 | 0.00147 | 0.171316 | 0.6660006 | 0 | 1.9323017 | 4.2651947 | 0 | 5.88901 | 1.10659022 | 143.493 | 0 | 2.431213 | 29.87605 | 11.98254 | 0 |
| WT1 | -3.99390994 | 0.014682 | 0.53882 | 0 | 0 | 0.9661508 | 0 | 0 | 0 | 0 | 0 | 11.2241 | 4.862426 | 2.489671 | 0 | 2.604148 |
| PYDC1 | -3.759515658 | 0.014403 | 0.534801 | 0.6660006 | 0 | 0 | 0 | 0 | 1.963003 | 0 | 4.270624 | 0 | 1.215607 | 0 | 5.991271 | 20.83319 |
| CECR2 | -3.729710755 | 0.025751 | 0.665835 | 1.3320013 | 0.6676067 | 0 | 0 | 0 | 0 | 0 | 15.37425 | 6.043745 | 0 | 0 | 8.38778 | 0 |
| IL36B | -3.694174806 | 0.00363 | 0.270811 | 0 | 0 | 0 | 1.0662987 | 1.042755 | 0.981502 | 0 | 0 | 1.726784 | 1.215607 | 12.44835 | 5.991271 | 10.41659 |
| UBE2QL1 | -3.666566586 | 0.007444 | 0.388152 | 0 | 0.6676067 | 0 | 0 | 0 | 0.981502 | 0 | 0.854125 | 0.863392 | 6.078033 | 0 | 5.991271 | 9.11452 |
| EBI3 | -3.652583592 | 0.014232 | 0.534426 | 0 | 0 | 0.9661508 | 1.0662987 | 0 | 0 | 1.10659022 | 23.06137 | 0 | 1.215607 | 2.489671 | 3.594763 | 0 |
| CXCL11 | -3.594252613 | 0.001323 | 0.160118 | 1.3320013 | 4.6732468 | 0 | 0 | 1.042755 | 0 | 0 | 15.37425 | 1.726784 | 1.215607 | 49.79341 | 5.991271 | 5.208297 |
| MYL7 | -3.578196769 | 0.033845 | 0.739169 | 0.6660006 | 0 | 0 | 0 | 0 | 0 | 0 | 2.562374 | 3.453568 | 1.215607 | 8.713847 | 0 | 0 |
| WDR72 | -3.483206504 | 0.014078 | 0.531402 | 1.3320013 | 0 | 8.6953575 | 5.3314934 | 5.213776 | 0.981502 | 6.63954131 | 27.33199 | 0 | 4.862426 | 4.979341 | 231.2631 | 0 |
| NPY4R | -3.472453632 | 0.000349 | 0.065591 | 0 | 2.6704267 | 0.9661508 | 3.198896 | 0 | 0 | 0 | 9.395373 | 18.13123 | 4.862426 | 27.38638 | 2.396508 | 3.906223 |
| SLURP2 | -3.469626236 | 0.03742 | 0.765694 | 0 | 0 | 0 | 3.198896 | 0 | 0 | 0 | 0 | 3.453568 | 2.431213 | 0 | 7.189525 | 14.32282 |
| SPRR2B | -3.44266707 | 0.021855 | 0.620275 | 0 | 1.3352134 | 0 | 0 | 0 | 0 | 0 | 11.10362 | 1.726784 | 2.431213 | 0 | 1.198254 | 2.604148 |
| CACNG7 | -3.353293139 | 0.027515 | 0.675007 | 0 | 0 | 0 | 0 | 0 | 0.981502 | 1.10659022 | 0 | 3.453568 | 6.078033 | 7.469012 | 0 | 1.302074 |
| PI3 | -3.249264345 | 2.18E-07 | 0.00053 | 779.22075 | 920.62962 | 445.39553 | 1891.6139 | 555.7885 | 334.6921 | 1498.32316 | 5971.186 | 2090.272 | 1845.291 | 1744.014 | 7968.391 | 32751.07 |
| RUNX1T1 | -3.193022074 | 0.007459 | 0.388152 | 0 | 0.6676067 | 1.9323017 | 0 | 1.042755 | 2.944505 | 0 | 0 | 13.81427 | 20.66531 | 8.713847 | 0 | 7.812445 |
| SP140 | -3.19245536 | 0.039586 | 1 | 0 | 0 | 0 | 0 | 0 | 0 | 1.10659022 | 5.124749 | 0.863392 | 3.64682 | 1.244835 | 1.198254 | 0 |
| CASP14 | -3.152194279 | 0.039944 | 0.778063 | 9.3240089 | 0 | 0 | 0 | 0 | 0 | 1.10659022 | 31.60262 | 33.67229 | 0 | 2.489671 | 0 | 14.32282 |
| EMX2 | -3.098102785 | 0.009002 | 0.431743 | 0 | 0.6676067 | 0 | 0 | 2.08551 | 0.981502 | 3.31977065 | 0 | 14.67767 | 13.37167 | 8.713847 | 0 | 13.02074 |
| STUM | -3.096278246 | 0.030548 | 0.70536 | 0 | 1.3352134 | 1.9323017 | 0 | 1.042755 | 0 | 0 | 20.49899 | 0 | 0 | 7.469012 | 1.198254 | 2.604148 |
| ENPP2 | -3.090341113 | 0.003168 | 0.25644 | 0.6660006 | 0.6676067 | 0 | 1.0662987 | 1.042755 | 0 | 0 | 2.562374 | 6.043745 | 3.64682 | 2.489671 | 9.586034 | 1.302074 |
| OR7D2 | -3.055777747 | 0.01143 | 0.4902 | 0 | 0 | 1.9323017 | 2.1325974 | 1.042755 | 0 | 2.21318044 | 11.10362 | 15.54106 | 2.431213 | 0 | 20.37032 | 0 |
| CARD18 | -3.042366809 | 7.05E-07 | 0.001161 | 21.31202 | 6.6760669 | 11.59381 | 6.3977921 | 3.128265 | 4.907509 | 3.31977065 | 46.12274 | 49.21335 | 14.58728 | 51.03825 | 31.15461 | 220.0505 |
| PRRX1 | -3.037801856 | 0.000449 | 0.075194 | 3.3300032 | 11.349314 | 59.901352 | 0 | 11.47031 | 23.55604 | 11.0659022 | 24.76962 | 279.739 | 258.9242 | 144.4009 | 1.198254 | 139.3219 |
| FAP | -3.035703716 | 0.022544 | 0.62835 | 0 | 0 | 0 | 1.0662987 | 0 | 0.981502 | 0 | 4.270624 | 2.590176 | 1.215607 | 2.489671 | 0 | 3.906223 |
| SORCS1 | -3.022393399 | 0.007195 | 0.386028 | 0 | 0 | 3.8646033 | 0 | 0 | 0.981502 | 0 | 2.562374 | 5.180352 | 8.509246 | 4.979341 | 1.198254 | 10.41659 |
| HOXD8 | -3.017281237 | 0.002729 | 0.23949 | 0 | 0.6676067 | 10.627659 | 0 | 2.08551 | 0 | 1.10659022 | 3.416499 | 18.13123 | 26.74334 | 19.91736 | 1.198254 | 29.94771 |
| DEPP1 | -2.983712328 | 0.004227 | 0.287825 | 3.9960038 | 16.690167 | 28.018374 | 31.98896 | 4.171021 | 8.833516 | 16.5988533 | 572.2636 | 4.31696 | 7.293639 | 41.07956 | 118.6272 | 2.604148 |
| CALML5 | -2.95971017 | 3.35E-05 | 0.013553 | 45.288043 | 40.056401 | 19.323017 | 39.453051 | 51.095 | 18.64853 | 82.9942664 | 140.9306 | 39.71604 | 82.66125 | 185.4805 | 35.94763 | 1494.781 |
| ITGAX | -2.933885739 | 0.034502 | 0.739169 | 0 | 3.3380334 | 0.9661508 | 0 | 0 | 0 | 0 | 17.0825 | 6.907137 | 0 | 1.244835 | 3.594763 | 1.302074 |
| RAB37 | -2.902816715 | 0.001494 | 0.172804 | 9.9900096 | 16.690167 | 34.78143 | 11.729285 | 8.342041 | 6.870512 | 6.63954131 | 460.3733 | 2.590176 | 1.215607 | 104.5662 | 31.15461 | 9.11452 |
| COL6A3 | -2.899559707 | 0.017263 | 0.571147 | 0.6660006 | 0 | 0.9661508 | 0 | 0 | 70.66812 | 1.10659022 | 0 | 8.633921 | 6.078033 | 9.958682 | 4.793017 | 0 |
| SPRR2E | -2.898475777 | 1.39E-07 | 0.000406 | 46.620045 | 47.400075 | 22.221469 | 54.381233 | 14.59857 | 7.852014 | 101.8063 | 327.1298 | 237.4328 | 131.2855 | 95.85232 | 274.4002 | 817.7026 |
| BRINP3 | -2.873497801 | 0.002576 | 0.229754 | 0.6660006 | 2.6704267 | 16.424564 | 0 | 0 | 8.833516 | 3.31977065 | 12.81187 | 47.48656 | 64.42715 | 39.83473 | 0 | 35.156 |
| SFRP2 | -2.852244374 | 0.001538 | 0.176412 | 0 | 3.3380334 | 7.7292067 | 1.0662987 | 3.128265 | 8.833516 | 3.31977065 | 2.562374 | 73.38833 | 40.11502 | 26.14154 | 0 | 26.04148 |
| NEFL | -2.845606667 | 0.002586 | 0.229754 | 17.316017 | 9.3464936 | 0 | 4.2651947 | 3.128265 | 0 | 3.31977065 | 30.74849 | 86.33921 | 8.509246 | 41.07956 | 0 | 67.70786 |
| MAP1B | -2.806600233 | 0.001286 | 0.158848 | 404.92839 | 10.681707 | 3.8646033 | 2.1325974 | 1.042755 | 11.77802 | 0 | 161.4296 | 50.07674 | 4.862426 | 33.61055 | 4.793017 | 9.11452 |
| DLX1 | -2.803113261 | 0.00133 | 0.160118 | 23.976023 | 3.3380334 | 2.8984525 | 0 | 1.042755 | 0 | 0 | 5.124749 | 40.57943 | 10.94046 | 8.713847 | 4.793017 | 5.208297 |
| CACNA1G | -2.786246048 | 0.00017 | 0.042082 | 5.9940057 | 3.3380334 | 5.796905 | 0 | 1.042755 | 7.852014 | 0 | 11.10362 | 36.26247 | 51.05548 | 28.63121 | 3.594763 | 14.32282 |
| SDR9C7 | -2.764545733 | 0.00028 | 0.058748 | 9.3240089 | 4.6732468 | 1.9323017 | 4.2651947 | 2.08551 | 0.981502 | 3.31977065 | 13.666 | 32.8089 | 9.724853 | 7.469012 | 2.396508 | 92.44727 |
| GRIA3 | -2.759154098 | 0.000531 | 0.085199 | 0.6660006 | 5.3408535 | 15.458413 | 0 | 4.171021 | 7.852014 | 4.42636087 | 9.395373 | 62.16423 | 64.42715 | 56.01759 | 1.198254 | 26.04148 |
| DEFB1 | -2.757917506 | 0.004077 | 0.284231 | 0 | 0.6676067 | 0 | 13.861883 | 9.384796 | 9.815017 | 4.42636087 | 43.56036 | 0.863392 | 59.56472 | 13.69319 | 8.38778 | 92.44727 |
| COMP | -2.719225077 | 0.03124 | 0.714557 | 1.9980019 | 1.3352134 | 4.8307542 | 0 | 0 | 1.963003 | 0 | 28.18612 | 3.453568 | 0 | 24.89671 | 1.198254 | 0 |
| PTPRD | -2.715943901 | 0.000544 | 0.085199 | 37.962036 | 47.400075 | 1.9323017 | 8.5303895 | 38.58194 | 36.31556 | 1.10659022 | 51.24749 | 489.5433 | 91.17049 | 7.469012 | 115.0324 | 216.1443 |
| MSX2 | -2.699979488 | 0.00014 | 0.037085 | 4.6620045 | 13.352134 | 0.9661508 | 11.729285 | 6.256531 | 0 | 4.42636087 | 46.97686 | 18.99463 | 24.31213 | 3.734506 | 53.92144 | 84.63482 |
| TBX15 | -2.688538236 | 0.006131 | 0.353503 | 0.6660006 | 0 | 7.7292067 | 0 | 2.08551 | 0.981502 | 4.42636087 | 3.416499 | 15.54106 | 35.25259 | 13.69319 | 0 | 18.22904 |
| EBF3 | -2.680499695 | 0.008597 | 0.421721 | 0 | 0 | 3.8646033 | 0 | 0 | 3.926007 | 3.31977065 | 1.70825 | 10.3607 | 20.66531 | 13.69319 | 1.198254 | 11.71867 |
| BPIFC | -2.676565947 | 0.026143 | 0.668244 | 7.9920076 | 0.6676067 | 0 | 0 | 0 | 0.981502 | 0 | 0.854125 | 3.453568 | 6.078033 | 11.20352 | 1.198254 | 33.85393 |
| IL32 | -2.630408133 | 0.000366 | 0.066639 | 7.9920076 | 17.357774 | 55.070597 | 4.2651947 | 2.08551 | 5.88901 | 11.0659022 | 217.8018 | 6.043745 | 26.74334 | 119.5042 | 46.73192 | 134.1136 |
| SULT4A1 | -2.620979606 | 0.019346 | 0.586008 | 5.9940057 | 4.6732468 | 0 | 0 | 0 | 1.963003 | 0 | 30.74849 | 4.31696 | 8.509246 | 21.1622 | 0 | 5.208297 |
| POTEI | -2.615875448 | 0.007158 | 0.386028 | 0 | 0 | 3.8646033 | 2.1325974 | 1.042755 | 1.963003 | 1.10659022 | 0 | 6.907137 | 17.01849 | 17.42769 | 3.594763 | 6.510371 |
| HERC5 | -2.614316669 | 0.006076 | 0.353503 | 11.988011 | 2.0028201 | 1.9323017 | 2.1325974 | 1.042755 | 0.981502 | 4.42636087 | 7.687123 | 2.590176 | 0 | 84.6488 | 29.95636 | 6.510371 |
| HIST1H4E | -2.543372395 | 0.015462 | 0.553512 | 0.6660006 | 2.6704267 | 3.8646033 | 0 | 0 | 0.981502 | 1.10659022 | 27.33199 | 3.453568 | 1.215607 | 9.958682 | 4.793017 | 0 |
| SLC19A3 | -2.538232033 | 0.014001 | 0.530288 | 0.6660006 | 2.0028201 | 0.9661508 | 0 | 0 | 0.981502 | 0 | 5.978873 | 1.726784 | 2.431213 | 11.20352 | 2.396508 | 1.302074 |
| IGFL1 | -2.533573748 | 0.002027 | 0.200767 | 12.654012 | 10.681707 | 42.510637 | 13.861883 | 4.171021 | 2.944505 | 9.95931196 | 29.89437 | 6.907137 | 7.293639 | 57.26242 | 19.17207 | 360.6746 |
| KRT6C | -2.520851397 | 0.004491 | 0.300276 | 1910.0898 | 697.64899 | 307.23596 | 106.62987 | 245.0475 | 281.691 | 422.717463 | 752.4839 | 3992.325 | 265.0022 | 1196.287 | 407.4064 | 12925.69 |
| KRT6B | -2.496787503 | 0.000137 | 0.037012 | 9583.0832 | 7995.9253 | 2566.0966 | 1518.4093 | 1918.669 | 1536.05 | 7149.6794 | 11861.23 | 16741.17 | 6691.914 | 14070.37 | 4709.139 | 102042.3 |
| SPINK7 | -2.483502449 | 0.000162 | 0.04073 | 62.60406 | 56.746568 | 212.55318 | 178.07188 | 205.4228 | 45.14908 | 192.546698 | 327.1298 | 25.90176 | 414.5218 | 341.0849 | 995.7493 | 2462.222 |
| TNFSF4 | -2.477428005 | 3.94E-05 | 0.014717 | 13.320013 | 22.698627 | 3.8646033 | 18.127078 | 12.51306 | 18.64853 | 11.0659022 | 39.28974 | 297.0069 | 29.17456 | 56.01759 | 20.37032 | 36.45808 |
| CCL17 | -2.457823428 | 0.02536 | 0.661072 | 1.9980019 | 0 | 3.8646033 | 0 | 3.128265 | 0.981502 | 0 | 16.22837 | 0 | 2.431213 | 12.44835 | 14.37905 | 1.302074 |
| PSCA | -2.453460191 | 0.048866 | 0.855164 | 18.648018 | 71.433915 | 3556.4012 | 1766.8569 | 238.7909 | 182.5593 | 243.449848 | 17682.09 | 19.85802 | 161.6757 | 4075.591 | 6095.519 | 499.9965 |
| CALHM5 | -2.450773713 | 0.018852 | 0.581949 | 0 | 3.3380334 | 1.9323017 | 2.1325974 | 0 | 0 | 0 | 5.978873 | 6.043745 | 1.215607 | 16.18286 | 1.198254 | 5.208297 |
| SPRR2A | -2.449379245 | 7.96E-06 | 0.00504 | 90.576087 | 111.49032 | 24.153771 | 77.839804 | 67.77908 | 78.52014 | 172.628074 | 1207.732 | 158.0008 | 188.419 | 185.4805 | 159.3678 | 1016.92 |
| BICC1 | -2.447127345 | 0.027005 | 0.672647 | 1.9980019 | 0 | 1.9323017 | 0 | 0 | 0.981502 | 0 | 2.562374 | 5.180352 | 6.078033 | 3.734506 | 0 | 6.510371 |
| RPTN | -2.406150895 | 0.00481 | 0.312497 | 0.6660006 | 16.02256 | 109.17504 | 47.983441 | 16.68408 | 15.70403 | 3.31977065 | 393.7515 | 7.770529 | 18.2341 | 270.1293 | 131.808 | 128.9053 |
| LCE3E | -2.404954181 | 0.016247 | 0.567925 | 7.326007 | 10.681707 | 3.8646033 | 4.2651947 | 0 | 0 | 1.10659022 | 74.30886 | 4.31696 | 17.01849 | 6.224176 | 23.96508 | 0 |
| LCE3D | -2.35575746 | 0.011756 | 0.491417 | 29.970029 | 9.3464936 | 0.9661508 | 26.657467 | 0 | 0 | 16.5988533 | 196.4487 | 22.44819 | 60.78033 | 13.69319 | 67.10224 | 6.510371 |
| CYP4F22 | -2.355181513 | 0.000396 | 0.070286 | 10.65601 | 6.0084602 | 3.8646033 | 69.309414 | 34.41092 | 4.907509 | 6.63954131 | 78.57948 | 68.20797 | 23.09653 | 97.09715 | 89.86907 | 235.6754 |
| ZEB2 | -2.354421319 | 0.003022 | 0.249163 | 2.6640025 | 18.025381 | 54.104447 | 1.0662987 | 17.72684 | 18.64853 | 6.63954131 | 8.541248 | 166.6347 | 128.8543 | 120.749 | 3.594763 | 92.44727 |
| S100A8 | -2.338686104 | 0.000146 | 0.037879 | 1541.1255 | 1632.2983 | 2301.3713 | 6422.317 | 2628.786 | 1961.04 | 4330.08752 | 13136.44 | 1804.489 | 5464.152 | 3870.193 | 9007.277 | 56973.56 |
| NRCAM | -2.32222105 | 0.049772 | 0.855164 | 23.310022 | 4.0056401 | 0.9661508 | 3.198896 | 1.042755 | 0 | 3.31977065 | 58.08048 | 67.34458 | 0 | 28.63121 | 0 | 1.302074 |
| CXCL10 | -2.322083089 | 0.033567 | 0.736561 | 2.6640025 | 4.0056401 | 0 | 2.1325974 | 0 | 0 | 1.10659022 | 11.10362 | 1.726784 | 0 | 18.67253 | 1.198254 | 11.71867 |
| SPRR2D | -2.32078011 | 0.000133 | 0.036483 | 290.37628 | 237.66798 | 116.90425 | 364.67415 | 295.0997 | 770.4789 | 1004.78392 | 3626.614 | 215.848 | 829.0437 | 1309.567 | 1109.583 | 6096.311 |
| MRGPRX3 | -2.319925004 | 0.003643 | 0.270811 | 8.6580083 | 47.400075 | 8.6953575 | 3.198896 | 0 | 0.981502 | 5.53295109 | 118.7233 | 64.75441 | 30.39016 | 65.97627 | 10.78429 | 32.55186 |

Continue on the next page…

| gene_id | log2FoldChange | pvalue | padj | nonTO-01 | nonTO-02 | nonTO-03 | nonTO-04 | nonTO-05 | nonTO-06 | nonTO-07 | TO-01 | TO-02 | TO-03 | TO-04 | TO-05 | TO-06 |
|---|---|---|---|---|---|---|---|---|---|---|---|---|---|---|---|---|
| S100A12 | -2.305434901 | 0.000277 | 0.058748 | 3.3300032 | 18.025381 | 10.627659 | 23.458571 | 5.213776 | 10.79652 | 23.2383946 | 146.0553 | 12.08749 | 12.15607 | 44.81407 | 35.94763 | 148.4365 |
| ZFP42 | -2.284652026 | 0.028078 | 0.679555 | 0.6660006 | 2.0028201 | 1.9323017 | 0 | 0 | 0.981502 | 1.10659022 | 11.10362 | 6.907137 | 1.215607 | 1.244835 | 0 | 7.812445 |
| KRT77 | -2.274731678 | 0.001645 | 0.18358 | 1.3320013 | 2.0028201 | 3.8646033 | 2.1325974 | 5.213776 | 1.963003 | 4.42636087 | 14.52012 | 0 | 13.37167 | 7.469012 | 19.17207 | 31.24978 |
| HIST2H3D | -2.261885011 | 0.002911 | 0.249163 | 0.6660006 | 1.3352134 | 0 | 6.3977921 | 0 | 2.944505 | 1.10659022 | 14.52012 | 5.180352 | 6.078033 | 9.958682 | 8.38778 | 5.208297 |
| KRTDAP | -2.259921427 | 0.003619 | 0.270811 | 101.2321 | 10.681707 | 6.7630558 | 33.055259 | 13.55582 | 16.68553 | 30.9845261 | 10.2495 | 239.1596 | 48.62426 | 27.38638 | 35.94763 | 515.6214 |
| CCL20 | -2.25571701 | 0.001425 | 0.16882 | 5.9940057 | 65.425455 | 59.901352 | 28.790064 | 19.81235 | 16.68553 | 34.3042968 | 627.7817 | 23.31159 | 23.09653 | 95.85232 | 43.13715 | 131.5095 |
| MMP1 | -2.25379055 | 0.004316 | 0.291145 | 124.54212 | 2445.4433 | 24.153771 | 13.861883 | 36.49643 | 11.77802 | 78.5679055 | 1005.305 | 94.97313 | 19.44971 | 265.1499 | 9.586034 | 158.8531 |
| MYO7A | -2.25211353 | 0.00056 | 0.086777 | 7.326007 | 2.6704267 | 9.6615083 | 1.0662987 | 1.042755 | 2.944505 | 0 | 15.37425 | 9.497313 | 9.724853 | 24.89671 | 10.78429 | 33.85393 |
| HEPHL1 | -2.247701889 | 0.027007 | 0.672647 | 93.90609 | 82.783229 | 10.627659 | 9.5966881 | 3.128265 | 0 | 3.31977065 | 295.5272 | 257.2908 | 44.97744 | 69.71078 | 34.74937 | 128.9053 |
| MARCO | -2.245280091 | 0.003862 | 0.27518 | 0.6660006 | 4.0056401 | 11.59381 | 22.392272 | 1.042755 | 0 | 11.0659022 | 77.72535 | 9.497313 | 13.37167 | 31.12088 | 27.55985 | 44.27052 |
| PCDHB16 | -2.244125012 | 0.017359 | 0.572219 | 1.3320013 | 0.6676067 | 0.9661508 | 2.1325974 | 1.042755 | 0 | 0 | 5.124749 | 5.180352 | 7.293639 | 2.489671 | 4.793017 | 0 |
| CLDN17 | -2.237393904 | 0.002095 | 0.203973 | 5.3280051 | 10.0141 | 6.7630558 | 14.928182 | 32.32541 | 5.88901 | 11.0659022 | 40.99799 | 7.770529 | 36.4682 | 17.42769 | 8.38778 | 236.9775 |
| APOL1 | -2.215663776 | 2.45E-05 | 0.011167 | 133.20013 | 465.32186 | 710.12086 | 609.92285 | 481.7529 | 396.5267 | 414.971332 | 7424.053 | 1251.919 | 1072.165 | 1440.274 | 1082.024 | 513.0172 |
| TUBA1A | -2.190694677 | 7.17E-07 | 0.001161 | 160.50615 | 120.1692 | 51.205994 | 31.98896 | 54.22327 | 118.7617 | 55.3295109 | 743.0886 | 249.5203 | 249.1993 | 497.9341 | 116.2307 | 467.4446 |
| TLR2 | -2.178445648 | 0.000356 | 0.065591 | 7.9920076 | 4.6732468 | 3.8646033 | 7.4640908 | 3.128265 | 11.77802 | 12.1724924 | 105.9115 | 31.94551 | 14.58728 | 19.91736 | 16.77556 | 6.510371 |
| TMPRSS11B | -2.169322634 | 0.000395 | 0.070286 | 64.602062 | 17.357774 | 16.424564 | 50.116038 | 39.6247 | 11.77802 | 38.7306576 | 399.7304 | 8.633921 | 79.01443 | 159.3389 | 61.11097 | 212.2381 |
| PDE3B | -2.167296745 | 0.004037 | 0.283068 | 2.6640025 | 12.01692 | 4.8307542 | 17.060779 | 11.47031 | 3.926007 | 13.2790826 | 18.79075 | 3.453568 | 24.31213 | 4.979341 | 167.7556 | 31.24978 |
| MAP6 | -2.158214975 | 0.018163 | 0.576919 | 0.6660006 | 5.3408535 | 4.8307542 | 1.0662987 | 1.042755 | 0.981502 | 0 | 20.49899 | 2.590176 | 6.078033 | 14.93802 | 0 | 10.41659 |
| NHLH2 | -2.153095577 | 0.007529 | 0.390365 | 12.654012 | 14.01974 | 0.9661508 | 7.4640908 | 2.08551 | 0.981502 | 4.42636087 | 72.60061 | 34.53568 | 10.94046 | 22.40704 | 23.96508 | 0 |
| GLDN | -2.147150104 | 0.018013 | 0.576919 | 2.6640025 | 0.6676067 | 3.8646033 | 5.3314934 | 16.68408 | 0 | 2.21318044 | 28.18612 | 9.497313 | 40.11502 | 0 | 34.74937 | 5.208297 |
| HIST1H1D | -2.137072629 | 0.047917 | 0.848751 | 1.3320013 | 0.6676067 | 0.9661508 | 1.0662987 | 0 | 0.981502 | 0 | 8.541248 | 4.31696 | 1.215607 | 0 | 3.594763 | 1.302074 |
| FER1L6 | -2.132673462 | 0.039567 | 0.777398 | 76.590073 | 53.408535 | 87.919726 | 26.657467 | 54.22327 | 40.24157 | 15.4922631 | 1032.637 | 1.726784 | 23.09653 | 174.2769 | 97.05859 | 3.906223 |
| KRT79 | -2.118456656 | 0.00723 | 0.386028 | 0 | 2.0028201 | 3.8646033 | 5.3314934 | 2.08551 | 0.981502 | 2.21318044 | 23.06137 | 1.726784 | 2.431213 | 16.18286 | 9.586034 | 6.510371 |
| IL33 | -2.112316324 | 0.003807 | 0.274565 | 18.648018 | 83.450836 | 28.018374 | 14.928182 | 15.64133 | 58.8901 | 21.0252141 | 586.7837 | 116.5579 | 21.88092 | 115.7697 | 8.38778 | 42.96845 |
| EEF1A2 | -2.110523784 | 0.008975 | 0.431743 | 23.976023 | 2.0028201 | 28.984525 | 102.36467 | 18.76959 | 20.61154 | 8.85272174 | 398.8763 | 1.726784 | 131.2855 | 49.79341 | 135.4027 | 41.66637 |
| HIST1H2BG | -2.103579813 | 1.58E-05 | 0.008871 | 15.984015 | 18.025381 | 19.323017 | 26.657467 | 15.64133 | 12.75952 | 28.7713457 | 198.1569 | 24.17498 | 19.44971 | 126.9732 | 57.5162 | 76.82238 |
| WFDC12 | -2.092273832 | 7.12E-05 | 0.023064 | 12.654012 | 6.0084602 | 4.8307542 | 8.5303895 | 3.128265 | 3.926007 | 7.74613153 | 42.70624 | 9.497313 | 41.33062 | 6.224176 | 39.54239 | 33.85393 |
| PGLYRP4 | -2.086664476 | 4.11E-06 | 0.003326 | 17.982017 | 18.025381 | 83.088972 | 56.51383 | 33.36816 | 52.01959 | 21.0252141 | 214.3853 | 92.38295 | 82.66125 | 136.9319 | 117.4289 | 380.2057 |
| SLC24A3 | -2.084743317 | 0.014012 | 0.530288 | 13.986013 | 1.3352134 | 0.9661508 | 11.729285 | 2.08551 | 0.981502 | 1.10659022 | 15.37425 | 8.633921 | 6.078033 | 4.979341 | 74.29176 | 9.11452 |
| FPR1 | -2.073123893 | 0.049544 | 0.855164 | 1.9980019 | 0 | 3.8646033 | 5.3314934 | 0 | 0 | 3.31977065 | 30.74849 | 1.726784 | 1.215607 | 11.20352 | 4.793017 | 1.302074 |
| TRABD2A | -2.072935979 | 0.02671 | 0.672647 | 1.3320013 | 5.3408535 | 1.9323017 | 1.0662987 | 0 | 0 | 3.31977065 | 21.35312 | 7.770529 | 2.431213 | 9.958682 | 5.991271 | 0 |
| C5AR2 | -2.067882489 | 0.005635 | 0.336498 | 1.9980019 | 3.3380334 | 15.458413 | 9.5966881 | 9.384796 | 6.870512 | 6.63954131 | 76.01711 | 3.453568 | 13.37167 | 13.69319 | 80.28303 | 2.604148 |
| DAPK1 | -2.061710582 | 0.007099 | 0.385943 | 17.982017 | 14.01974 | 102.41199 | 29.856363 | 22.94061 | 106.0022 | 7.74613153 | 490.2676 | 24.17498 | 14.58728 | 117.0145 | 391.8291 | 37.76015 |
| OASL | -2.053787481 | 0.00536 | 0.328129 | 8.6580083 | 27.371874 | 67.630558 | 49.049739 | 55.26602 | 24.53754 | 109.552432 | 92.24548 | 19.85802 | 14.58728 | 724.4941 | 301.9601 | 62.49956 |
| ANO4 | -2.05311295 | 0.033212 | 0.735475 | 5.9940057 | 17.357774 | 6.7630558 | 3.198896 | 5.213776 | 0 | 6.63954131 | 70.89236 | 42.30621 | 14.58728 | 33.61055 | 0 | 0 |
| VIM | -2.051821727 | 2.31E-05 | 0.010842 | 6111.2218 | 324.45685 | 267.62378 | 125.82324 | 53.18051 | 258.135 | 126.151285 | 620.9487 | 1358.979 | 1051.5 | 337.3504 | 654.2468 | 2063.788 |
| AADAC | -2.03580173 | 0.039812 | 0.777398 | 6.6600064 | 16.690167 | 0 | 1.0662987 | 5.213776 | 19.63003 | 2.21318044 | 7.687123 | 2.590176 | 21.88092 | 3.734506 | 3.594763 | 143.2282 |
| ELFN1 | -2.035459146 | 0.003034 | 0.249163 | 4.6620045 | 0.6676067 | 3.8646033 | 2.1325974 | 1.042755 | 0.981502 | 2.21318044 | 4.270624 | 6.907137 | 15.80289 | 2.489671 | 9.586034 | 16.92696 |
| ANXA10 | -2.033969579 | 0.026844 | 0.672647 | 1.9980019 | 28.039481 | 18.356866 | 3.198896 | 7.299286 | 0.981502 | 4.42636087 | 135.8058 | 2.590176 | 12.15607 | 11.20352 | 63.50747 | 1.302074 |
| LIPK | -2.033188428 | 0.003615 | 0.270811 | 13.320013 | 4.0056401 | 2.8984525 | 3.198896 | 1.042755 | 2.944505 | 7.74613153 | 28.18612 | 3.453568 | 8.509246 | 19.91736 | 7.189525 | 58.59334 |
| PCDHGA1 | -2.032580156 | 0.008704 | 0.424122 | 3.3300032 | 1.3352134 | 2.8984525 | 1.0662987 | 4.171021 | 0 | 0 | 7.687123 | 6.907137 | 3.64682 | 18.67253 | 2.396508 | 6.510371 |
| DHRS2 | -2.030912794 | 0.013081 | 0.51512 | 2.6640025 | 5.3408535 | 3.8646033 | 4.2651947 | 1.042755 | 0 | 4.42636087 | 9.395373 | 11.2241 | 0 | 38.58989 | 13.1808 | 3.906223 |
| GABRQ | -2.017745796 | 0.032601 | 0.730773 | 1.9980019 | 0.6676067 | 0.9661508 | 2.1325974 | 1.042755 | 0 | 1.10659022 | 3.416499 | 11.2241 | 1.215607 | 7.469012 | 0 | 3.906223 |
| ATP6V0A4 | -2.012375845 | 0.003009 | 0.249163 | 2.6640025 | 4.6732468 | 23.18762 | 36.254155 | 17.72684 | 16.68553 | 12.1724924 | 174.2415 | 2.590176 | 29.17456 | 56.01759 | 52.72319 | 74.21823 |
| CSNK1A1L | -2.012340479 | 0.044981 | 0.836779 | 0 | 0.6676067 | 15.458413 | 1.0662987 | 2.08551 | 6.870512 | 0 | 0.854125 | 32.8089 | 23.09653 | 16.18286 | 2.396508 | 14.32282 |
| SLC10A6 | -2.006230831 | 0.000256 | 0.057522 | 66.600064 | 38.053581 | 23.18762 | 34.121558 | 61.52255 | 38.27857 | 4.42636087 | 290.4024 | 48.34996 | 97.24853 | 42.3244 | 313.9426 | 126.3012 |
| CEACAM7 | -1.993375465 | 0.010366 | 0.464721 | 14.652014 | 4.6732468 | 18.356866 | 78.906102 | 49.00949 | 37.29707 | 28.7713457 | 457.8109 | 1.726784 | 43.76184 | 28.63121 | 95.86034 | 160.1551 |
| AKNAD1 | -1.986832898 | 0.032136 | 0.724806 | 5.9940057 | 7.3436735 | 0 | 2.1325974 | 0 | 3.926007 | 2.21318044 | 4.270624 | 40.57943 | 9.724853 | 9.958682 | 0 | 10.41659 |
| MDGA1 | -1.97611459 | 0.012974 | 0.514478 | 88.578085 | 94.800149 | 115.9381 | 27.723766 | 19.81235 | 46.13058 | 15.4922631 | 13.666 | 15.54106 | 550.6698 | 32.36572 | 697.384 | 70.31201 |
| CDYL2 | -1.973422204 | 0.004779 | 0.312217 | 4.6620045 | 7.3436735 | 2.8984525 | 1.0662987 | 1.042755 | 4.907509 | 1.10659022 | 25.62374 | 28.49194 | 2.431213 | 6.224176 | 7.189525 | 9.11452 |
| GATA2 | -1.972823002 | 0.034316 | 0.739169 | 1.3320013 | 0.6676067 | 0 | 10.662987 | 1.042755 | 0.981502 | 2.21318044 | 11.95775 | 1.726784 | 25.52774 | 11.20352 | 1.198254 | 3.906223 |
| CIDEA | -1.972394575 | 0.023874 | 0.642071 | 0.6660006 | 16.02256 | 0 | 14.928182 | 16.68408 | 0.981502 | 3.31977065 | 13.666 | 2.590176 | 19.44971 | 14.93802 | 39.54239 | 87.23897 |
| ESR1 | -1.968587105 | 0.009099 | 0.43324 | 3.3300032 | 20.028201 | 11.59381 | 10.662987 | 10.42755 | 0 | 2.21318044 | 102.495 | 32.8089 | 6.078033 | 8.713847 | 31.15461 | 14.32282 |
| CD1D | -1.957054187 | 0.029993 | 0.699262 | 4.6620045 | 29.374694 | 2.8984525 | 3.198896 | 0 | 0.981502 | 3.31977065 | 14.52012 | 93.24634 | 3.64682 | 19.91736 | 7.189525 | 11.71867 |
| HIST1H2BC | -1.950343859 | 0.000257 | 0.057522 | 27.972027 | 32.712728 | 84.055122 | 62.911622 | 26.06888 | 20.61154 | 38.7306576 | 459.5191 | 32.8089 | 52.27108 | 205.3978 | 106.6446 | 111.9784 |
| IFI6 | -1.931215765 | 0.000196 | 0.045987 | 21.978021 | 12.01692 | 17.390715 | 18.127078 | 12.51306 | 32.38956 | 33.1977065 | 96.5161 | 25.90176 | 31.60577 | 235.2739 | 28.7581 | 63.80164 |
| GSDMA | -1.924952009 | 0.002617 | 0.231069 | 8.6580083 | 25.369054 | 3.8646033 | 14.928182 | 1.042755 | 0.981502 | 5.53295109 | 35.87324 | 52.66692 | 7.293639 | 31.12088 | 37.14588 | 35.156 |
| SLC5A1 | -1.922031202 | 3.74E-05 | 0.014329 | 117.21611 | 111.49032 | 67.630558 | 56.51383 | 45.88123 | 35.33406 | 32.0911163 | 505.6419 | 137.2793 | 87.52367 | 263.9051 | 91.06732 | 433.5907 |
| GGT1 | -1.920013773 | 2.88E-08 | 0.000105 | 142.52414 | 285.06805 | 195.16247 | 107.69617 | 138.6864 | 184.5223 | 157.135811 | 1331.581 | 718.3422 | 679.5241 | 312.4537 | 588.3428 | 298.175 |
| C10orf99 | -1.898138279 | 0.001702 | 0.18502 | 52.61405 | 13.352134 | 16.424564 | 36.254155 | 70.90735 | 82.44615 | 36.5174772 | 99.9326 | 102.7437 | 104.5422 | 52.28308 | 32.35286 | 593.7458 |
| PAPPA | -1.889375638 | 0.004715 | 0.31084 | 5.3280051 | 1.3352134 | 1.9323017 | 0 | 3.128265 | 8.833516 | 1.10659022 | 12.81187 | 12.95088 | 15.80289 | 11.20352 | 2.396508 | 14.32282 |
| SLAMF7 | -1.885154369 | 0.000271 | 0.058748 | 14.652014 | 25.369054 | 20.289167 | 13.861883 | 42.75296 | 2.944505 | 43.1570185 | 128.1187 | 115.6945 | 66.85836 | 80.91429 | 15.57731 | 106.7701 |
| NR2F1 | -1.884919346 | 0.018452 | 0.576919 | 3.9960038 | 4.6732468 | 17.390715 | 0 | 5.213776 | 27.48205 | 0 | 26.47787 | 29.35533 | 53.48669 | 53.52792 | 4.793017 | 18.22904 |
| PLA2G4E | -1.871118393 | 1.66E-06 | 0.002014 | 124.54212 | 130.1833 | 69.56286 | 152.48071 | 159.5415 | 77.53864 | 46.4767892 | 443.2908 | 380.7559 | 142.226 | 370.9609 | 220.4788 | 828.1192 |
| JAKMIP3 | -1.871085583 | 0.033366 | 0.735475 | 1.9980019 | 3.3380334 | 1.9323017 | 3.198896 | 2.08551 | 2.944505 | 0 | 7.687123 | 0 | 3.64682 | 22.40704 | 14.37905 | 1.302074 |
| ARL14 | -1.865231302 | 0.025481 | 0.661769 | 0.6660006 | 2.6704267 | 9.6615083 | 1.0662987 | 1.042755 | 0.981502 | 2.21318044 | 31.60262 | 3.453568 | 3.64682 | 2.489671 | 7.189525 | 7.812445 |
| AC011473.4 | -1.851912691 | 2.16E-05 | 0.010842 | 16.650016 | 17.357774 | 22.221469 | 17.060779 | 22.94061 | 6.870512 | 13.2790826 | 89.6831 | 69.93476 | 24.31213 | 21.1622 | 115.0324 | 39.06223 |
| ISG15 | -1.835013774 | 0.002367 | 0.218252 | 32.634031 | 83.450836 | 124.63346 | 115.16026 | 56.30878 | 86.37215 | 235.703716 | 262.2163 | 91.51956 | 59.56472 | 1336.953 | 298.3653 | 195.3111 |
| PLEKHG4B | -1.823191498 | 0.025363 | 0.661072 | 4.6620045 | 2.0028201 | 0.9661508 | 2.1325974 | 8.342041 | 3.926007 | 0 | 29.89437 | 6.043745 | 13.37167 | 9.958682 | 7.189525 | 0 |
| FOXL2 | -1.823031601 | 0.024201 | 0.643448 | 2.6640025 | 3.3380334 | 2.8984525 | 0 | 1.042755 | 0 | 0 | 5.978873 | 5.180352 | 2.431213 | 7.469012 | 3.594763 | 7.812445 |
| HIST1H4H | -1.813548113 | 0.002936 | 0.249163 | 5.9940057 | 14.01974 | 14.492262 | 33.055259 | 16.68408 | 16.68553 | 17.7054435 | 193.0322 | 6.907137 | 19.44971 | 67.22111 | 40.74064 | 27.34356 |
| IL36G | -1.803534221 | 0.036657 | 0.758646 | 87.246084 | 92.129723 | 8.6953575 | 24.52487 | 11.47031 | 2.944505 | 0 | 380.0855 | 99.29009 | 43.76184 | 85.89364 | 16.77556 | 55.98919 |
| FAM25A | -1.800173549 | 0.010329 | 0.464721 | 35.964034 | 25.369054 | 7.7292067 | 12.795584 | 13.55582 | 4.907509 | 34.3042968 | 23.06137 | 21.5848 | 58.34912 | 4.979341 | 39.54239 | 256.5086 |
| FAM25C | -1.791957661 | 0.049701 | 0.855164 | 1.9980019 | 2.0028201 | 4.8307542 | 6.3977921 | 0 | 2.944505 | 0 | 15.37425 | 11.2241 | 0 | 3.734506 | 2.396508 | 20.83319 |
| PDZK1IP1 | -1.786067209 | 0.038754 | 0.774984 | 6.6600064 | 29.374694 | 303.37136 | 212.19344 | 55.26602 | 145.2623 | 146.069909 | 1234.21 | 17.26784 | 122.7763 | 161.8286 | 599.1271 | 518.2255 |
| PLA2G4C | -1.784650442 | 0.026619 | 0.672647 | 3.9960038 | 0.6676067 | 20.289167 | 0 | 3.128265 | 0.981502 | 5.53295109 | 25.62374 | 2.590176 | 8.509246 | 19.91736 | 20.37032 | 24.73941 |
| IL31RA | -1.764053153 | 0.001831 | 0.19368 | 25.974025 | 97.470576 | 19.323017 | 15.99448 | 42.75296 | 3.926007 | 14.3856728 | 98.22435 | 216.7114 | 143.4416 | 47.30374 | 81.48129 | 55.98919 |
| CHI3L2 | -1.761152444 | 0.001588 | 0.180771 | 0.6660006 | 4.0056401 | 4.8307542 | 2.1325974 | 1.042755 | 4.907509 | 3.31977065 | 11.10362 | 10.3607 | 4.862426 | 9.958682 | 9.586034 | 14.32282 |
| GSTM3 | -1.756248424 | 0.001864 | 0.194035 | 170.49616 | 32.712728 | 26.086072 | 19.193376 | 118.8741 | 67.72362 | 46.4767892 | 221.2183 | 149.3668 | 185.9878 | 89.62814 | 80.28303 | 670.5682 |
| TREM2 | -1.750222988 | 2.93E-05 | 0.012945 | 115.88411 | 148.87629 | 203.85783 | 59.712726 | 56.30878 | 46.13058 | 149.389679 | 154.5966 | 256.4274 | 368.3288 | 309.964 | 336.7094 | 826.8171 |
| CSF2RB | -1.749019786 | 0.017287 | 0.571147 | 3.9960038 | 1.3352134 | 1.9323017 | 2.1325974 | 4.171021 | 10.79652 | 2.21318044 | 15.37425 | 2.590176 | 7.293639 | 7.469012 | 3.594763 | 40.3643 |
| TNFSF18 | -1.747407693 | 0.038415 | 0.774531 | 8.6580083 | 8.6788869 | 5.796905 | 3.198896 | 6.256531 | 0.981502 | 4.42636087 | 17.93662 | 56.98388 | 1.215607 | 4.979341 | 0 | 28.64563 |
| C8orf48 | -1.743384506 | 0.022975 | 0.633423 | 0.6660006 | 2.6704267 | 1.9323017 | 0 | 1.042755 | 3.926007 | 2.21318044 | 7.687123 | 11.2241 | 1.215607 | 6.224176 | 2.396508 | 6.510371 |
| IL36A | -1.726276249 | 0.017569 | 0.573917 | 12.654012 | 5.3408535 | 6.7630558 | 100.23208 | 34.41092 | 28.46355 | 11.0659022 | 79.4336 | 4.31696 | 158.0289 | 60.99693 | 218.0823 | 41.66637 |
| TM4SF19 | -1.692991271 | 0.006444 | 0.365317 | 3.3300032 | 12.684527 | 0.9661508 | 1.0662987 | 8.342041 | 4.907509 | 5.53295109 | 13.666 | 42.30621 | 12.15607 | 8.713847 | 17.97381 | 7.812445 |
| PSTPIP1 | -1.692026766 | 0.00525 | 0.324151 | 25.308024 | 11.349314 | 4.8307542 | 7.4640908 | 13.55582 | 4.907509 | 15.4922631 | 9.395373 | 98.4267 | 17.01849 | 60.99693 | 8.38778 | 36.45808 |
| CSF3 | -1.67826912 | 0.027656 | 0.676095 | 5.3280051 | 61.419815 | 20.289167 | 8.5303895 | 8.342041 | 10.79652 | 28.7713457 | 257.9457 | 42.30621 | 4.862426 | 43.56924 | 35.94763 | 9.11452 |
| TAGLN | -1.67774441 | 0.000809 | 0.110386 | 109.2241 | 56.078962 | 74.393614 | 52.248635 | 64.65082 | 59.87161 | 18.8120337 | 158.8672 | 135.5526 | 65.64276 | 628.6418 | 82.67954 | 124.9991 |
| TSPAN10 | -1.676727978 | 2.69E-06 | 0.002614 | 17.982017 | 23.366234 | 26.086072 | 36.254155 | 46.92398 | 27.48205 | 11.0659022 | 67.47586 | 75.9785 | 83.87685 | 156.8492 | 43.13715 | 89.84312 |
| DIRAS3 | -1.67374569 | 0.039528 | 0.777398 | 5.3280051 | 0.6676067 | 0 | 0 | 1.042755 | 3.926007 | 1.10659022 | 8.541248 | 6.907137 | 2.431213 | 6.224176 | 4.793017 | 5.208297 |
| ARMH4 | -1.668891843 | 0.01182 | 0.492049 | 8.6580083 | 7.3436735 | 4.8307542 | 14.928182 | 4.171021 | 20.61154 | 13.2790826 | 82.8501 | 3.453568 | 70.50518 | 8.713847 | 22.76683 | 11.71867 |
| TNFRSF9 | -1.65401362 | 0.048283 | 0.851768 | 3.3300032 | 2.6704267 | 0 | 4.2651947 | 2.08551 | 2.944505 | 1.10659022 | 11.95775 | 15.54106 | 4.862426 | 1.244835 | 0 | 10.41659 |
| SPOCK1 | -1.645497512 | 0.006104 | 0.353503 | 1.9980019 | 8.6788869 | 13.526112 | 5.3314934 | 0 | 4.907509 | 3.31977065 | 32.45674 | 13.81427 | 12.15607 | 17.42769 | 7.189525 | 18.22904 |
| AL031777.3 | -1.639116695 | 0.013025 | 0.514478 | 2.6640025 | 3.3380334 | 5.796905 | 4.2651947 | 1.042755 | 3.926007 | 1.10659022 | 13.666 | 2.590176 | 6.078033 | 24.89671 | 8.38778 | 3.906223 |
| OTULINL | -1.636665804 | 0.004662 | 0.30872 | 7.9920076 | 18.692987 | 21.255318 | 3.198896 | 11.47031 | 2.944505 | 6.63954131 | 83.70423 | 32.8089 | 13.37167 | 31.12088 | 10.78429 | 20.83319 |
| GJB6 | -1.63501182 | 0.000597 | 0.090543 | 1226.7732 | 530.74732 | 444.42938 | 1094.0224 | 2091.767 | 695.8847 | 544.442387 | 1619.421 | 6854.47 | 745.1668 | 1927.005 | 2053.808 | 4443.979 |
| CDKN2A | -1.633524835 | 0.007707 | 0.39537 | 101.2321 | 329.1301 | 748.76689 | 183.40337 | 82.37766 | 8.833516 | 182.587386 | 849.8542 | 396.297 | 448.5588 | 540.2585 | 748.9089 | 1368.48 |
| MAN1A1 | -1.633402083 | 0.012288 | 0.502493 | 59.940057 | 4.6732468 | 50.239843 | 6.3977921 | 13.55582 | 41.22307 | 18.8120337 | 58.93461 | 9.497313 | 127.6387 | 51.03825 | 51.52493 | 221.3526 |
| CDH16 | -1.627862502 | 0.007447 | 0.388152 | 41.95804 | 23.366234 | 26.086072 | 51.182337 | 45.88123 | 6.870512 | 36.5174772 | 20.49899 | 81.15886 | 51.05548 | 43.56924 | 38.34414 | 380.2057 |
| PKNOX2 | -1.62388303 | 0.010188 | 0.464721 | 1.3320013 | 3.3380334 | 14.492262 | 0 | 4.171021 | 2.944505 | 5.53295109 | 7.687123 | 7.770529 | 19.44971 | 12.44835 | 15.57731 | 20.83319 |
| RND1 | -1.619624578 | 0.034422 | 0.739169 | 7.9920076 | 2.6704267 | 4.8307542 | 7.4640908 | 8.342041 | 9.815017 | 3.31977065 | 77.72535 | 5.180352 | 8.509246 | 1.244835 | 19.17207 | 3.906223 |
| AEBP1 | -1.619075211 | 0.002515 | 0.228549 | 13.320013 | 4.6732468 | 8.6953575 | 3.198896 | 8.342041 | 9.815017 | 2.21318044 | 10.2495 | 26.76515 | 52.27108 | 22.40704 | 11.98254 | 10.41659 |
| SPRR1A | -1.611418376 | 0.008204 | 0.407972 | 712.62068 | 903.27185 | 342.98355 | 4031.6753 | 2480.714 | 3069.156 | 2341.5449 | 4712.206 | 880.6599 | 2045.866 | 4096.753 | 5792.361 | 18827.99 |
| SAMD15 | -1.604457093 | 0.027175 | 0.672683 | 7.326007 | 3.3380334 | 4.8307542 | 2.1325974 | 1.042755 | 0.981502 | 0 | 4.270624 | 9.497313 | 6.078033 | 22.40704 | 3.594763 | 7.812445 |

Continue on the next page…

| gene_id | log2FoldChange | pvalue | padj | nonTO-01 | nonTO-02 | nonTO-03 | nonTO-04 | nonTO-05 | nonTO-06 | nonTO-07 | TO-01 | TO-02 | TO-03 | TO-04 | TO-05 | TO-06 |
|---|---|---|---|---|---|---|---|---|---|---|---|---|---|---|---|---|
| TMC1 | -1.602100293 | 0.024949 | 0.654975 | 4.6620045 | 2.0028201 | 2.8984525 | 2.1325974 | 2.08551 | 0 | 2.21318044 | 7.687123 | 0.863392 | 8.509246 | 4.979341 | 14.37905 | 6.510371 |
| HSD17B2 | -1.601790344 | 0.030407 | 0.704335 | 0.6660006 | 2.0028201 | 12.559961 | 3.198896 | 6.256531 | 4.907509 | 0 | 30.74849 | 10.3607 | 8.509246 | 11.20352 | 11.98254 | 2.604148 |
| SLC6A11 | -1.600223799 | 9.98E-05 | 0.029663 | 107.8921 | 237.00037 | 259.89457 | 148.21552 | 143.9002 | 55.9456 | 138.323777 | 352.7535 | 873.7528 | 587.138 | 562.6656 | 121.0237 | 338.5393 |
| CNFN | -1.597930355 | 0.003322 | 0.265978 | 45.954044 | 22.698627 | 55.070597 | 151.41441 | 66.73633 | 112.8727 | 54.2229207 | 210.9688 | 57.84727 | 128.8543 | 68.46594 | 176.1434 | 677.0786 |
| KNDC1 | -1.596884712 | 0.000986 | 0.128297 | 4.6620045 | 4.6732468 | 7.7292067 | 6.3977921 | 17.72684 | 2.944505 | 9.95931196 | 9.395373 | 34.53568 | 13.37167 | 33.61055 | 27.55985 | 19.53111 |
| IGF2BP3 | -1.593133694 | 0.04464 | 0.833845 | 13.320013 | 20.695807 | 1.9323017 | 1.0662987 | 10.42755 | 15.70403 | 4.42636087 | 20.49899 | 39.71604 | 14.58728 | 85.89364 | 0 | 15.62489 |
| APOL2 | -1.590138465 | 0.00015 | 0.038454 | 111.88811 | 277.05677 | 197.09477 | 282.56915 | 163.7126 | 242.4309 | 147.176499 | 1726.186 | 375.5756 | 309.9797 | 612.459 | 361.8728 | 281.248 |
| C1QL1 | -1.5821913 | 0.019147 | 0.583617 | 7.326007 | 7.3436735 | 7.7292067 | 8.5303895 | 9.384796 | 12.75952 | 4.42636087 | 1.70825 | 5.180352 | 23.09653 | 11.20352 | 86.2743 | 20.83319 |
| DGAT2 | -1.577123101 | 1.15E-05 | 0.006719 | 93.240089 | 148.20868 | 145.88878 | 250.58019 | 204.38 | 138.3917 | 235.703716 | 653.4055 | 265.0614 | 246.7681 | 367.2264 | 479.3017 | 1096.346 |
| HIST2H2BF | -1.576705971 | 0.006205 | 0.355949 | 7.326007 | 10.681707 | 6.7630558 | 12.795584 | 6.256531 | 15.70403 | 3.31977065 | 68.32998 | 4.31696 | 15.80289 | 38.58989 | 21.56858 | 11.71867 |
| ICOSLG | -1.576069666 | 4.59E-06 | 0.003347 | 18.648018 | 17.357774 | 25.119922 | 23.458571 | 19.81235 | 58.8901 | 15.4922631 | 93.95373 | 87.2026 | 98.46413 | 70.95561 | 50.32668 | 53.38504 |
| ESPNL | -1.570512545 | 0.014663 | 0.53882 | 2.6640025 | 2.0028201 | 0 | 2.1325974 | 6.256531 | 3.926007 | 2.21318044 | 9.395373 | 14.67767 | 6.078033 | 2.489671 | 7.189525 | 7.812445 |
| XG | -1.553045757 | 3.44E-06 | 0.002946 | 122.54412 | 95.467756 | 56.036748 | 127.95584 | 105.3183 | 81.46464 | 69.7151837 | 213.5312 | 120.0115 | 393.8565 | 199.1736 | 210.8927 | 522.1318 |
| GNAO1 | -1.544776244 | 0.00792 | 0.403454 | 29.970029 | 10.0141 | 9.6615083 | 9.5966881 | 7.299286 | 5.88901 | 8.85272174 | 5.978873 | 83.74903 | 36.4682 | 44.81407 | 10.78429 | 23.43734 |
| IL1A | -1.533839512 | 0.003587 | 0.270811 | 98.568094 | 498.03459 | 127.53191 | 172.74039 | 237.7482 | 48.09358 | 126.151285 | 792.6278 | 1263.143 | 153.1664 | 387.1438 | 215.6858 | 437.4969 |
| CDRT1 | -1.524927397 | 0.018492 | 0.576919 | 3.9960038 | 12.684527 | 24.153771 | 14.928182 | 6.256531 | 16.68553 | 2.21318044 | 75.16298 | 6.043745 | 8.509246 | 65.97627 | 25.16334 | 18.22904 |
| TLR1 | -1.524877738 | 0.002238 | 0.213166 | 5.3280051 | 9.3464936 | 15.458413 | 19.193376 | 8.342041 | 9.815017 | 13.2790826 | 82.8501 | 13.81427 | 29.17456 | 31.12088 | 9.586034 | 29.94771 |
| GYG2 | -1.517728141 | 0.022567 | 0.62835 | 1.9980019 | 2.0028201 | 4.8307542 | 5.3314934 | 4.171021 | 4.907509 | 1.10659022 | 17.93662 | 8.633921 | 9.724853 | 0 | 15.57731 | 6.510371 |
| TMEM45A | -1.516665899 | 0.002989 | 0.249163 | 398.26838 | 156.88757 | 91.784329 | 79.972401 | 47.96674 | 88.33516 | 68.6085935 | 497.9547 | 233.1159 | 184.7722 | 154.3596 | 327.1234 | 893.2229 |
| PCDHAC1 | -1.515385347 | 0.039319 | 0.777398 | 0.6660006 | 3.3380334 | 8.6953575 | 5.3314934 | 2.08551 | 3.926007 | 2.21318044 | 26.47787 | 4.31696 | 3.64682 | 21.1622 | 2.396508 | 5.208297 |
| TUBB2A | -1.511192853 | 3.33E-05 | 0.013553 | 218.44821 | 180.25381 | 88.885877 | 205.79565 | 134.5154 | 148.2068 | 147.176499 | 699.5282 | 370.3952 | 193.2814 | 414.5302 | 189.3242 | 878.9001 |
| SAMD9L | -1.510704562 | 0.023664 | 0.640303 | 4.6620045 | 56.078962 | 42.510637 | 92.767985 | 58.39429 | 64.77911 | 43.1570185 | 521.8702 | 23.31159 | 31.60577 | 153.1147 | 112.6359 | 40.3643 |
| CA2 | -1.509123941 | 2.27E-06 | 0.002359 | 455.54444 | 407.90768 | 187.43326 | 412.65759 | 393.1187 | 147.2253 | 219.104863 | 1007.013 | 1480.717 | 485.027 | 812.8774 | 899.8889 | 739.5782 |
| FN1 | -1.501446105 | 0.017803 | 0.575152 | 137.19613 | 30.709908 | 83.088972 | 24.52487 | 46.92398 | 209.0599 | 38.7306576 | 398.0221 | 222.7552 | 289.3144 | 399.5921 | 13.1808 | 61.19749 |
| INA | -1.499319797 | 0.007397 | 0.388152 | 25.974025 | 35.383154 | 22.221469 | 30.922662 | 33.36816 | 23.55604 | 52.0097402 | 76.87123 | 9.497313 | 32.82138 | 105.811 | 37.14588 | 279.946 |
| GRB14 | -1.493868871 | 0.038945 | 0.774984 | 13.986013 | 2.6704267 | 8.6953575 | 5.3314934 | 2.08551 | 3.926007 | 3.31977065 | 47.83099 | 4.31696 | 6.078033 | 27.38638 | 2.396508 | 9.11452 |
| PGBD5 | -1.480293423 | 0.004189 | 0.287825 | 11.988011 | 58.081782 | 195.16247 | 60.779025 | 128.2589 | 103.0577 | 170.414894 | 479.164 | 174.4052 | 149.5196 | 474.2822 | 98.25685 | 361.9766 |
| POU3F1 | -1.47872116 | 0.001996 | 0.200767 | 109.2241 | 42.059221 | 20.289167 | 61.845324 | 65.69357 | 32.38956 | 17.7054435 | 23.91549 | 206.3507 | 162.8913 | 148.1354 | 179.7381 | 115.8846 |
| PTHLH | -1.47609795 | 0.00115 | 0.145717 | 2909.0908 | 3085.0105 | 1208.6547 | 980.99479 | 1007.301 | 497.6214 | 821.089942 | 8126.997 | 2692.057 | 2352.199 | 6765.68 | 2316.225 | 2812.48 |
| KRT14 | -1.473398849 | 0.003575 | 0.270811 | 231140.85 | 89955.328 | 49090.124 | 45168.412 | 64183.66 | 15812.97 | 86190.0989 | 90409.11 | 305919.7 | 215824.9 | 186073 | 104426.7 | 481465.4 |
| LTBP1 | -1.467160987 | 0.003469 | 0.270811 | 1768.8977 | 2842.0017 | 656.01641 | 889.2931 | 723.6721 | 207.0969 | 650.675048 | 2896.337 | 7207.597 | 2201.464 | 2423.694 | 1302.502 | 2308.578 |
| CYYR1 | -1.464944341 | 0.005144 | 0.322914 | 107.8921 | 66.093062 | 15.458413 | 25.591168 | 20.8551 | 54.9641 | 12.1724924 | 85.41248 | 261.6078 | 168.9693 | 57.26242 | 74.29176 | 72.91616 |
| AFAP1L2 | -1.455169905 | 4.48E-05 | 0.015828 | 273.06026 | 84.786049 | 137.19342 | 115.16026 | 56.30878 | 137.4102 | 73.0349544 | 481.7264 | 439.4666 | 203.0063 | 211.622 | 387.0361 | 341.1434 |
| ATG9B | -1.452223317 | 0.027194 | 0.672683 | 149.18414 | 30.042301 | 96.615083 | 115.16026 | 46.92398 | 81.46464 | 18.8120337 | 380.0855 | 25.03837 | 326.9982 | 90.87298 | 424.182 | 15.62489 |
| ITGBL1 | -1.444690446 | 0.044406 | 0.83162 | 9.3240089 | 32.045121 | 38.646033 | 13.861883 | 7.299286 | 1.963003 | 37.6240674 | 67.47586 | 44.89639 | 32.82138 | 1.244835 | 32.35286 | 149.7385 |
| HIST1H2BD | -1.419036508 | 0.002321 | 0.215395 | 61.272059 | 48.735288 | 134.29497 | 99.165777 | 86.54868 | 110.9097 | 128.364465 | 730.2767 | 67.34458 | 93.60171 | 192.9495 | 210.8927 | 235.6754 |
| SLIT3 | -1.418973022 | 0.047755 | 0.848751 | 632.0346 | 16.02256 | 6.7630558 | 1.0662987 | 6.256531 | 47.11208 | 1.10659022 | 22.20724 | 33.67229 | 109.4046 | 24.89671 | 21.56858 | 19.53111 |
| KLF13 | -1.41318246 | 0.045428 | 0.836779 | 86.580083 | 48.067681 | 0.9661508 | 217.52493 | 176.2256 | 44.16758 | 113.978792 | 57.22636 | 404.9309 | 564.0415 | 296.2708 | 46.73192 | 199.2174 |
| SERPING1 | -1.403831881 | 0.034211 | 0.739169 | 17.316017 | 18.025381 | 0.9661508 | 49.049739 | 53.18051 | 49.07509 | 14.3856728 | 251.1127 | 25.90176 | 48.62426 | 28.63121 | 56.31795 | 45.5726 |
| VGLL3 | -1.401942093 | 0.02342 | 0.635429 | 1.3320013 | 4.6732468 | 18.356866 | 14.928182 | 9.384796 | 18.64853 | 11.0659022 | 34.16499 | 12.95088 | 14.58728 | 87.13847 | 16.77556 | 10.41659 |
| S100A9 | -1.400220275 | 0.018319 | 0.576919 | 4274.3921 | 11650.404 | 20281.438 | 31734.115 | 19036.54 | 15356.58 | 34492.4171 | 74233.69 | 5228.702 | 13967.32 | 21728.6 | 50259.58 | 144130.5 |
| BRSK2 | -1.398521304 | 0.019043 | 0.582904 | 5.3280051 | 2.0028201 | 7.7292067 | 4.2651947 | 2.08551 | 3.926007 | 0 | 9.395373 | 4.31696 | 7.293639 | 17.42769 | 11.98254 | 7.812445 |
| IL36RN | -1.397438318 | 0.010927 | 0.480325 | 113.88611 | 66.093062 | 14.492262 | 75.707206 | 94.89072 | 67.72362 | 135.004007 | 193.0322 | 155.4106 | 109.4046 | 93.36265 | 49.12842 | 682.2869 |
| CLDND2 | -1.395895161 | 0.022037 | 0.620724 | 3.9960038 | 4.6732468 | 3.8646033 | 3.198896 | 1.042755 | 2.944505 | 1.10659022 | 11.10362 | 1.726784 | 10.94046 | 4.979341 | 10.78429 | 9.11452 |
| GPLD1 | -1.395053297 | 0.017685 | 0.573917 | 4.6620045 | 5.3408535 | 10.627659 | 3.198896 | 13.55582 | 18.64853 | 7.74613153 | 14.52012 | 4.31696 | 31.60577 | 8.713847 | 29.95636 | 54.68712 |
| LRRC6 | -1.389501318 | 0.009786 | 0.450441 | 5.9940057 | 9.3464936 | 10.627659 | 6.3977921 | 17.72684 | 36.31556 | 7.74613153 | 80.28773 | 9.497313 | 25.52774 | 24.89671 | 37.14588 | 32.55186 |
| TMPRSS11F | -1.376860175 | 0.008149 | 0.407972 | 5.9940057 | 37.385974 | 10.627659 | 20.259675 | 25.02612 | 9.815017 | 42.0504283 | 92.24548 | 33.67229 | 19.44971 | 56.01759 | 26.36159 | 108.0722 |
| SUGCT | -1.370827532 | 0.004583 | 0.304932 | 310.3563 | 424.59785 | 173.90715 | 74.640908 | 87.59143 | 40.24157 | 221.318044 | 322.8592 | 701.9378 | 318.4889 | 873.8744 | 239.6508 | 501.2986 |
| BMP6 | -1.369294516 | 0.017157 | 0.571147 | 3.9960038 | 12.684527 | 7.7292067 | 8.5303895 | 6.256531 | 3.926007 | 2.21318044 | 17.0825 | 11.2241 | 12.15607 | 4.979341 | 9.586034 | 46.87467 |
| LHX5 | -1.365800918 | 0.001786 | 0.192717 | 11.322011 | 16.02256 | 9.6615083 | 9.5966881 | 6.256531 | 7.852014 | 4.42636087 | 44.41449 | 26.76515 | 17.01849 | 8.713847 | 29.95636 | 18.22904 |
| RHBG | -1.344442228 | 0.029346 | 0.695232 | 3.3300032 | 8.0112802 | 22.221469 | 47.983441 | 15.64133 | 16.68553 | 16.5988533 | 40.14386 | 5.180352 | 25.52774 | 92.11781 | 92.26558 | 27.34356 |
| WFDC5 | -1.334042527 | 0.003013 | 0.249163 | 48.618047 | 18.025381 | 2.8984525 | 24.52487 | 32.32541 | 20.61154 | 14.3856728 | 72.60061 | 59.57405 | 63.21154 | 23.65187 | 62.30922 | 69.00993 |
| COL1A1 | -1.333025479 | 0.010545 | 0.471311 | 468.19845 | 114.16074 | 229.9439 | 85.303895 | 172.0546 | 390.6377 | 39.8372478 | 297.2354 | 713.1619 | 1149.964 | 590.0519 | 283.9863 | 207.0298 |
| KRT9 | -1.329958823 | 0.005082 | 0.322914 | 21.31202 | 11.349314 | 3.8646033 | 4.2651947 | 7.299286 | 5.88901 | 5.53295109 | 15.37425 | 35.39908 | 21.88092 | 14.93802 | 15.57731 | 28.64563 |
| CERS3 | -1.32826615 | 0.001603 | 0.181068 | 362.30435 | 128.84809 | 243.47001 | 656.83999 | 825.8621 | 291.506 | 320.911163 | 840.4588 | 996.3545 | 1049.068 | 324.902 | 1008.93 | 1868.477 |
| WNT6 | -1.327890002 | 0.043323 | 0.820575 | 43.290041 | 16.02256 | 24.153771 | 52.248635 | 9.384796 | 37.29707 | 15.4922631 | 5.124749 | 13.81427 | 192.0658 | 22.40704 | 95.86034 | 97.65557 |
| PCDHB15 | -1.325440673 | 0.027932 | 0.678993 | 11.322011 | 4.6732468 | 3.8646033 | 2.1325974 | 2.08551 | 1.963003 | 1.10659022 | 17.0825 | 11.2241 | 6.078033 | 12.44835 | 7.189525 | 6.510371 |
| HIST1H2BE | -1.319417442 | 0.005285 | 0.324898 | 16.650016 | 16.02256 | 21.255318 | 30.922662 | 10.42755 | 22.57454 | 12.1724924 | 120.4316 | 17.26784 | 25.52774 | 39.83473 | 53.92144 | 19.53111 |
| DDX60 | -1.317930757 | 0.003384 | 0.267024 | 93.90609 | 160.89321 | 122.70116 | 73.574609 | 139.7292 | 160.9663 | 139.430367 | 338.2334 | 193.3998 | 127.6387 | 917.4436 | 239.6508 | 88.54105 |
| MAB21L3 | -1.317383688 | 0.008146 | 0.407972 | 11.988011 | 26.704267 | 52.172145 | 76.773505 | 105.3183 | 78.52014 | 27.6647555 | 147.7636 | 37.98925 | 69.28958 | 109.5455 | 200.1085 | 243.4879 |
| CEACAM1 | -1.310081765 | 0.033077 | 0.734659 | 177.15617 | 1040.7988 | 1298.5067 | 1116.4147 | 945.7789 | 672.3287 | 936.175324 | 7613.668 | 206.3507 | 470.4397 | 1358.115 | 2336.596 | 1162.752 |
| DNER | -1.302290129 | 0.047562 | 0.84717 | 207.1262 | 102.14382 | 37.679882 | 22.392272 | 39.6247 | 3.926007 | 90.7403979 | 136.66 | 273.6953 | 137.3635 | 197.9288 | 17.97381 | 303.3833 |
| PIK3IP1 | -1.291226151 | 0.003148 | 0.25624 | 255.07824 | 276.38917 | 266.65763 | 371.07194 | 247.133 | 422.0457 | 192.546698 | 2094.314 | 223.6185 | 461.9305 | 429.4682 | 726.1421 | 322.9144 |
| NRG4 | -1.290550029 | 0.02453 | 0.649834 | 10.65601 | 13.352134 | 13.526112 | 5.3314934 | 5.213776 | 3.926007 | 3.31977065 | 37.58149 | 31.08211 | 20.66531 | 16.18286 | 8.38778 | 2.604148 |
| MYL9 | -1.283780775 | 0.003872 | 0.27518 | 28.638027 | 44.062041 | 49.273692 | 42.651947 | 38.58194 | 29.44505 | 24.3449848 | 152.8883 | 22.44819 | 30.39016 | 184.2356 | 81.48129 | 65.10371 |
| UCP2 | -1.280097375 | 0.008243 | 0.408485 | 55.278053 | 60.752208 | 279.21759 | 100.23208 | 71.95011 | 61.83461 | 175.947845 | 600.4497 | 108.7874 | 91.17049 | 319.9227 | 330.7182 | 222.6547 |
| KIF7 | -1.279204899 | 0.034202 | 0.739169 | 51.94805 | 82.783229 | 117.8704 | 58.646427 | 22.94061 | 23.55604 | 39.8372478 | 101.6408 | 53.53031 | 57.13351 | 502.9135 | 91.06732 | 22.13526 |
| OGDHL | -1.274247549 | 0.022598 | 0.62835 | 5.9940057 | 13.352134 | 9.6615083 | 5.3314934 | 2.08551 | 14.72253 | 11.0659022 | 5.124749 | 41.44282 | 13.37167 | 9.958682 | 19.17207 | 40.3643 |
| HIST1H3E | -1.270779 | 0.002032 | 0.200767 | 29.304028 | 30.709908 | 19.323017 | 26.657467 | 30.2399 | 34.35256 | 8.85272174 | 132.3893 | 25.90176 | 37.6838 | 64.73144 | 74.29176 | 36.45808 |
| B4GALNT3 | -1.269807362 | 3.56E-07 | 0.000742 | 376.29036 | 426.60067 | 492.73692 | 397.72941 | 469.2398 | 223.7824 | 159.348991 | 909.6429 | 834.0368 | 739.0888 | 1000.848 | 956.2069 | 824.213 |
| KLK14 | -1.267289455 | 0.013 | 0.514478 | 8.6580083 | 8.6788869 | 7.7292067 | 6.3977921 | 7.299286 | 15.70403 | 4.42636087 | 6.832998 | 23.31159 | 15.80289 | 6.224176 | 45.53366 | 24.73941 |
| ANK1 | -1.267220431 | 0.016401 | 0.568222 | 213.7862 | 738.37299 | 349.7466 | 405.1935 | 178.3111 | 264.024 | 105.126071 | 182.7827 | 349.6738 | 1112.28 | 1549.82 | 1246.184 | 213.5402 |
| CWH43 | -1.263292525 | 0.003779 | 0.274565 | 104.5621 | 132.18612 | 145.88878 | 324.1548 | 389.9904 | 179.6148 | 101.8063 | 702.9447 | 190.8096 | 292.9612 | 328.6365 | 289.9775 | 1028.639 |
| RAB6B | -1.262714725 | 0.010671 | 0.474016 | 41.95804 | 11.349314 | 41.544486 | 21.325974 | 15.64133 | 46.13058 | 17.7054435 | 149.4718 | 19.85802 | 27.95895 | 100.8317 | 61.11097 | 42.96845 |
| HIST4H4 | -1.26092033 | 0.033336 | 0.735475 | 2.6640025 | 9.3464936 | 15.458413 | 15.99448 | 15.64133 | 4.907509 | 4.42636087 | 60.64286 | 12.95088 | 8.509246 | 24.89671 | 25.16334 | 6.510371 |
| XAF1 | -1.25785238 | 0.003044 | 0.249163 | 15.318015 | 22.698627 | 60.867502 | 31.98896 | 36.49643 | 66.74212 | 22.1318044 | 156.3048 | 25.90176 | 72.93639 | 75.93495 | 94.66208 | 97.65557 |
| IVL | -1.254028877 | 0.024115 | 0.643448 | 170.49616 | 970.03251 | 805.76979 | 1427.7739 | 1928.054 | 608.5311 | 939.495095 | 5511.667 | 969.5893 | 769.479 | 3562.719 | 422.9837 | 2765.606 |
| APOE | -1.25058469 | 0.017004 | 0.569971 | 229.10422 | 96.802969 | 257.96227 | 404.1272 | 727.8431 | 581.049 | 65.2888229 | 1433.221 | 320.3185 | 747.598 | 248.9671 | 1119.169 | 946.608 |
| TCHH | -1.249525569 | 0.001651 | 0.18358 | 7.326007 | 20.028201 | 24.153771 | 7.4640908 | 8.342041 | 14.72253 | 16.5988533 | 37.58149 | 37.98925 | 15.80289 | 54.77275 | 29.95636 | 24.73941 |
| PARVB | -1.244344422 | 0.001822 | 0.19368 | 42.624041 | 76.107162 | 90.818178 | 94.900583 | 81.3349 | 37.29707 | 115.085383 | 117.8692 | 370.3952 | 82.66125 | 113.28 | 107.8429 | 298.175 |
| TMEM191B | -1.239822871 | 0.045507 | 0.837034 | 3.9960038 | 6.6760669 | 32.849128 | 7.4640908 | 10.42755 | 7.852014 | 15.4922631 | 32.45674 | 3.453568 | 27.95895 | 22.40704 | 11.98254 | 72.91616 |
| SBSN | -1.200601546 | 0.010945 | 0.480325 | 2701.9646 | 1377.9402 | 839.58507 | 2108.0725 | 2410.85 | 1288.712 | 1686.44349 | 1756.935 | 2107.54 | 3586.039 | 1160.186 | 3307.182 | 12538.97 |
| ADAM21 | -1.199726799 | 0.0265 | 0.672647 | 1.9980019 | 9.3464936 | 4.8307542 | 4.2651947 | 1.042755 | 4.907509 | 4.42636087 | 12.81187 | 12.08749 | 7.293639 | 8.713847 | 4.793017 | 15.62489 |
| C1QTNF1 | -1.193840848 | 0.047274 | 0.844469 | 172.49417 | 78.109982 | 18.356866 | 14.928182 | 14.59857 | 16.68553 | 34.3042968 | 118.7233 | 75.9785 | 108.189 | 104.5662 | 37.14588 | 244.79 |
| RUNDC3A | -1.192314207 | 0.003839 | 0.27518 | 11.322011 | 47.400075 | 50.239843 | 19.193376 | 22.94061 | 26.50055 | 40.9438381 | 128.1187 | 53.53031 | 29.17456 | 57.26242 | 56.31795 | 102.8639 |
| SNPH | -1.191709383 | 0.000282 | 0.058748 | 33.966033 | 59.416995 | 70.529011 | 76.773505 | 51.095 | 54.9641 | 59.7558718 | 208.4064 | 66.48119 | 87.52367 | 221.5807 | 131.808 | 78.12445 |
| HIST1H2AC | -1.190312143 | 0.001691 | 0.18502 | 183.15018 | 212.29893 | 239.60541 | 324.1548 | 249.2185 | 314.0806 | 220.211453 | 1381.974 | 172.6784 | 301.4704 | 510.3825 | 426.5785 | 613.277 |
| CD83 | -1.186749903 | 0.023687 | 0.640303 | 35.964034 | 22.698627 | 4.8307542 | 10.662987 | 14.59857 | 14.72253 | 29.8779359 | 81.99598 | 53.53031 | 9.724853 | 31.12088 | 17.97381 | 66.40579 |
| AOX1 | -1.180871991 | 0.016453 | 0.568222 | 71.262068 | 386.54427 | 109.17504 | 89.569089 | 83.42041 | 38.27857 | 101.8063 | 583.3672 | 267.6515 | 234.6121 | 347.309 | 98.25685 | 180.9883 |
| KIAA1549L | -1.179259489 | 0.003351 | 0.266808 | 18.648018 | 86.788869 | 38.646033 | 55.447531 | 42.75296 | 25.51904 | 59.7558718 | 139.2223 | 64.75441 | 42.54623 | 182.9908 | 124.6184 | 82.03068 |
| DDX58 | -1.174784966 | 0.000689 | 0.100899 | 71.928069 | 107.48468 | 85.021273 | 84.237596 | 88.63419 | 70.66812 | 71.9283642 | 267.3411 | 92.38295 | 75.36761 | 380.9196 | 137.7992 | 169.2696 |
| ADAP2 | -1.171655468 | 0.000339 | 0.065591 | 42.624041 | 54.076142 | 41.544486 | 42.651947 | 84.46317 | 34.35256 | 58.6492816 | 110.1821 | 78.56868 | 75.36761 | 68.46594 | 131.808 | 227.863 |
| RGL1 | -1.169653441 | 0.010335 | 0.464721 | 20.64602 | 20.028201 | 5.796905 | 19.193376 | 19.81235 | 43.18608 | 8.85272174 | 28.18612 | 77.70529 | 25.52774 | 48.54858 | 21.56858 | 63.80164 |
| SH2D5 | -1.165821603 | 0.038119 | 0.773457 | 526.1405 | 520.06561 | 114.97195 | 76.773505 | 149.114 | 56.9271 | 227.957585 | 284.4236 | 1133.634 | 295.3924 | 924.9126 | 146.187 | 433.5907 |
| LRRC3 | -1.156652691 | 0.015552 | 0.554978 | 113.88611 | 22.031021 | 18.356866 | 36.254155 | 36.49643 | 12.75952 | 15.4922631 | 96.5161 | 108.7874 | 83.87685 | 97.09715 | 44.33541 | 59.89541 |
| YPEL2 | -1.155575956 | 0.021514 | 0.620275 | 79.254076 | 46.064861 | 121.735 | 156.74591 | 87.59143 | 126.6137 | 35.410887 | 562.0141 | 71.66154 | 99.67974 | 212.8668 | 240.8491 | 58.59334 |
| HIST1H2BJ | -1.154905808 | 0.006943 | 0.380273 | 18.648018 | 14.687347 | 11.59381 | 20.259675 | 8.342041 | 5.88901 | 19.9186239 | 54.66399 | 18.99463 | 18.2341 | 53.52792 | 16.77556 | 27.34356 |
| CARD16 | -1.154514981 | 0.003302 | 0.265799 | 37.296036 | 15.354954 | 25.119922 | 22.392272 | 28.15439 | 16.68553 | 25.451575 | 95.66198 | 41.44282 | 36.4682 | 42.3244 | 17.97381 | 91.1452 |
| PPP2R2C | -1.154135504 | 0.000175 | 0.042564 | 115.21811 | 133.52134 | 196.12862 | 182.33707 | 252.3467 | 104.0392 | 100.69971 | 515.0372 | 151.0936 | 438.834 | 288.8018 | 378.6483 | 294.2688 |
| HIST1H2BK | -1.151681543 | 0.006782 | 0.372895 | 91.908088 | 138.86219 | 176.8056 | 182.33707 | 87.59143 | 105.0207 | 220.211453 | 755.0463 | 106.1972 | 111.8358 | 239.0084 | 285.1845 | 410.1534 |
| IFIT3 | -1.141187646 | 0.015659 | 0.556478 | 46.620045 | 96.802969 | 55.070597 | 51.182337 | 37.53919 | 69.68662 | 70.821774 | 122.1398 | 62.16423 | 66.85836 | 424.4888 | 87.47256 | 46.87467 |
| DPF1 | -1.128945939 | 0.011589 | 0.4902 | 17.316017 | 20.695807 | 6.7630558 | 10.662987 | 12.51306 | 3.926007 | 21.0252141 | 11.10362 | 42.30621 | 19.44971 | 37.34506 | 23.96508 | 41.66637 |
| TTC7B | -1.126420726 | 0.000788 | 0.110386 | 111.88811 | 146.87347 | 47.341391 | 71.442012 | 61.52255 | 33.37106 | 76.354725 | 117.0151 | 278.8756 | 160.4601 | 179.2563 | 154.5748 | 140.624 |
| GDPD2 | -1.125995542 | 0.000852 | 0.112885 | 51.94805 | 58.749388 | 45.409089 | 37.320454 | 33.36816 | 16.68553 | 74.1415446 | 124.7022 | 159.7275 | 64.42715 | 75.93495 | 68.30049 | 100.2597 |

Continue on the next page…

| gene_id | log2FoldChange | pvalue | padj | nonTO-01 | nonTO-02 | nonTO-03 | nonTO-04 | nonTO-05 | nonTO-06 | nonTO-07 | TO-01 | TO-02 | TO-03 | TO-04 | TO-05 | TO-06 |
|---|---|---|---|---|---|---|---|---|---|---|---|---|---|---|---|---|
| IQCA1 | -1.125073355 | 3.45E-05 | 0.013598 | 33.300032 | 50.070501 | 26.086072 | 44.784545 | 44.83847 | 55.9456 | 50.90315 | 132.3893 | 93.24634 | 53.48669 | 125.7284 | 97.05859 | 67.70786 |
| SLC39A2 | -1.12394348 | 0.027805 | 0.678599 | 40.626039 | 25.369054 | 48.307542 | 69.309414 | 81.3349 | 67.72362 | 136.110597 | 245.1338 | 29.35533 | 87.52367 | 56.01759 | 118.6272 | 337.2372 |
| MARCKSL1 | -1.114131756 | 0.018945 | 0.58233 | 304.36229 | 388.54709 | 636.6934 | 213.25974 | 134.5154 | 215.9304 | 252.30257 | 1883.345 | 204.6239 | 260.1398 | 520.3412 | 567.9725 | 544.267 |
| BCAT1 | -1.112018569 | 0.012312 | 0.502493 | 191.80818 | 552.11073 | 351.6789 | 942.60803 | 507.8218 | 226.7269 | 193.653288 | 1495.572 | 1625.767 | 407.2282 | 368.4712 | 721.349 | 874.9939 |
| TMPRSS13 | -1.10963506 | 0.027961 | 0.678993 | 49.284047 | 88.124083 | 79.224368 | 103.43097 | 462.9833 | 267.95 | 217.998273 | 524.4326 | 126.0552 | 265.0022 | 323.6572 | 321.1321 | 785.1508 |
| CTSV | -1.104062602 | 0.000186 | 0.044509 | 768.56474 | 459.3134 | 256.99612 | 380.66863 | 314.9121 | 270.8945 | 712.6441 | 922.4548 | 910.8786 | 971.2697 | 1004.582 | 644.6608 | 1378.897 |
| CTXN1 | -1.103144405 | 0.011341 | 0.4902 | 25.308024 | 30.709908 | 25.119922 | 24.52487 | 9.384796 | 8.833516 | 28.7713457 | 78.57948 | 22.44819 | 34.03698 | 89.62814 | 31.15461 | 26.04148 |
| RIMS3 | -1.103136472 | 0.004882 | 0.31472 | 169.16416 | 73.436735 | 70.529011 | 44.784545 | 92.80521 | 49.07509 | 28.7713457 | 183.6368 | 265.9248 | 178.6942 | 149.3802 | 73.09351 | 124.9991 |
| ADTRP | -1.100463624 | 0.01303 | 0.514478 | 91.908088 | 62.087422 | 24.153771 | 37.320454 | 67.77908 | 22.57454 | 32.0911163 | 43.56036 | 115.6945 | 55.9179 | 77.17979 | 89.86907 | 242.1858 |
| HIST1H4I | -1.099908822 | 0.009624 | 0.446564 | 32.634031 | 19.360594 | 48.307542 | 47.983441 | 42.75296 | 31.40806 | 45.3701989 | 180.2203 | 21.5848 | 35.25259 | 92.11781 | 69.49875 | 91.1452 |
| CNNM1 | -1.095924162 | 0.011594 | 0.4902 | 41.95804 | 52.740928 | 28.018374 | 44.784545 | 42.75296 | 41.22307 | 24.3449848 | 27.33199 | 50.07674 | 65.64276 | 62.24176 | 239.6508 | 62.49956 |
| TLR6 | -1.094901725 | 0.01993 | 0.595967 | 38.628037 | 19.360594 | 33.815279 | 29.856363 | 5.213776 | 11.77802 | 13.2790826 | 51.24749 | 43.1696 | 59.56472 | 75.93495 | 11.98254 | 37.76015 |
| ANGPTL4 | -1.094210936 | 0.013508 | 0.524838 | 713.95268 | 668.9419 | 311.10057 | 366.80675 | 235.6627 | 237.5234 | 317.591393 | 2460.733 | 383.3461 | 410.875 | 754.3702 | 653.0486 | 557.2878 |
| TMEM204 | -1.093519331 | 0.046672 | 0.840239 | 15.318015 | 14.687347 | 38.646033 | 6.3977921 | 12.51306 | 14.72253 | 3.31977065 | 26.47787 | 40.57943 | 9.724853 | 28.63121 | 14.37905 | 74.21823 |
| EHD3 | -1.093094937 | 0.018462 | 0.576919 | 100.5661 | 140.1974 | 349.7466 | 412.65759 | 330.5534 | 199.2449 | 327.550705 | 1258.98 | 88.06599 | 390.2097 | 490.4651 | 680.6084 | 490.882 |
| ADAMTS17 | -1.092506739 | 0.026592 | 0.672647 | 689.31066 | 9.3464936 | 6.7630558 | 36.254155 | 9.384796 | 33.37106 | 6.63954131 | 35.01912 | 52.66692 | 40.11502 | 13.69319 | 39.54239 | 58.59334 |
| ZNF883 | -1.092071009 | 0.000536 | 0.085199 | 33.300032 | 24.701447 | 39.612184 | 63.977921 | 37.53919 | 17.66703 | 47.5833794 | 115.3068 | 70.79815 | 49.83987 | 59.75209 | 103.0499 | 80.7286 |
| HIST1H1C | -1.081732191 | 0.017566 | 0.573917 | 121.21212 | 127.51288 | 365.20501 | 317.75701 | 214.8076 | 281.691 | 374.027494 | 1211.149 | 91.51956 | 294.1768 | 438.182 | 674.6171 | 557.2878 |
| DACT2 | -1.075695897 | 0.036505 | 0.757515 | 58.608056 | 22.698627 | 3.8646033 | 23.458571 | 19.81235 | 50.05659 | 21.0252141 | 13.666 | 44.89639 | 96.03292 | 53.52792 | 77.88653 | 76.82238 |
| BIRC3 | -1.066378479 | 0.011638 | 0.4902 | 70.596068 | 185.59466 | 54.104447 | 123.69065 | 44.83847 | 40.24157 | 89.6338077 | 399.7304 | 119.1481 | 151.9508 | 210.3772 | 107.8429 | 104.1659 |
| MICAL1 | -1.065817418 | 0.011771 | 0.491417 | 339.66033 | 405.23726 | 107.24274 | 97.03318 | 165.7981 | 135.4472 | 203.6126 | 537.2445 | 717.4788 | 226.1028 | 539.0137 | 129.4115 | 462.2363 |
| SPRR1B | -1.065610176 | 0.002039 | 0.200767 | 3912.0877 | 4651.2158 | 3334.1865 | 7443.8311 | 6103.246 | 6357.187 | 7513.74758 | 18587.46 | 4355.813 | 6226.337 | 9908.889 | 11398.99 | 20054.55 |
| CCDC71L | -1.064633141 | 0.002197 | 0.210629 | 282.38427 | 232.99473 | 116.90425 | 129.02214 | 112.6176 | 258.135 | 147.176499 | 569.7012 | 539.6201 | 238.2589 | 245.2326 | 176.1434 | 526.038 |
| TMEM86A | -1.059306042 | 0.039396 | 0.777398 | 9.9900096 | 71.433915 | 107.24274 | 37.320454 | 68.82184 | 30.42655 | 46.4767892 | 232.3219 | 34.53568 | 41.33062 | 113.28 | 80.28303 | 161.4572 |
| CDA | -1.058584164 | 0.021899 | 0.620275 | 63.936061 | 63.422635 | 79.224368 | 60.779025 | 86.54868 | 53.00109 | 252.30257 | 477.4558 | 76.8419 | 165.3225 | 118.2594 | 118.6272 | 217.4464 |
| CSTA | -1.051092981 | 0.037418 | 0.765694 | 3330.0032 | 3273.2756 | 4711.9176 | 9313.0527 | 8573.533 | 4657.226 | 10787.0414 | 10486.09 | 4868.668 | 5814.246 | 5543.252 | 8469.261 | 44112.97 |
| GPRC5D | -1.045458458 | 0.019305 | 0.585996 | 5.3280051 | 15.354954 | 27.052223 | 19.193376 | 18.76959 | 12.75952 | 6.63954131 | 33.31087 | 19.85802 | 9.724853 | 44.81407 | 33.55112 | 44.27052 |
| SLC31A2 | -1.044724914 | 2.92E-06 | 0.002659 | 398.26838 | 472.66553 | 282.11604 | 358.27636 | 333.6816 | 188.4483 | 382.880215 | 835.334 | 768.419 | 536.0825 | 687.1491 | 536.8179 | 911.452 |
| HERC6 | -1.042137962 | 0.043366 | 0.820575 | 51.282049 | 62.087422 | 108.20889 | 133.28734 | 83.42041 | 106.0022 | 172.628074 | 520.162 | 74.25172 | 58.34912 | 395.8576 | 155.7731 | 58.59334 |
| EPGN | -1.029536982 | 0.020386 | 0.59784 | 76.590073 | 38.721188 | 112.0735 | 27.723766 | 94.89072 | 61.83461 | 52.0097402 | 140.0765 | 144.1865 | 55.9179 | 73.44528 | 63.50747 | 334.6331 |
| TH | -1.018814744 | 0.03407 | 0.739169 | 27.306026 | 15.354954 | 4.8307542 | 11.729285 | 10.42755 | 5.88901 | 19.9186239 | 16.22837 | 51.80352 | 37.6838 | 32.36572 | 14.37905 | 14.32282 |
| BBS12 | -1.018369786 | 0.005879 | 0.346763 | 31.968031 | 40.056401 | 31.882977 | 26.657467 | 23.98337 | 15.70403 | 40.9438381 | 107.6197 | 101.8803 | 38.89941 | 43.56924 | 29.95636 | 42.96845 |
| CCND2 | -1.013956743 | 0.014365 | 0.534801 | 650.68262 | 394.55555 | 1027.0183 | 371.07194 | 376.4346 | 332.7291 | 286.606866 | 1751.81 | 974.7697 | 391.4253 | 1791.318 | 520.0423 | 523.4338 |
| JAZF1 | -1.011731762 | 0.008104 | 0.407972 | 27.306026 | 28.707087 | 35.747581 | 20.259675 | 31.28265 | 18.64853 | 26.5581652 | 117.8692 | 50.94013 | 32.82138 | 62.24176 | 41.9389 | 18.22904 |
| ELOVL7 | -1.011308917 | 0.00852 | 0.419397 | 53.280051 | 155.55236 | 76.325916 | 45.850843 | 71.95011 | 44.16758 | 85.2074468 | 327.9839 | 116.5579 | 110.6202 | 179.2563 | 80.28303 | 105.468 |
| IFIT2 | -1.01024946 | 0.018921 | 0.58233 | 43.956042 | 72.101522 | 47.341391 | 81.0387 | 53.18051 | 73.61263 | 78.5679055 | 82.8501 | 53.53031 | 74.152 | 375.9403 | 128.2132 | 62.49956 |
| HIST2H2BE | -1.007712001 | 0.018343 | 0.576919 | 71.928069 | 76.774769 | 115.9381 | 149.28182 | 117.8313 | 130.5397 | 57.5426913 | 476.6016 | 48.34996 | 134.9323 | 275.1086 | 190.5224 | 113.2805 |
| P3H3 | 1.004902552 | 0.030907 | 0.710858 | 388.27837 | 64.757849 | 113.03965 | 340.14928 | 178.3111 | 318.9881 | 326.444114 | 93.95373 | 23.31159 | 212.7312 | 102.0765 | 159.3678 | 148.4365 |
| RHOBTB3 | 1.016355674 | 0.007031 | 0.38366 | 801.86477 | 1008.7537 | 2160.3133 | 1097.2213 | 1172.057 | 2232.916 | 723.710003 | 1048.011 | 290.9631 | 822.9657 | 820.3465 | 659.0398 | 255.2065 |
| CNTNAP3B | 1.029926868 | 0.016788 | 0.569971 | 109.89011 | 190.26791 | 173.90715 | 58.646427 | 60.4798 | 35.33406 | 125.044695 | 82.8501 | 67.34458 | 35.25259 | 28.63121 | 76.68827 | 24.73941 |
| RBMS3 | 1.032550264 | 0.012944 | 0.514478 | 175.15817 | 92.797329 | 33.815279 | 60.779025 | 72.99286 | 96.18717 | 38.7306576 | 51.24749 | 39.71604 | 53.48669 | 14.93802 | 55.11969 | 24.73941 |
| SLC16A14 | 1.042596248 | 0.0087 | 0.424122 | 124.54212 | 107.48468 | 144.92262 | 189.80117 | 198.1235 | 230.6529 | 293.246408 | 202.4276 | 28.49194 | 81.44564 | 43.56924 | 100.6534 | 78.12445 |
| PROCA1 | 1.058692435 | 0.023843 | 0.642071 | 61.938059 | 30.709908 | 22.221469 | 23.458571 | 29.19714 | 75.57563 | 21.0252141 | 12.81187 | 18.99463 | 44.97744 | 12.44835 | 10.78429 | 9.11452 |
| ARHGEF35 | 1.062979416 | 0.016854 | 0.569971 | 31.30203 | 91.462116 | 100.47969 | 43.718246 | 40.66745 | 89.31666 | 33.1977065 | 36.72737 | 4.31696 | 35.25259 | 34.85539 | 32.35286 | 33.85393 |
| NAALADL1 | 1.067747074 | 0.013947 | 0.530288 | 61.272059 | 28.707087 | 28.018374 | 31.98896 | 46.92398 | 69.68662 | 75.2481348 | 5.978873 | 17.26784 | 46.19305 | 13.69319 | 23.96508 | 33.85393 |
| COL9A3 | 1.06821609 | 0.01678 | 0.569971 | 49.950048 | 20.695807 | 24.153771 | 22.392272 | 19.81235 | 68.70512 | 45.3701989 | 3.416499 | 17.26784 | 23.09653 | 17.42769 | 21.56858 | 20.83319 |
| PGGHG | 1.070564789 | 0.011058 | 0.482381 | 266.40025 | 150.87911 | 76.325916 | 157.8122 | 158.4988 | 358.2481 | 49.7965598 | 71.74648 | 57.84727 | 94.81731 | 156.8492 | 65.90398 | 50.78089 |
| C5orf38 | 1.0870273 | 0.010718 | 0.474636 | 167.16616 | 56.078962 | 427.03867 | 154.61331 | 124.0879 | 206.1154 | 111.765612 | 55.51811 | 57.84727 | 138.5792 | 39.83473 | 124.6184 | 87.23897 |
| TUB | 1.087991522 | 0.025067 | 0.656554 | 33.300032 | 17.357774 | 9.6615083 | 19.193376 | 20.8551 | 42.20457 | 15.4922631 | 4.270624 | 5.180352 | 12.15607 | 23.65187 | 13.1808 | 6.510371 |
| FARP1 | 1.100595718 | 0.008898 | 0.430707 | 461.53844 | 336.47377 | 639.59185 | 480.90071 | 576.6436 | 877.4625 | 453.701989 | 361.2948 | 49.21335 | 247.9837 | 556.4414 | 184.5312 | 130.2074 |
| USP2 | 1.104500755 | 0.027937 | 0.678993 | 16.650016 | 18.692987 | 89.852027 | 79.972401 | 44.83847 | 84.40915 | 26.5581652 | 24.76962 | 10.3607 | 30.39016 | 19.91736 | 47.93017 | 10.41659 |
| DNAJC22 | 1.1131917 | 0.005517 | 0.333537 | 34.632033 | 18.025381 | 28.984525 | 22.392272 | 14.59857 | 24.53754 | 23.2383946 | 8.541248 | 3.453568 | 14.58728 | 17.42769 | 16.77556 | 6.510371 |
| TMEM200B | 1.115572281 | 0.032967 | 0.73333 | 37.296036 | 13.352134 | 24.153771 | 21.325974 | 17.72684 | 26.50055 | 34.3042968 | 4.270624 | 6.043745 | 29.17456 | 3.734506 | 4.793017 | 22.13526 |
| SLC27A2 | 1.140910866 | 0.038314 | 0.774251 | 28.638027 | 88.124083 | 223.18084 | 170.60779 | 281.5439 | 386.7117 | 368.494543 | 81.99598 | 14.67767 | 122.7763 | 56.01759 | 140.1957 | 186.1966 |
| RIBC2 | 1.141963368 | 0.009679 | 0.44769 | 11.322011 | 16.690167 | 21.255318 | 28.790064 | 33.36816 | 31.40806 | 66.3954131 | 10.2495 | 12.08749 | 17.01849 | 12.44835 | 7.189525 | 22.13526 |
| SPRY2 | 1.153680502 | 0.006541 | 0.36605 | 1680.9856 | 353.16394 | 268.58993 | 340.14928 | 311.7838 | 490.7509 | 307.632081 | 194.7404 | 240.023 | 362.2508 | 150.6251 | 246.8404 | 252.6024 |
| STON1 | 1.156519124 | 0.034543 | 0.739169 | 13.986013 | 3.3380334 | 11.59381 | 7.4640908 | 19.81235 | 8.833516 | 13.2790826 | 5.978873 | 5.180352 | 9.724853 | 3.734506 | 1.198254 | 3.906223 |
| PKDREJ | 1.167181553 | 0.046851 | 0.840656 | 13.986013 | 16.02256 | 19.323017 | 25.591168 | 29.19714 | 64.77911 | 3.31977065 | 9.395373 | 5.180352 | 19.44971 | 3.734506 | 20.37032 | 7.812445 |
| PODXL | 1.175633085 | 0.047353 | 0.844469 | 23.310022 | 218.30739 | 189.36556 | 171.67409 | 90.7197 | 164.8923 | 53.1163305 | 26.47787 | 12.08749 | 69.28958 | 169.2976 | 53.92144 | 15.62489 |
| FOXL1 | 1.19154055 | 0.003697 | 0.273446 | 51.94805 | 57.414175 | 38.646033 | 21.325974 | 13.55582 | 61.83461 | 46.4767892 | 19.64487 | 16.40445 | 13.37167 | 12.44835 | 35.94763 | 11.71867 |
| TLE6 | 1.200140087 | 0.006523 | 0.36605 | 105.8941 | 69.431095 | 68.596709 | 11.729285 | 37.53919 | 44.16758 | 49.7965598 | 26.47787 | 18.99463 | 42.54623 | 9.958682 | 27.55985 | 19.53111 |
| ITGA1 | 1.201794916 | 0.045041 | 0.836779 | 27.972027 | 56.746568 | 28.018374 | 13.861883 | 19.81235 | 25.51904 | 24.3449848 | 35.87324 | 10.3607 | 2.431213 | 17.42769 | 4.793017 | 1.302074 |
| LINC00672 | 1.211683539 | 0.018298 | 0.576919 | 65.934063 | 31.377514 | 38.646033 | 51.182337 | 85.50592 | 69.68662 | 6.63954131 | 23.91549 | 13.81427 | 43.76184 | 9.958682 | 29.95636 | 7.812445 |
| SEMA3B | 1.225649644 | 0.027139 | 0.672683 | 119.88011 | 174.24534 | 1113.0058 | 146.08292 | 182.4822 | 286.5985 | 151.60286 | 263.0704 | 43.1696 | 96.03292 | 169.2976 | 171.3504 | 53.38504 |
| ITGA2B | 1.240689087 | 0.040527 | 0.784047 | 24.642024 | 12.01692 | 11.59381 | 5.3314934 | 16.68408 | 13.74102 | 17.7054435 | 0 | 14.67767 | 8.509246 | 3.734506 | 7.189525 | 2.604148 |
| TNS1 | 1.24607896 | 0.020386 | 0.59784 | 3040.9589 | 876.56758 | 1524.586 | 3613.6862 | 2272.163 | 4697.467 | 433.783365 | 345.9205 | 667.4021 | 2200.248 | 353.5332 | 1775.813 | 605.4645 |
| PCLO | 1.247833105 | 0.017694 | 0.573917 | 201.13219 | 301.09062 | 243.47001 | 92.767985 | 254.4323 | 238.5049 | 98.4865294 | 256.2374 | 31.08211 | 105.7578 | 59.75209 | 49.12842 | 13.02074 |
| CEL | 1.247944066 | 0.034143 | 0.739169 | 236.43023 | 420.59221 | 1984.4738 | 1054.5694 | 816.4773 | 1190.562 | 231.277356 | 447.5614 | 124.3285 | 392.6409 | 316.1882 | 824.3989 | 36.45808 |
| SOD3 | 1.25222761 | 0.047771 | 0.848751 | 47.952046 | 4.0056401 | 16.424564 | 20.259675 | 8.342041 | 52.01959 | 9.95931196 | 2.562374 | 6.043745 | 17.01849 | 17.42769 | 8.38778 | 6.510371 |
| FAM189A2 | 1.267826114 | 0.004104 | 0.284762 | 65.268062 | 24.033841 | 99.513536 | 40.51935 | 63.60806 | 78.52014 | 33.1977065 | 19.64487 | 6.043745 | 40.11502 | 32.36572 | 13.1808 | 33.85393 |
| RAB26 | 1.291650612 | 0.033289 | 0.735475 | 47.952046 | 58.081782 | 551.67213 | 123.69065 | 127.2161 | 317.0251 | 196.973059 | 162.2837 | 12.08749 | 111.8358 | 38.58989 | 138.9975 | 33.85393 |
| SDC2 | 1.297697066 | 0.009427 | 0.441656 | 42.624041 | 45.397255 | 39.612184 | 66.110518 | 36.49643 | 131.5212 | 68.6085935 | 20.49899 | 12.95088 | 38.89941 | 12.44835 | 4.793017 | 61.19749 |
| SYDE2 | 1.303007485 | 0.015713 | 0.557019 | 26.640025 | 75.439555 | 47.341391 | 77.839804 | 46.92398 | 112.8727 | 29.8779359 | 41.85211 | 8.633921 | 18.2341 | 38.58989 | 35.94763 | 1.302074 |
| MYH7B | 1.311822075 | 0.022068 | 0.620724 | 64.602062 | 36.718368 | 32.849128 | 38.386753 | 37.53919 | 83.42765 | 3.31977065 | 17.0825 | 24.17498 | 27.95895 | 14.93802 | 16.77556 | 1.302074 |
| HSPB6 | 1.321339083 | 0.034243 | 0.739169 | 11.322011 | 2.6704267 | 15.458413 | 35.187856 | 5.213776 | 33.37106 | 18.8120337 | 4.270624 | 3.453568 | 13.37167 | 3.734506 | 11.98254 | 5.208297 |
| HUS1B | 1.333753948 | 0.046638 | 0.840239 | 4.6620045 | 3.3380334 | 7.7292067 | 10.662987 | 6.256531 | 9.815017 | 4.42636087 | 0.854125 | 1.726784 | 0 | 3.734506 | 3.594763 | 6.510371 |
| HIF3A | 1.364089071 | 0.036516 | 0.757515 | 39.294038 | 22.698627 | 4.8307542 | 22.392272 | 17.72684 | 44.16758 | 7.74613153 | 6.832998 | 16.40445 | 21.88092 | 3.734506 | 1.198254 | 2.604148 |
| TMEM121 | 1.376614015 | 0.017701 | 0.573917 | 28.638027 | 8.0112802 | 24.153771 | 6.3977921 | 11.47031 | 31.40806 | 40.9438381 | 8.541248 | 1.726784 | 9.724853 | 3.734506 | 7.189525 | 19.53111 |
| WNT3A | 1.379063342 | 0.004263 | 0.288892 | 196.47019 | 71.433915 | 282.11604 | 133.28734 | 82.37766 | 190.4113 | 127.257875 | 16.22837 | 37.98925 | 159.2445 | 21.1622 | 63.50747 | 59.89541 |
| MLXIPL | 1.383066742 | 0.025844 | 0.665835 | 8.6580083 | 6.6760669 | 20.289167 | 19.193376 | 18.76959 | 50.05659 | 6.63954131 | 8.541248 | 2.590176 | 10.94046 | 4.979341 | 14.37905 | 1.302074 |
| FOXP2 | 1.384360972 | 0.016003 | 0.563844 | 26.640025 | 30.042301 | 17.390715 | 73.574609 | 138.6864 | 111.8912 | 30.9845261 | 13.666 | 20.72141 | 57.13351 | 6.224176 | 10.78429 | 32.55186 |
| MYB | 1.395430278 | 0.039857 | 0.777398 | 6.6600064 | 7.3436735 | 26.086072 | 105.56357 | 86.54868 | 113.8542 | 70.821774 | 12.81187 | 2.590176 | 23.09653 | 14.93802 | 35.94763 | 46.87467 |
| PNCK | 1.396791154 | 0.002483 | 0.227529 | 232.43422 | 54.076142 | 97.581234 | 82.104998 | 126.1734 | 290.5245 | 57.5426913 | 44.41449 | 23.31159 | 96.03292 | 22.40704 | 63.50747 | 57.29127 |
| DOK7 | 1.399768895 | 0.000108 | 0.030846 | 151.84815 | 165.56646 | 685.96709 | 232.45311 | 204.38 | 353.3406 | 438.209726 | 94.80785 | 69.07137 | 139.7948 | 146.8906 | 146.187 | 128.9053 |
| AC008687.4 | 1.400637788 | 0.041365 | 0.79615 | 7.326007 | 11.349314 | 23.18762 | 10.662987 | 34.41092 | 19.63003 | 4.42636087 | 18.79075 | 1.726784 | 4.862426 | 1.244835 | 7.189525 | 1.302074 |
| KLRF1 | 1.402637256 | 0.034543 | 0.739169 | 1.3320013 | 4.0056401 | 38.646033 | 9.5966881 | 13.55582 | 14.72253 | 21.0252141 | 9.395373 | 2.590176 | 6.078033 | 4.979341 | 5.991271 | 3.906223 |
| TBC1D30 | 1.407611813 | 0.026383 | 0.672023 | 9.9900096 | 34.715548 | 109.17504 | 63.977921 | 50.05225 | 64.77911 | 107.339251 | 46.12274 | 2.590176 | 3.64682 | 53.52792 | 20.37032 | 15.62489 |
| AC004233.2 | 1.420655487 | 8.81E-07 | 0.001284 | 63.936061 | 94.132543 | 62.799804 | 34.121558 | 72.99286 | 61.83461 | 45.3701989 | 26.47787 | 21.5848 | 13.37167 | 26.14154 | 27.55985 | 24.73941 |
| SPON2 | 1.421404153 | 0.013691 | 0.527713 | 177.82217 | 55.411355 | 569.06284 | 388.13272 | 822.7338 | 350.3961 | 219.104863 | 92.24548 | 19.85802 | 374.4068 | 105.811 | 86.2743 | 148.4365 |
| CRYBB3 | 1.42160469 | 0.03433 | 0.739169 | 76.590073 | 11.349314 | 3.8646033 | 13.861883 | 13.55582 | 8.833516 | 5.53295109 | 6.832998 | 6.907137 | 10.94046 | 6.224176 | 8.38778 | 3.906223 |
| KCNQ1 | 1.430160835 | 0.006111 | 0.353503 | 61.938059 | 131.51852 | 499.49998 | 475.56921 | 291.9714 | 649.7541 | 516.777632 | 187.9075 | 19.85802 | 222.456 | 105.811 | 179.7381 | 119.7908 |
| FAM72C | 1.432636944 | 0.033926 | 0.739169 | 84.582081 | 93.464936 | 11.59381 | 4.2651947 | 11.47031 | 11.77802 | 29.8779359 | 11.10362 | 4.31696 | 12.15607 | 13.69319 | 9.586034 | 28.64563 |
| ARHGAP44 | 1.449304787 | 0.026637 | 0.672647 | 14.652014 | 38.053581 | 5.796905 | 15.99448 | 23.98337 | 182.5593 | 53.1163305 | 18.79075 | 8.633921 | 30.39016 | 23.65187 | 14.37905 | 9.11452 |
| SLIT2 | 1.46810562 | 0.02026 | 0.59784 | 209.1242 | 64.757849 | 38.646033 | 14.928182 | 15.64133 | 32.38956 | 34.3042968 | 16.22837 | 24.17498 | 27.95895 | 38.58989 | 2.396508 | 18.22904 |
| KRT8 | 1.472455037 | 0.007262 | 0.386176 | 4955.7107 | 6047.1814 | 22366.392 | 5788.9355 | 5320.137 | 11042.88 | 17583.7186 | 9308.252 | 390.2532 | 1746.827 | 4283.478 | 4671.993 | 2179.672 |
| MEIS3 | 1.479476923 | 0.037145 | 0.764413 | 112.55411 | 22.698627 | 2.8984525 | 4.2651947 | 27.11163 | 16.68553 | 12.1724924 | 7.687123 | 4.31696 | 15.80289 | 8.713847 | 8.38778 | 16.92696 |
| BST1 | 1.481678039 | 0.021715 | 0.620275 | 20.64602 | 8.6788869 | 4.8307542 | 3.198896 | 7.299286 | 15.70403 | 4.42636087 | 5.124749 | 2.590176 | 4.862426 | 1.244835 | 4.793017 | 1.302074 |
| ZNF90 | 1.487924791 | 0.000922 | 0.12108 | 51.282049 | 30.709908 | 14.492262 | 49.049739 | 90.7197 | 67.72362 | 18.8120337 | 7.687123 | 24.17498 | 20.66531 | 13.69319 | 16.77556 | 15.62489 |
| FES | 1.510394601 | 0.002525 | 0.228549 | 11.988011 | 14.01974 | 33.815279 | 34.121558 | 46.92398 | 100.1132 | 65.2888229 | 22.20724 | 7.770529 | 23.09653 | 14.93802 | 11.98254 | 11.71867 |
| FP565260.3 | 1.516478346 | 0.031833 | 0.722441 | 97.236093 | 32.045121 | 5.796905 | 92.767985 | 25.02612 | 20.61154 | 8.85272174 | 0.854125 | 21.5848 | 18.2341 | 19.91736 | 20.37032 | 3.906223 |
| G0S2 | 1.536470523 | 0.013813 | 0.529405 | 71.928069 | 116.83117 | 223.18084 | 92.767985 | 135.5582 | 486.8249 | 378.453855 | 115.3068 | 20.72141 | 55.9179 | 11.20352 | 219.2805 | 22.13526 |
| CD177 | 1.555795984 | 0.032882 | 0.732997 | 4.6620045 | 8.0112802 | 10.627659 | 34.121558 | 46.92398 | 104.0392 | 5.53295109 | 17.0825 | 3.453568 | 13.37167 | 3.734506 | 17.97381 | 6.510371 |
| SV2A | 1.559711698 | 0.016664 | 0.569971 | 97.236093 | 26.036661 | 14.492262 | 23.458571 | 7.299286 | 45.14908 | 8.85272174 | 1.70825 | 14.67767 | 14.58728 | 7.469012 | 21.56858 | 5.208297 |

Continue on the next page…

| gene_id | log2FoldChange | pvalue | padj | nonTO-01 | nonTO-02 | nonTO-03 | nonTO-04 | nonTO-05 | nonTO-06 | nonTO-07 | TO-01 | TO-02 | TO-03 | TO-04 | TO-05 | TO-06 |
|---|---|---|---|---|---|---|---|---|---|---|---|---|---|---|---|---|
| ATAD3C | 1.595897871 | 0.006424 | 0.365317 | 31.30203 | 28.039481 | 98.547385 | 21.325974 | 52.13776 | 66.74212 | 28.7713457 | 40.14386 | 0.863392 | 10.94046 | 19.91736 | 5.991271 | 14.32282 |
| ZBTB10 | 1.621188717 | 0.005241 | 0.324151 | 56.610054 | 98.805789 | 74.393614 | 27.723766 | 19.81235 | 137.4102 | 32.0911163 | 50.39336 | 2.590176 | 29.17456 | 17.42769 | 16.77556 | 7.812445 |
| LOX | 1.639605684 | 0.008499 | 0.419397 | 861.80482 | 107.48468 | 57.002899 | 105.56357 | 106.361 | 281.691 | 44.2636087 | 53.80986 | 42.30621 | 177.4786 | 34.85539 | 57.5162 | 65.10371 |
| KISS1R | 1.653945403 | 0.039755 | 0.777398 | 106.5601 | 19.360594 | 1.9323017 | 11.729285 | 4.171021 | 7.852014 | 17.7054435 | 17.93662 | 3.453568 | 12.15607 | 2.489671 | 3.594763 | 6.510371 |
| EYA1 | 1.666899613 | 2.24E-05 | 0.010842 | 46.620045 | 38.721188 | 30.916827 | 39.453051 | 35.45367 | 76.55713 | 32.0911163 | 5.124749 | 20.72141 | 20.66531 | 8.713847 | 19.17207 | 6.510371 |
| DOC2B | 1.670587536 | 0.032902 | 0.732997 | 1.9980019 | 2.0028201 | 19.323017 | 40.51935 | 25.02612 | 44.16758 | 17.7054435 | 7.687123 | 0 | 7.293639 | 7.469012 | 15.57731 | 2.604148 |
| CHDH | 1.679584463 | 0.001449 | 0.170308 | 54.612052 | 8.6788869 | 57.002899 | 18.127078 | 35.45367 | 93.24266 | 46.4767892 | 9.395373 | 5.180352 | 12.15607 | 19.91736 | 28.7581 | 9.11452 |
| DGCR6 | 1.729884444 | 0.010296 | 0.464721 | 39.294038 | 14.01974 | 19.323017 | 20.259675 | 70.90735 | 91.27966 | 5.53295109 | 0.854125 | 7.770529 | 14.58728 | 28.63121 | 9.586034 | 6.510371 |
| HHEX | 1.735527674 | 0.004936 | 0.31684 | 3.3300032 | 12.01692 | 19.323017 | 12.795584 | 5.213776 | 17.66703 | 9.95931196 | 4.270624 | 2.590176 | 0 | 2.489671 | 4.793017 | 6.510371 |
| VMO1 | 1.75344315 | 0.011489 | 0.4902 | 5.3280051 | 7.3436735 | 112.0735 | 29.856363 | 35.45367 | 60.85311 | 57.5426913 | 16.22837 | 7.770529 | 14.58728 | 0 | 22.76683 | 16.92696 |
| EPX | 1.768577629 | 0.004493 | 0.300276 | 14.652014 | 9.3464936 | 5.796905 | 11.729285 | 15.64133 | 24.53754 | 2.21318044 | 2.562374 | 6.043745 | 4.862426 | 1.244835 | 4.793017 | 1.302074 |
| TENM3 | 1.775352556 | 0.015221 | 0.548937 | 225.77422 | 13.352134 | 13.526112 | 18.127078 | 34.41092 | 34.35256 | 4.42636087 | 10.2495 | 8.633921 | 21.88092 | 27.38638 | 10.78429 | 7.812445 |
| CACNB4 | 1.823416928 | 0.000105 | 0.030551 | 20.64602 | 24.701447 | 14.492262 | 7.4640908 | 10.42755 | 19.63003 | 15.4922631 | 6.832998 | 4.31696 | 3.64682 | 2.489671 | 5.991271 | 3.906223 |
| CHL1 | 1.823956166 | 0.034943 | 0.744336 | 2.6640025 | 19.360594 | 9.6615083 | 5.3314934 | 6.256531 | 49.07509 | 6.63954131 | 10.2495 | 0 | 7.293639 | 0 | 3.594763 | 2.604148 |
| PLCH1 | 1.824775843 | 0.012851 | 0.514478 | 27.972027 | 6.0084602 | 5.796905 | 5.3314934 | 4.171021 | 6.870512 | 22.1318044 | 6.832998 | 5.180352 | 0 | 2.489671 | 1.198254 | 2.604148 |
| C9orf131 | 1.834517167 | 0.017029 | 0.569971 | 16.650016 | 14.687347 | 18.356866 | 15.99448 | 5.213776 | 14.72253 | 0 | 5.124749 | 0.863392 | 2.431213 | 8.713847 | 3.594763 | 0 |
| IFITM2 | 1.835437593 | 1.8E-06 | 0.002016 | 327.67231 | 250.35251 | 472.44776 | 322.0222 | 271.1163 | 598.7161 | 735.882495 | 195.5946 | 31.94551 | 130.0699 | 67.22111 | 150.98 | 139.3219 |
| DMRT2 | 1.835904579 | 8.18E-05 | 0.025358 | 40.626039 | 117.49878 | 72.461312 | 145.01662 | 71.95011 | 222.8009 | 135.004007 | 7.687123 | 19.85802 | 32.82138 | 38.58989 | 71.89525 | 23.43734 |
| B3GAT1 | 1.851515049 | 0.005422 | 0.330534 | 5.9940057 | 10.0141 | 8.6953575 | 14.928182 | 8.342041 | 8.833516 | 6.63954131 | 0 | 0 | 6.078033 | 2.489671 | 4.793017 | 2.604148 |
| MRAP | 1.863841417 | 0.038751 | 0.774984 | 3.3300032 | 2.0028201 | 7.7292067 | 3.198896 | 6.256531 | 4.907509 | 1.10659022 | 0 | 0.863392 | 2.431213 | 1.244835 | 2.396508 | 0 |
| CDH4 | 1.864747252 | 0.001188 | 0.149247 | 22.644022 | 4.0056401 | 17.390715 | 29.856363 | 18.76959 | 9.815017 | 37.6240674 | 5.124749 | 0.863392 | 6.078033 | 6.224176 | 4.793017 | 10.41659 |
| SOBP | 1.871690334 | 0.039467 | 0.777398 | 1.9980019 | 35.383154 | 45.409089 | 18.127078 | 11.47031 | 173.7258 | 13.2790826 | 3.416499 | 0 | 26.74334 | 18.67253 | 20.37032 | 1.302074 |
| CCDC8 | 1.896737966 | 0.007445 | 0.388152 | 482.18446 | 77.442376 | 27.052223 | 140.75143 | 61.52255 | 292.4875 | 150.49627 | 9.395373 | 17.26784 | 79.01443 | 38.58989 | 3.594763 | 136.7178 |
| SYCP2 | 1.901665657 | 0.006138 | 0.353503 | 350.31634 | 41.391615 | 28.984525 | 15.99448 | 23.98337 | 90.29816 | 14.3856728 | 9.395373 | 31.08211 | 37.6838 | 11.20352 | 26.36159 | 14.32282 |
| ZNF423 | 1.9622323 | 0.014459 | 0.534801 | 10.65601 | 2.0028201 | 2.8984525 | 4.2651947 | 5.213776 | 14.72253 | 5.53295109 | 0.854125 | 0.863392 | 3.64682 | 1.244835 | 3.594763 | 0 |
| NUP210 | 1.968577312 | 0.001838 | 0.19368 | 43.290041 | 65.425455 | 259.89457 | 37.320454 | 27.11163 | 112.8727 | 100.69971 | 46.97686 | 2.590176 | 17.01849 | 37.34506 | 33.55112 | 3.906223 |
| CKM | 1.979495025 | 0.043996 | 0.827123 | 17.316017 | 3.3380334 | 4.8307542 | 2.1325974 | 6.256531 | 11.77802 | 0 | 0.854125 | 0.863392 | 3.64682 | 0 | 4.793017 | 0 |
| LDOC1 | 1.985807173 | 0.009479 | 0.442668 | 129.20412 | 15.354954 | 84.055122 | 92.767985 | 44.83847 | 64.77911 | 139.430367 | 4.270624 | 0.863392 | 0 | 32.36572 | 35.94763 | 50.78089 |
| SDK1 | 1.986227918 | 0.017216 | 0.571147 | 10.65601 | 5.3408535 | 5.796905 | 3.198896 | 42.75296 | 70.66812 | 7.74613153 | 8.541248 | 4.31696 | 2.431213 | 13.69319 | 2.396508 | 0 |
| HAPLN2 | 1.99047851 | 0.041837 | 0.803122 | 3.9960038 | 2.0028201 | 4.8307542 | 7.4640908 | 8.342041 | 21.59304 | 1.10659022 | 0 | 0 | 2.431213 | 0 | 5.991271 | 2.604148 |
| KCNE3 | 2.032847542 | 0.004214 | 0.287825 | 9.3240089 | 4.0056401 | 17.390715 | 43.718246 | 23.98337 | 78.52014 | 15.4922631 | 8.541248 | 0 | 13.37167 | 6.224176 | 8.38778 | 3.906223 |
| AMOT | 2.078631411 | 0.003749 | 0.274565 | 49.284047 | 4.6732468 | 52.172145 | 17.060779 | 17.72684 | 109.9282 | 26.5581652 | 20.49899 | 0 | 15.80289 | 6.224176 | 8.38778 | 5.208297 |
| TSPO2 | 2.085380983 | 0.004826 | 0.312497 | 33.966033 | 7.3436735 | 7.7292067 | 5.3314934 | 10.42755 | 16.68553 | 3.31977065 | 4.270624 | 0.863392 | 4.862426 | 0 | 5.991271 | 1.302074 |
| SGCE | 2.096894368 | 0.013797 | 0.529405 | 181.15217 | 70.766309 | 56.036748 | 70.375713 | 14.59857 | 64.77911 | 18.8120337 | 7.687123 | 0.863392 | 38.89941 | 47.30374 | 0 | 1.302074 |
| ARL13A | 2.108786967 | 0.019512 | 0.589807 | 11.988011 | 4.0056401 | 0 | 5.3314934 | 6.256531 | 14.72253 | 2.21318044 | 1.70825 | 0 | 1.215607 | 2.489671 | 2.396508 | 1.302074 |
| ZDHHC2 | 2.130487723 | 0.000354 | 0.065591 | 47.952046 | 188.93269 | 111.10735 | 115.16026 | 214.8076 | 364.1371 | 357.42864 | 52.95574 | 7.770529 | 93.60171 | 94.60748 | 8.38778 | 16.92696 |
| JAM3 | 2.139494504 | 0.001118 | 0.142926 | 259.07425 | 26.036661 | 23.18762 | 14.928182 | 13.55582 | 78.52014 | 24.3449848 | 10.2495 | 19.85802 | 10.94046 | 19.91736 | 14.37905 | 10.41659 |
| HCLS1 | 2.142986884 | 0.000331 | 0.065231 | 15.318015 | 104.14664 | 254.09767 | 65.04422 | 187.6959 | 162.9293 | 165.988533 | 76.87123 | 6.043745 | 14.58728 | 54.77275 | 15.57731 | 16.92696 |
| ITGB2 | 2.199810707 | 0.000776 | 0.110386 | 9.3240089 | 130.85091 | 26.086072 | 13.861883 | 18.76959 | 18.64853 | 18.8120337 | 3.416499 | 4.31696 | 3.64682 | 13.69319 | 11.98254 | 7.812445 |
| SUPT4H1 | 2.221973586 | 0.021279 | 0.615155 | 7.326007 | 4.0056401 | 5.796905 | 3.198896 | 2.08551 | 3.926007 | 1.10659022 | 1.70825 | 1.726784 | 1.215607 | 0 | 0 | 0 |
| GPR18 | 2.229764533 | 0.048027 | 0.849221 | 1.9980019 | 4.0056401 | 11.59381 | 9.5966881 | 5.213776 | 3.926007 | 0 | 0 | 1.726784 | 0 | 4.979341 | 0 | 0 |
| NDN | 2.231140811 | 0.009089 | 0.43324 | 4.6620045 | 2.6704267 | 22.221469 | 66.110518 | 96.97623 | 305.247 | 206.932371 | 15.37425 | 2.590176 | 3.64682 | 26.14154 | 55.11969 | 26.04148 |
| RUNX3 | 2.266360766 | 0.00081 | 0.110386 | 418.2484 | 24.033841 | 394.18954 | 116.22656 | 150.1567 | 332.7291 | 130.577646 | 34.16499 | 0.863392 | 34.03698 | 104.5662 | 83.8778 | 22.13526 |
| SFMBT2 | 2.311823532 | 0.033551 | 0.736561 | 40.626039 | 2.6704267 | 7.7292067 | 2.1325974 | 1.042755 | 2.944505 | 0 | 2.562374 | 3.453568 | 1.215607 | 1.244835 | 1.198254 | 0 |
| NKX2-1 | 2.338443726 | 0.019193 | 0.58381 | 701.96467 | 456.64297 | 163.27949 | 313.49181 | 275.2874 | 915.7411 | 447.062448 | 43.56036 | 0 | 370.76 | 121.9939 | 8.38778 | 10.41659 |
| CD248 | 2.353430601 | 0.010769 | 0.475458 | 6.6600064 | 0.6676067 | 6.7630558 | 29.856363 | 54.22327 | 114.8357 | 5.53295109 | 1.70825 | 0.863392 | 21.88092 | 3.734506 | 3.594763 | 5.208297 |
| PSAPL1 | 2.357146437 | 0.011271 | 0.488749 | 0 | 5.3408535 | 23.18762 | 135.41993 | 55.26602 | 84.40915 | 7.74613153 | 1.70825 | 0.863392 | 15.80289 | 6.224176 | 8.38778 | 19.53111 |
| ARHGAP6 | 2.36262673 | 5.41E-05 | 0.017923 | 8.6580083 | 18.692987 | 31.882977 | 6.3977921 | 15.64133 | 27.48205 | 13.2790826 | 0.854125 | 1.726784 | 6.078033 | 6.224176 | 3.594763 | 2.604148 |
| SLCO4C1 | 2.376715061 | 0.015579 | 0.554978 | 0.6660006 | 2.0028201 | 2.8984525 | 28.790064 | 35.45367 | 90.29816 | 4.42636087 | 2.562374 | 0 | 3.64682 | 12.44835 | 3.594763 | 5.208297 |
| C5AR1 | 2.386927045 | 0.00357 | 0.270811 | 36.630035 | 28.039481 | 80.190519 | 157.8122 | 208.551 | 474.0653 | 16.5988533 | 1.70825 | 6.907137 | 91.17049 | 29.87605 | 32.35286 | 2.604148 |
| IFITM1 | 2.399141033 | 1.33E-06 | 0.001757 | 586.08056 | 134.85655 | 116.90425 | 213.25974 | 236.7054 | 947.1492 | 749.161578 | 40.99799 | 62.16423 | 103.3266 | 54.77275 | 69.49875 | 154.9468 |
| GNAT1 | 2.403267139 | 0.023334 | 0.634282 | 3.3300032 | 14.687347 | 6.7630558 | 9.5966881 | 5.213776 | 2.944505 | 0 | 0 | 0 | 1.215607 | 1.244835 | 4.793017 | 0 |
| FMO3 | 2.407560706 | 0.046674 | 0.840239 | 0 | 42.726828 | 34.78143 | 24.52487 | 13.55582 | 3.926007 | 4.42636087 | 13.666 | 0 | 0 | 0 | 5.991271 | 0 |
| EDIL3 | 2.478092788 | 0.009184 | 0.435853 | 94.572091 | 4.0056401 | 10.627659 | 5.3314934 | 10.42755 | 27.48205 | 5.53295109 | 15.37425 | 0 | 3.64682 | 2.489671 | 2.396508 | 0 |
| CAPN9 | 2.478833509 | 4.56E-05 | 0.015828 | 13.320013 | 6.6760669 | 19.323017 | 44.784545 | 6.256531 | 47.11208 | 16.5988533 | 5.978873 | 2.590176 | 3.64682 | 3.734506 | 3.594763 | 3.906223 |
| KCNU1 | 2.483900113 | 0.018333 | 0.576919 | 5.9940057 | 4.0056401 | 0.9661508 | 5.3314934 | 1.042755 | 23.55604 | 1.10659022 | 0.854125 | 1.726784 | 0 | 1.244835 | 0 | 2.604148 |
| RNF144A | 2.49334988 | 0.00339 | 0.267024 | 21.978021 | 0 | 5.796905 | 5.3314934 | 16.68408 | 21.59304 | 15.4922631 | 3.416499 | 0 | 3.64682 | 2.489671 | 1.198254 | 2.604148 |
| VWA5B2 | 2.552771134 | 0.007649 | 0.393827 | 5.3280051 | 3.3380334 | 3.8646033 | 2.1325974 | 5.213776 | 13.74102 | 3.31977065 | 0.854125 | 0.863392 | 3.64682 | 0 | 0 | 0 |
| GYPC | 2.558149843 | 0.00075 | 0.108138 | 29.970029 | 4.6732468 | 7.7292067 | 8.5303895 | 7.299286 | 15.70403 | 8.85272174 | 0 | 2.590176 | 2.431213 | 0 | 5.991271 | 1.302074 |
| CLGN | 2.568603282 | 0.000294 | 0.060381 | 20.64602 | 24.701447 | 9.6615083 | 155.67961 | 201.2517 | 56.9271 | 11.0659022 | 18.79075 | 8.633921 | 13.37167 | 13.69319 | 13.1808 | 1.302074 |
| ADRA2B | 2.622055563 | 0.026073 | 0.668244 | 1.9980019 | 1.3352134 | 1.9323017 | 1.0662987 | 1.042755 | 9.815017 | 6.63954131 | 0.854125 | 0 | 0 | 1.244835 | 1.198254 | 0 |
| POU5F2 | 2.622602404 | 0.019084 | 0.582921 | 1.3320013 | 2.6704267 | 6.7630558 | 15.99448 | 4.171021 | 6.870512 | 0 | 0 | 2.590176 | 0 | 1.244835 | 1.198254 | 0 |
| STAC | 2.627825835 | 0.000693 | 0.100899 | 23.310022 | 16.02256 | 17.390715 | 18.127078 | 10.42755 | 22.57454 | 43.1570185 | 12.81187 | 0 | 1.215607 | 6.224176 | 0 | 0 |
| NPFFR2 | 2.664909026 | 0.039237 | 0.777398 | 1.9980019 | 14.01974 | 15.458413 | 1.0662987 | 1.042755 | 0.981502 | 13.2790826 | 5.978873 | 0 | 0 | 0 | 0 | 0 |
| PRDM16 | 2.674249023 | 0.017726 | 0.573917 | 128.53812 | 78.109982 | 41.544486 | 157.8122 | 135.5582 | 295.432 | 79.6744957 | 0 | 0 | 106.9734 | 3.734506 | 3.594763 | 9.11452 |
| PAK5 | 2.69590707 | 0.00355 | 0.270811 | 31.30203 | 0.6676067 | 6.7630558 | 6.3977921 | 10.42755 | 25.51904 | 3.31977065 | 1.70825 | 0.863392 | 0 | 4.979341 | 0 | 3.906223 |
| KLRB1 | 2.707055873 | 0.020592 | 0.602472 | 4.6620045 | 4.0056401 | 2.8984525 | 2.1325974 | 5.213776 | 5.88901 | 0 | 0 | 0.863392 | 2.431213 | 0 | 0 | 0 |
| HSD17B13 | 2.71379766 | 0.006503 | 0.36605 | 0.6660006 | 2.6704267 | 5.796905 | 4.2651947 | 6.256531 | 7.852014 | 15.4922631 | 0.854125 | 0 | 2.431213 | 0 | 2.396508 | 0 |
| SFTA3 | 2.753680541 | 0.000572 | 0.087698 | 58.608056 | 26.704267 | 25.119922 | 29.856363 | 23.98337 | 103.0577 | 37.6240674 | 1.70825 | 0.863392 | 26.74334 | 9.958682 | 0 | 0 |
| FAIM2 | 2.77367251 | 4.43E-06 | 0.003347 | 10.65601 | 24.033841 | 50.239843 | 54.381233 | 61.52255 | 16.68553 | 92.9535783 | 9.395373 | 0 | 7.293639 | 11.20352 | 5.991271 | 5.208297 |
| C14orf39 | 2.776959749 | 0.022535 | 0.62835 | 0.6660006 | 10.0141 | 15.458413 | 0 | 1.042755 | 1.963003 | 12.1724924 | 0.854125 | 1.726784 | 0 | 0 | 2.396508 | 0 |
| SOX5 | 2.859170679 | 0.038238 | 0.773788 | 16.650016 | 0 | 0.9661508 | 3.198896 | 2.08551 | 12.75952 | 0 | 0 | 1.726784 | 1.215607 | 1.244835 | 0 | 0 |
| SLC30A8 | 2.876455645 | 0.035913 | 0.751104 | 1.9980019 | 0.6676067 | 0 | 2.1325974 | 7.299286 | 3.926007 | 4.42636087 | 0 | 0 | 2.431213 | 0 | 0 | 0 |
| PEX5L | 2.927833227 | 0.031085 | 0.712115 | 5.3280051 | 5.3408535 | 4.8307542 | 1.0662987 | 1.042755 | 2.944505 | 0 | 1.70825 | 0 | 0 | 0 | 0 | 0 |
| HRASLS5 | 2.964538817 | 0.00559 | 0.33641 | 3.9960038 | 16.02256 | 3.8646033 | 11.729285 | 12.51306 | 7.852014 | 0 | 3.416499 | 0 | 0 | 2.489671 | 0 | 0 |
| SYNDIG1L | 3.040673855 | 0.021207 | 0.615155 | 2.6640025 | 1.3352134 | 1.9323017 | 3.198896 | 0 | 4.907509 | 2.21318044 | 0 | 0 | 1.215607 | 0 | 0 | 0 |
| AQP4 | 3.103512752 | 0.000786 | 0.110386 | 1.9980019 | 11.349314 | 45.409089 | 11.729285 | 9.384796 | 38.27857 | 3.31977065 | 3.416499 | 0 | 0 | 0 | 3.594763 | 5.208297 |
| BGN | 3.133045471 | 0.00514 | 0.322914 | 161.17215 | 0.6676067 | 5.796905 | 8.5303895 | 3.128265 | 102.0762 | 0 | 0 | 7.770529 | 7.293639 | 3.734506 | 3.594763 | 5.208297 |
| CHST9 | 3.149519382 | 4.48E-05 | 0.015828 | 18.648018 | 101.47622 | 348.78045 | 83.171297 | 162.6698 | 448.5463 | 107.339251 | 79.4336 | 0.863392 | 7.293639 | 13.69319 | 17.97381 | 2.604148 |
| ELN | 3.165811052 | 0.014462 | 0.534801 | 27.306026 | 2.6704267 | 74.393614 | 404.1272 | 410.8455 | 3241.9 | 17.7054435 | 4.270624 | 3.453568 | 108.189 | 4.979341 | 106.6446 | 171.8738 |
| CHST10 | 3.198878558 | 0.005611 | 0.33641 | 6.6600064 | 1.3352134 | 0.9661508 | 1.0662987 | 3.128265 | 15.70403 | 21.0252141 | 0 | 0 | 0 | 1.244835 | 2.396508 | 1.302074 |
| CD300A | 3.262875909 | 0.030336 | 0.704335 | 0.6660006 | 0 | 2.8984525 | 3.198896 | 3.128265 | 16.68553 | 0 | 0 | 0 | 1.215607 | 0 | 1.198254 | 0 |
| UGT1A10 | 3.323622747 | 0.027144 | 0.672683 | 0 | 0 | 5.796905 | 3.198896 | 15.64133 | 0.981502 | 2.21318044 | 0 | 0 | 1.215607 | 0 | 0 | 1.302074 |
| TRIM61 | 3.536205575 | 0.014998 | 0.544808 | 1.3320013 | 4.6732468 | 0 | 2.1325974 | 4.171021 | 19.63003 | 0 | 0 | 0 | 1.215607 | 1.244835 | 0 | 0 |
| COL5A1 | 3.585230474 | 0.029878 | 0.698753 | 460.87244 | 0 | 1.9323017 | 0 | 8.342041 | 63.79761 | 0 | 1.70825 | 0.863392 | 8.509246 | 2.489671 | 14.37905 | 10.41659 |
| L3MBTL4 | 3.605234992 | 0.002991 | 0.249163 | 35.298034 | 0 | 9.6615083 | 2.1325974 | 13.55582 | 17.66703 | 95.1667588 | 6.832998 | 0 | 0 | 0 | 0 | 5.208297 |
| LEF1 | 3.65099865 | 0.000405 | 0.071072 | 137.86213 | 1.3352134 | 73.427463 | 4.2651947 | 15.64133 | 32.38956 | 40.9438381 | 5.978873 | 0 | 0 | 13.69319 | 0 | 1.302074 |
| ATP8B4 | 3.719949471 | 0.014777 | 0.540972 | 0 | 2.0028201 | 1.9323017 | 4.2651947 | 4.171021 | 13.74102 | 0 | 0.854125 | 0 | 0 | 0 | 0 | 0 |
| NPPC | 3.753885556 | 0.006609 | 0.366109 | 211.1222 | 3.3380334 | 4.8307542 | 1.0662987 | 3.128265 | 106.9837 | 0 | 7.687123 | 0.863392 | 1.215607 | 0 | 5.991271 | 5.208297 |
| COL14A1 | 3.810827417 | 0.014384 | 0.534801 | 338.99432 | 2.0028201 | 0.9661508 | 1.0662987 | 0 | 43.18608 | 0 | 0.854125 | 1.726784 | 9.724853 | 1.244835 | 2.396508 | 7.812445 |
| LDHC | 3.85107267 | 0.039728 | 0.777398 | 1.3320013 | 0 | 8.6953575 | 0 | 0 | 0 | 29.8779359 | 0 | 0 | 1.215607 | 1.244835 | 0 | 0 |
| NLGN4X | 3.959540445 | 0.002105 | 0.203973 | 0 | 84.118442 | 112.0735 | 4.2651947 | 2.08551 | 1.963003 | 5.53295109 | 4.270624 | 0 | 0 | 1.244835 | 5.991271 | 0 |
| DMRT3 | 3.970407766 | 0.005497 | 0.333537 | 3.9960038 | 0 | 3.8646033 | 13.861883 | 0 | 36.31556 | 5.53295109 | 0 | 0 | 1.215607 | 0 | 2.396508 | 0 |
| MOXD1 | 4.125615094 | 0.017576 | 0.573917 | 49.950048 | 0 | 0 | 0 | 1.042755 | 14.72253 | 1.10659022 | 0.854125 | 0 | 2.431213 | 0 | 0 | 0 |
| PEG3 | 4.154786006 | 0.03749 | 0.765694 | 0.6660006 | 0 | 6.7630558 | 0 | 2.08551 | 15.70403 | 0 | 0 | 0 | 0 | 0 | 0 | 0 |
| DPY19L2 | 4.155778711 | 0.004745 | 0.311412 | 53.280051 | 0 | 0 | 15.99448 | 2.08551 | 76.55713 | 1.10659022 | 0 | 3.453568 | 0 | 0 | 3.594763 | 0 |
| PI15 | 4.201864419 | 0.016737 | 0.569971 | 21.31202 | 0 | 5.796905 | 1.0662987 | 0 | 7.852014 | 0 | 0.854125 | 0 | 0 | 0 | 0 | 0 |
| DPYSL3 | 4.219606303 | 2.37E-08 | 0.000105 | 24.642024 | 8.6788869 | 27.052223 | 9.5966881 | 68.82184 | 85.39065 | 245.663028 | 0.854125 | 2.590176 | 3.64682 | 4.979341 | 5.991271 | 3.906223 |
| TMEM47 | 4.323593202 | 0.000818 | 0.110386 | 93.240089 | 1.3352134 | 0 | 3.198896 | 0 | 53.00109 | 13.2790826 | 2.562374 | 1.726784 | 0 | 1.244835 | 0 | 1.302074 |
| SPARCL1 | 4.424029383 | 0.001277 | 0.158848 | 347.65233 | 2.0028201 | 8.6953575 | 5.3314934 | 2.08551 | 63.79761 | 2.21318044 | 0 | 5.180352 | 2.431213 | 1.244835 | 8.38778 | 0 |
| FREM2 | 4.432537458 | 0.000515 | 0.084293 | 67.266064 | 8.0112802 | 117.8704 | 1.0662987 | 15.64133 | 224.7639 | 6.63954131 | 11.10362 | 0 | 0 | 3.734506 | 2.396508 | 0 |
| UGT8 | 4.57069043 | 0.002889 | 0.249028 | 35.964034 | 0 | 5.796905 | 0 | 10.42755 | 11.77802 | 1.10659022 | 0 | 0 | 0 | 0 | 0 | 2.604148 |
| U2AF1L5 | 4.866155849 | 0.030967 | 0.710858 | 33.300032 | 0 | 0 | 1.0662987 | 0 | 0 | 6.63954131 | 0 | 0 | 0 | 0 | 0 | 0 |
| TAP2 | 5.030300468 | 0.018658 | 0.578002 | 0 | 0 | 0 | 13.861883 | 0 | 23.55604 | 8.85272174 | 0 | 0 | 0 | 0 | 0 | 0 |
| SPOCK3 | 5.251829885 | 0.000303 | 0.061365 | 0 | 1.3352134 | 4.8307542 | 3.198896 | 17.72684 | 47.11208 | 1.10659022 | 0.854125 | 0 | 0 | 0 | 0 | 0 |
| PTN | 6.098280921 | 0.009606 | 0.446564 | 113.88611 | 0 | 1.9323017 | 0 | 0 | 15.70403 | 3.31977065 | 0 | 0 | 0 | 0 | 1.198254 | 0 |
| CAPN6 | 21.8031266 | 4.58E-13 | 6.68E-09 | 193.80619 | 0 | 0 | 0 | 0 | 61.83461 | 0 | 0 | 0 | 0 | 0 | 0 | 0 |

Differentially expressed genes were identified using DEGseq algorithm (fold change $\geqslant 2$, $p < 0.05$).

**Supplementary Table 2.** List of genes with increased chromatin accessibility at promoter region and their expressions at stem state and differentiated state

| Gene expression linked to peaks up in TO | Gene ID | Peak Annotation | Distance to TSS | p-value | Fold Change (log2) | TO vs. Non-TO |
|---|---|---|---|---|---|---|
| Stem state | FGF10 | promoter-TSS (ENST00000264664.4) | -279 | 0.006825 | 6.49 | up |
| | ITGAX | promoter-TSS (ENST00000562522.2) | -291 | 0.011157 | 4.35 | up |
| | KLK12 | promoter-TSS (ENST00000250351.4) | -53 | 0.001547 | 2.26 | up |
| | RUNX1T1 | promoter-TSS (ENST00000614812.4) | -12 | 0.019282 | 5.10 | up |
| | ARHGAP24 | promoter-TSS (ENST00000264343.4) | -906 | 0.005496 | -1.52 | down |
| | GRAMD1B | promoter-TSS (ENST00000646146.1) | -346 | 0.009418 | -3.50 | down |
| | IPCEF1 | promoter-TSS (ENST00000367220.8) | -25 | 0.027998 | -1.88 | down |
| | RARB | promoter-TSS (ENST00000458646.1) | -76 | 0.020646 | -3.20 | down |
| | SEC16B | promoter-TSS (ENST00000308284.10) | -60 | 0.03691 | -1.12 | down |
| | | | | | | |
| Differentiated state | ADAMTS5 | promoter-TSS (ENST00000284987.5) | -899 | 0.000927 | 6.34 | up |
| | AGMO | promoter-TSS (ENST00000342526.7) | 11 | 0.015141 | 3.33 | up |
| | ATP6V0D2 | promoter-TSS (ENST00000285393.3) | 49 | 0.000745 | 5.49 | up |
| | C4orf19 | promoter-TSS (ENST00000284437.6) | 59 | 0.023027 | 1.28 | up |
| | CD69 | promoter-TSS (ENST00000228434.7) | -302 | 0.006832 | 5.20 | up |
| | COL5A2 | promoter-TSS (ENST00000374866.8) | -612 | 5.35E-07 | 3.35 | up |
| | CSGALNACT1 | promoter-TSS (ENST00000332246.10) | 66 | 0.006357 | 1.75 | up |
| | GABRP | promoter-TSS (ENST00000518525.5) | -55 | 1.28E-11 | 1.41 | up |
| | GNG4 | promoter-TSS (ENST00000484517.2) | -505 | 0.02777 | 2.29 | up |
| | IDO1 | promoter-TSS (ENST00000518237.5) | -201 | 1.34E-05 | 4.80 | up |
| | MICAL2 | promoter-TSS (ENST00000527546.5) | -478 | 6.05E-05 | 1.65 | up |
| | ODAPH | promoter-TSS (ENST00000616557.1) | -209 | 0.000544 | 3.74 | up |
| | PITPNM2 | promoter-TSS (ENST00000542749.5) | -284 | 0.000982 | 1.18 | up |
| | PTPN22 | promoter-TSS (ENST00000525799.1) | -159 | 0.03057 | 2.51 | up |
| | SATB2 | promoter-TSS (ENST00000457245.5) | -242 | 0.026141 | 1.53 | up |
| | SH2D1B | promoter-TSS (ENST00000367929.2) | 23 | 0.007382 | 3.07 | up |
| | TENM2 | promoter-TSS (ENST00000518659.5) | -385 | 1.3E-07 | 1.99 | up |
| | TNFSF11 | promoter-TSS (ENST00000358545.6) | -383 | 0.006503 | 2.01 | up |
| | BTNL8 | promoter-TSS (ENST00000410920.1) | 112 | 0.026586 | -3.69 | down |
| | CFAP58 | promoter-TSS (ENST00000369703.1) | 50 | 0.002 | -2.39 | down |
| | FABP6 | promoter-TSS (ENST00000402432.3) | -590 | 2.12E-06 | -4.35 | down |
| | KLK14 | promoter-TSS (ENST00000650543.1) | -348 | 0.016259 | -2.31 | down |
| | LYZ | promoter-TSS (ENST00000549690.1) | 56 | 0.005913 | -1.48 | down |
| | MS4A8 | promoter-TSS (ENST00000300226.6) | 100 | 0.00853 | -2.70 | down |
| | NEK10 | promoter-TSS (ENST00000383770.4) | -458, -640 | 0.041795 | -1.76 | down |
| | PACRG | promoter-TSS (ENST00000611387.1) | -900 | 0.003251 | -2.72 | down |
| | PPCDC | promoter-TSS (ENST00000563393.1) | 98 | 0.039533 | -1.66 | down |
| | RP1 | promoter-TSS (ENST00000637698.1) | 94 | 0.036107 | -1.83 | down |
| | SMIM6 | promoter-TSS (ENST00000579469.1) | -232 | 3.73E-06 | -4.29 | down |
| | TEKT4 | promoter-TSS (ENST00000295201.4) | -443 | 0.001103 | -3.05 | down |

**Supplementary Table 3.** List of genes with decreased chromatin accessibility at promoter region and their expressions at stem state and differentiation state

| Gene expression linked to peaks down in TO | Gene ID | Peak Annotation | Distance to TSS | p-value | Fold Change (log2) | TO vs. Non-TO |
|---|---|---|---|---|---|---|
| Stem state | TNS1 | promoter-TSS (ENST00000646520.1) | -33 | 0.004181 | -1.62 | down |
| | BAIAP3 | promoter-TSS (ENST00000628027.2) | -18 | 0.015623 | -2.97 | down |
| | PAMR1 | promoter-TSS (ENST00000621476.4) | -191 | 1.23E-05 | -4.34 | down |
| | NFIX | promoter-TSS (ENST00000587760.5) | -921 | 0.007613 | -1.47 | down |
| | APCDD1 | promoter-TSS (ENST00000578882.1) | -9 | 0.001803 | -2.70 | down |
| | ZFP3 | promoter-TSS (ENST00000574352.1) | 147 | 0.008388 | -1.77 | down |
| | ALOX15 | promoter-TSS (ENST00000570836.5) | -513 | 0.006552 | -5.64 | down |
| | ATP2A1 | promoter-TSS (ENST00000561547.5) | 55 | 0.021148 | -1.47 | down |
| | BMP4 | promoter-TSS (ENST00000558984.1) | -365 | 0.000296 | -2.90 | down |
| | NR4A1 | promoter-TSS (ENST00000545748.5) | -610 | 0.000262 | -2.67 | down |
| | SFTA3 | promoter-TSS (ENST00000518529.6) | -348 | 9.49E-05 | -4.93 | down |
| | NGFR | promoter-TSS (ENST00000504201.1) | -780 | 1.81E-07 | -1.64 | down |
| | NKX2-1 | promoter-TSS (ENST00000498187.6) | 69 | 0.0355 | -5.39 | down |
| | FRMD5 | promoter-TSS (ENST00000484674.5) | 317 | 0.000449 | -3.37 | down |
| | MNX1 | promoter-TSS (ENST00000480284.1) | 120 | 0.003431 | -6.58 | down |
| | COL6A2 | promoter-TSS (ENST00000454245.1) | -82 | 0.044091 | -7.49 | down |
| | HCAR1 | promoter-TSS (ENST00000432564.2) | 5 | 0.001076 | -4.79 | down |
| | WNK2 | promoter-TSS (ENST00000427277.6) | -483 | 0.014241 | -2.31 | down |
| | PHYHD1 | promoter-TSS (ENST00000421063.6) | 24 | 0.042853 | -1.11 | down |
| | C2orf74 | promoter-TSS (ENST00000420918.3) | -53 | 8.55E-06 | -2.38 | down |
| | PGGHG | promoter-TSS (ENST00000409479.5) | -406 | 8.04E-05 | -2.09 | down |
| | IFITM1 | promoter-TSS (ENST00000408968.3) | 56 | 9.8E-05 | -2.97 | down |
| | ITGB2 | promoter-TSS (ENST00000397857.5) | -139 | 0.000977 | -4.47 | down |
| | NRP1 | promoter-TSS (ENST00000395995.5) | -281 | 0.001018 | -1.15 | down |
| | PANX2 | promoter-TSS (ENST00000395842.2) | -710 | 0.043984 | -1.44 | down |
| | IGF2 | promoter-TSS (ENST00000381406.8) | -190 | 0.006076 | -1.78 | down |
| | PAQR7 | promoter-TSS (ENST00000374296.3) | -527 | 0.017417 | -1.02 | down |
| | WNT2B | promoter-TSS (ENST00000369684.4) | -42 | 0.037642 | -1.07 | down |
| | SV2A | promoter-TSS (ENST00000369146.7) | -145 | 0.010278 | -2.63 | down |
| | CIART | promoter-TSS (ENST00000369095.5) | -346 | 0.015809 | -1.48 | down |
| | LGR6 | promoter-TSS (ENST00000367278.7) | -514 | 0.012292 | -4.43 | down |
| | CCDC88B | promoter-TSS (ENST00000359902.2) | -745 | 0.011935 | -1.63 | down |
| | WNK3 | promoter-TSS (ENST00000354646.6) | -256 | 0.026961 | -3.72 | down |
| | PAK5 | promoter-TSS (ENST00000353224.9) | -91 | 0.040812 | -5.12 | down |
| | EFS | promoter-TSS (ENST00000351354.3) | -435 | 0.023909 | -1.10 | down |
| | TRPC6 | promoter-TSS (ENST00000348423.8) | -13 | 3.01E-05 | -7.75 | down |
| | NRG2 | promoter-TSS (ENST00000340391.7) | -160 | 0.000327 | -3.31 | down |
| | VWA1 | promoter-TSS (ENST00000338660.5) | -49 | 0.000741 | -1.54 | down |
| | B4GALNT4 | promoter-TSS (ENST00000329962.10) | -214 | 1.05E-05 | -1.88 | down |
| | FAIM2 | promoter-TSS (ENST00000320634.7) | -71 | 0.019572 | -4.18 | down |
| | HEG1 | promoter-TSS (ENST00000311127.8) | -646 | 0.004292 | -1.51 | down |
| | AMOT | promoter-TSS (ENST00000304758.5) | -309 | 0.01086 | -4.81 | down |
| | SIX2 | promoter-TSS (ENST00000303077.6) | -265 | 0.009051 | -2.41 | down |
| | KRT8 | promoter-TSS (ENST00000293308.11) | -720 | 2.17E-17 | -3.28 | down |
| | PLEKHH2 | promoter-TSS (ENST00000282406.8) | 95 | 0.00657 | -1.02 | down |
| | NTRK2 | promoter-TSS (ENST00000277120.7) | -537 | 0.018174 | -4.82 | down |
| | PSD2 | promoter-TSS (ENST00000274710.3) | -75 | 0.024297 | -6.13 | down |
| | CABLES1 | promoter-TSS (ENST00000256925.11) | -5 | 0.02386 | -2.02 | down |
| | ALDH3A1 | promoter-TSS (ENST00000225740.10) | 4, 14 | 1.51E-07 | -2.12 | down |
| | PPP2R2C | promoter-TSS (ENST00000506140.5) | 68 | 0.032996 | 1.26 | up |
| | TNFSF18 | promoter-TSS (ENST00000404377.3) | -143 | 0.000986 | 2.53 | up |
| | PRSS3 | promoter-TSS (ENST00000379405.3) | -173 | 0.003301 | 1.63 | up |

Continue on the next page…

| | | | | | | |
|---|---|---|---|---|---|---|
| Differentiated state | AC074143.1 | promoter-TSS (ENST00000518227.1) | 62 | 0.023879 | -1.60 | down |
| | ADGRB2 | promoter-TSS (ENST00000373658.7) | -136 | 0.019904 | -1.53 | down |
| | ALDH3A1 | promoter-TSS (ENST00000225740.10) | 4, 14 | 0.000446 | -3.45 | down |
| | ALPL | promoter-TSS (ENST00000540617.5) | -93 | 0.011294 | -2.23 | down |
| | ANKRD24 | promoter-TSS (ENST00000262970.9) | 40 | 0.021459 | -1.28 | down |
| | ANKUB1 | promoter-TSS (ENST00000446160.6) | 13 | 0.000173 | -2.39 | down |
| | APCDD1 | promoter-TSS (ENST00000578882.1) | -9 | 0.001778 | -2.84 | down |
| | BAIAP3 | promoter-TSS (ENST00000628027.2) | -18 | 0.023153 | -1.91 | down |
| | BMP8A | promoter-TSS (ENST00000331593.5) | -319 | 0.018854 | -1.70 | down |
| | C2orf74 | promoter-TSS (ENST00000420918.3) | -53 | 0.004271 | -2.28 | down |
| | CBY1 | promoter-TSS (ENST00000355830.10) | 95 | 8.97E-05 | -1.79 | down |
| | CCR10 | promoter-TSS (ENST00000591765.1) | -120 | 0.001471 | -2.98 | down |
| | COA3 | promoter-TSS (ENST00000588408.5) | 573 | 0.005453 | -1.63 | down |
| | EFS | promoter-TSS (ENST00000351354.3) | -435 | 0.011481 | -1.25 | down |
| | FAIM2 | promoter-TSS (ENST00000320634.7) | -71 | 2.78E-05 | -4.25 | down |
| | FAM183A | promoter-TSS (ENST00000335282.4) | -272 | 1.09E-05 | -3.55 | down |
| | FOXJ1 | promoter-TSS (ENST00000322957.6) | -125 | 0.003167 | -2.29 | down |
| | FRMPD2 | promoter-TSS (ENST00000305531.3) | -16 | 0.033067 | -1.93 | down |
| | GLB1L | promoter-TSS (ENST00000409640.5) | 72 | 0.000195 | -2.32 | down |
| | GPR162 | promoter-TSS (ENST00000428545.6) | -24 | 0.002601 | -3.20 | down |
| | GSTM4 | promoter-TSS (ENST00000369833.5) | -16 | 0.002384 | -1.97 | down |
| | HHEX | promoter-TSS (ENST00000282728.9) | -103 | 0.042015 | -1.91 | down |
| | ICAM4 | promoter-TSS (ENST00000393717.2) | -424 | 0.023663 | -1.20 | down |
| | IGFBP6 | promoter-TSS (ENST00000549628.1) | -414 | 0.016203 | -2.67 | down |
| | ITPKA | promoter-TSS (ENST00000260386.6) | 11 | 0.00934 | -1.62 | down |
| | KCNB1 | promoter-TSS (ENST00000371741.5) | -283 | 0.037904 | -1.51 | down |
| | KCNH3 | promoter-TSS (ENST00000257981.6) | -690 | 0.005005 | -2.28 | down |
| | KCNJ16 | promoter-TSS (ENST00000615244.4) | -167 | 0.033686 | -3.92 | down |
| | KIF18B | promoter-TSS (ENST00000587309.5) | 12 | 3.94E-10 | -4.30 | down |
| | KRT8 | promoter-TSS (ENST00000293308.11) | -720 | 0.010438 | -1.46 | down |
| | LGR6 | promoter-TSS (ENST00000367278.7) | -514 | 0.012392 | -3.85 | down |
| | LMNA | promoter-TSS (ENST00000532414.2) | 125 | 0.028175 | -1.58 | down |
| | MPPED1 | promoter-TSS (ENST00000443721.1) | -31 | 1.1E-05 | -5.53 | down |
| | NPAS1 | promoter-TSS (ENST00000449844.6) | 466 | 0.003975 | -1.94 | down |
| | NTRK2 | promoter-TSS (ENST00000277120.7) | -537 | 0.011582 | -2.62 | down |
| | NUPR1 | promoter-TSS (ENST00000395641.2) | -45 | 0.008744 | -1.99 | down |
| | PAQR5 | promoter-TSS (ENST00000563004.2) | 458 | 0.003522 | -1.70 | down |
| | PLEKHO1 | promoter-TSS (ENST00000369124.4) | -12 | 0.014104 | -1.83 | down |
| | PLIN4 | promoter-TSS (ENST00000633942.1) | -828 | 0.033122 | -1.19 | down |
| | POLR2J | promoter-TSS (ENST00000393794.4) | -121 | 0.013957 | -1.89 | down |
| | PPM1N | promoter-TSS (ENST00000401593.5) | -451 | 0.031517 | -1.45 | down |
| | PPP1R1B | promoter-TSS (ENST00000579000.5) | -127 | 0.000441 | -3.36 | down |
| | RBM24 | promoter-TSS (ENST00000425446.6) | 131 | 0.03824 | -1.65 | down |
| | RBP7 | promoter-TSS (ENST00000294435.7) | 53 | 0.003665 | -3.42 | down |
| | REC8 | promoter-TSS (ENST00000611366.4) | 56 | 0.035379 | -4.05 | down |
| | RND2 | promoter-TSS (ENST00000587250.3) | -379 | 8.98E-05 | -2.55 | down |
| | RNF157 | promoter-TSS (ENST00000319945.10) | -401 | 0.004983 | -2.61 | down |
| | SEMA4A | promoter-TSS (ENST00000368286.6) | 74 | 0.013837 | -1.09 | down |
| | SHISA8 | promoter-TSS (ENST00000621082.1) | -355 | 0.000244 | -3.49 | down |
| | SIX2 | promoter-TSS (ENST00000303077.6) | -265 | 0.000579 | -2.25 | down |
| | SMKR1 | promoter-TSS (ENST00000462322.2) | 60 | 1E-05 | -3.84 | down |
| | SOX5 | promoter-TSS (ENST00000546136.5) | -2 | 0.002097 | -3.29 | down |
| | STMN1 | promoter-TSS (ENST00000399728.5) | 9 | 0.001221 | -1.51 | down |
| | SV2A | promoter-TSS (ENST00000369146.7) | -145 | 0.015212 | -3.39 | down |
| | TEKT2 | promoter-TSS (ENST00000446354.2) | 150 | 0.000143 | -3.22 | down |
| | TFF3 | promoter-TSS (ENST00000291525.11) | -20 | 1.89E-07 | -3.70 | down |
| | TRPC6 | promoter-TSS (ENST00000348423.8) | -13 | 0.000826 | -4.68 | down |
| | TTLL6 | promoter-TSS (ENST00000393382.7) | 100 | 0.022268 | -2.15 | down |

**Supplementary Table 4.** List of genes with increased chromatin accessibility at exon region and their expressions at stem state and differentiated state

| Gene expression linked to peaks up in TO | Gene ID | Peak Annotation | Distance to TSS | p-value | Fold Change (log2) | TO vs. Non-TO |
|---|---|---|---|---|---|---|
| **Stem state** | SDCBP2 | exon (ENST00000614659.1, exon 4 of 4) | 6912 | 0.001338 | 1.73 | up |
| | TNFSF14 | exon (ENST00000599359.1, exon 2 of 5) | 161 | 0.017341 | 5.37 | up |
| | ADGRE2 | exon (ENST00000596991.6, exon 19 of 20) | 33237 | 0.029884 | 2.05 | up |
| | VNN2 | exon (ENST00000525270.5, exon 2 of 8) | 130 | 0.007287 | 6.87 | up |
| | CWH43 | exon (ENST00000513409.1, exon 6 of 16) | 11877 | 0.000112 | 2.52 | up |
| | FAM3D | exon (ENST00000482387.6, exon 17 of 17) | -75644 | 0.004619 | 4.43 | up |
| | MUC4 | exon (ENST00000463781.7, exon 2 of 25) | 21603 | 0.015398 | 5.23 | up |
| | SPINK5 | exon (ENST00000398454.5, exon 4 of 28) | 8213 | 0.000721 | 1.10 | up |
| | GCNT3 | exon (ENST00000396065.2, exon 3 of 3) | 6744 | 0.02974 | 3.72 | up |
| | PIK3AP1 | exon (ENST00000371109.3, exon 1 of 10) | 115 | 0.020077 | 1.79 | up |
| | ESR1 | exon (ENST00000338799.9, exon 1 of 9) | 901 | 0.011128 | 2.55 | up |
| | STX3 | exon (ENST00000307552.3, exon 1 of 1) | 111 | 0.009271 | 1.21 | up |
| | ACER1 | exon (ENST00000301452.4, exon 1 of 6) | 169 | 0.016538 | 4.46 | up |
| | GPRIN1 | exon (ENST00000261944.9, exon 27 of 32) | 19688 | 0.045576 | 1.04 | up |
| | HLF | exon (ENST00000376352.6, exon 18 of 18) | -98065 | 0.003667 | -2.67 | down |
| | LOX | exon (ENST00000339397.4, exon 8 of 8) | 50017 | 8.60E-05 | -3.09 | down |
| | CAVIN2 | exon (ENST00000304141.4, exon 2 of 2) | 11535 | 3.48E-05 | -1.69 | down |
| | | | | | | |
| **Differentiated state** | ADGRE2 | exon (ENST00000596991.6, exon 19 of 20) | 33237 | 0.011349 | 1.86 | up |
| | BMP3 | exon (ENST00000282701.2, exon 1 of 3) | 625 | 6.42E-08 | 2.77 | up |
| | EMP1 | exon (ENST00000256951.9, exon 5 of 5) | 7210 | 0.004608 | 1.10 | up |
| | ESR1 | exon (ENST00000338799.9, exon 1 of 9) | 901 | 0.008816 | 2.68 | up |
| | FKBP14 | exon (ENST00000222803.9, exon 4 of 4) | 13614 | 0.000662 | 1.44 | up |
| | IRAK3 | exon (ENST00000457197.2, exon 4 of 11) | 22300 | 0.000296 | 1.84 | up |
| | ISM2 | exon (ENST00000216484.6, exon 12 of 12) | -9928 | 0.00078 | 2.42 | up |
| | LAMA3 | exon (ENST00000399516.7, exon 33 of 74) | -14929 | 5.21E-09 | 2.94 | up |
| | MGAM | exon (ENST00000549489.6, exon 11 of 48) | 34593 | 0.033523 | 2.63 | up |
| | NRG1 | exon (ENST00000356819.7, exon 12 of 12) | 44474 | 0.014837 | 1.26 | up |
| | NTM | exon (ENST00000374791.7, exon 1 of 8) | 360 | 0.038128 | 3.22 | up |
| | PCDHA13 | exon (ENST00000625195.1, exon 1 of 2) | 127 | 0.030017 | 3.86 | up |
| | PPARG | exon (ENST00000643888.1, exon 7 of 9) | 54510 | 0.007546 | 1.32 | up |
| | SATB2 | exon (ENST00000428695.5, exon 1 of 7) | 207 | 0.026141 | 1.53 | up |
| | SEC24D | exon (ENST00000511481.5, exon 13 of 16) | 37072 | 0.048046 | 1.16 | up |
| | STX3 | exon (ENST00000307552.3, exon 1 of 1) | 111 | 0.003332 | 1.04 | up |
| | TNFSF14 | exon (ENST00000599359.1, exon 2 of 5) | 161 | 0.002289 | 3.62 | up |
| | DEGS2 | exon (ENST00000544450.6, exon 11 of 11) | 21019 | 0.003834 | -2.36 | down |
| | EFHC1 | exon (ENST00000360726.3, exon 3 of 3) | -16338 | 0.005452 | -1.86 | down |
| | ERICH3 | exon (ENST00000612390.4, exon 3 of 3) | 10371 | 0.040271 | -2.40 | down |
| | FANK1 | exon (ENST00000368676.8, exon 19 of 19), (ENST00000368679.8, exon 23 of 23) | 66975, 39130 | 1.78E-06 | -2.53 | down |
| | FBF1 | exon (ENST00000592193.6, exon 3 of 28) | 4524 | 0.004857 | -1.49 | down |
| | IL22RA1 | exon (ENST00000327535.5, exon 4 of 7) | -18526 | 0.003463 | -1.83 | down |
| | LCA5L | exon (ENST00000485895.6, exon 5 of 5) | 5677 | 0.011146 | -1.79 | down |
| | MYLK3 | exon (ENST00000394809.8, exon 1 of 13) | 387 | 0.006472 | -2.60 | down |
| | NCAPH | exon (ENST00000427946.5, exon 7 of 17) | 17568 | 0.011858 | -2.33 | down |
| | NTRK2 | exon (ENST00000376208.5, exon 14 of 14) | 203993 | 0.011582 | -2.62 | down |
| | SYNE3 | exon (ENST00000557275.5, exon 17 of 17) | 41904 | 0.04791 | -1.67 | down |
| | TCTEX1D1 | exon (ENST00000371037.8, exon 22 of 25) | -13116 | 0.030144 | -2.20 | down |

**Supplementary Table 5.** List of genes with decreased chromatin accessibility at exon region and their expressions at stem state and differentiated state

| Gene expression linked to peaks down in TO | Gene ID | Peak Annotation | Distance to TSS | p-value | Fold Change (log2) | TO vs. Non-TO |
|---|---|---|---|---|---|---|
| **Stem state** | GNAT1 | exon (ENST00000232461.7, exon 8 of 9) | 3172 | 0.04349328 | -4.27 | down |
| | BMP4 | exon (ENST00000245451.8, exon 1 of 4) | 257 | 2.75708E-05 | -2.72 | down |
| | PRPH | exon (ENST00000257860.8, exon 6 of 9) | 3849 | 0.013755449 | -4.61 | down |
| | WNT10A | exon (ENST00000258411.7, exon 1 of 4) | 118 | 0.017549088 | -1.05 | down |
| | PHYHD1 | exon (ENST00000259324.5, exon 4 of 4) | -3444 | 0.002430617 | -1.15 | down |
| | IGFBP4 | exon (ENST00000269593.4, exon 1 of 4) | 343 | 0.013647231 | -1.53 | down |
| | WNT3A | exon (ENST00000284523.1, exon 1 of 4) | 144 | 0.000203853 | -2.11 | down |
| | SOSTDC1 | exon (ENST00000307068.4, exon 1 of 2) | 128 | 0.002798447 | -4.11 | down |
| | CCDC8 | exon (ENST00000307522.4, exon 1 of 1) | 254 | 0.000165404 | -3.15 | down |
| | GGT5 | exon (ENST00000327365.8, exon 1 of 12) | 498 | 1.70261E-12 | -4.43 | down |
| | METTL7A | exon (ENST00000332160.4, exon 1 of 2) | 162 | 0.000470109 | -1.68 | down |
| | CKB | exon (ENST00000348956.6, exon 1 of 8) | 148 | 0.003734738 | -1.60 | down |
| | SUSD2 | exon (ENST00000358321.3, exon 1 of 15) | 181 | 1.98678E-07 | -3.35 | down |
| | NRG2 | exon (ENST00000361474.5, exon 1 of 10) | 332 | 0.00075442 | -2.72 | down |
| | TREX2 | exon (ENST00000370211.8, exon 8 of 10) | -7882 | 0.025254889 | -1.24 | down |
| | LDOC1 | exon (ENST00000370526.3, exon 1 of 1) | 266 | 1.04493E-06 | -4.72 | down |
| | SMOC1 | exon (ENST00000381280.4, exon 1 of 12) | 231 | 2.2141E-06 | -1.69 | down |
| | RUNX3 | exon (ENST00000399916.5, exon 2 of 6) | 138 | 0.028468019 | -2.66 | down |
| | RBM38 | exon (ENST00000417346.1, exon 1 of 2) | 386 | 0.000912418 | -1.17 | down |
| | HCAR1 | exon (ENST00000432564.2, exon 1 of 1) | 4545 | 0.003119762 | -2.92 | down |
| | PROB1 | exon (ENST00000434752.3, exon 1 of 1) | 1529 | 0.004149771 | -1.09 | down |
| | RNF165 | exon (ENST00000588679.1, exon 1 of 1) | 271 | 3.08181E-10 | -2.52 | down |
| | MYO15B | exon (ENST00000610510.4, exon 1 of 63) | 1077 | 0.026822784 | -1.20 | down |
| | TPRG1 | exon (ENST00000617246.4, exon 11 of 11) | -65461 | 0.000119316 | -1.21 | down |
| | PRODH | exon (ENST00000638240.1, exon 6 of 6) | -10702 | 0.035269387 | -2.84 | down |

Continue on the next page…

| | | | | | | |
|---|---|---|---|---|---|---|
| **Differentiated state** | DOK7 | exon (ENST00000340083.5, exon 3 of 7) | 10172 | 0.000405461 | -1.37 | down |
| | DOK7 | exon (ENST00000340083.5, exon 7 of 7) | 9813 | 0.000405461 | -1.37 | down |
| | ESPN | exon (ENST00000636330.1, exon 11 of 11) | 7221 | 0.001509439 | -1.52 | down |
| | ESPN | exon (ENST00000636330.1, exon 8 of 11) | 689 | 0.001509439 | -1.52 | down |
| | DMRT3 | exon (ENST00000190165.2, exon 1 of 2) | 114 | 0.008928564 | -3.33 | down |
| | MAMSTR | exon (ENST00000222145.8, exon 5 of 12) | -9517 | 0.014971701 | -1.87 | down |
| | LGR6 | exon (ENST00000255432.11, exon 1 of 18) | 117 | 0.012391622 | -3.85 | down |
| | PRPH | exon (ENST00000257860.8, exon 6 of 9) | 3849 | 0.038094502 | -2.18 | down |
| | PIMREG | exon (ENST00000262483.12, exon 20 of 20) | 10881 | 8.54161E-09 | -4.52 | down |
| | UBE2S | exon (ENST00000264552.13, exon 4 of 4) | 7192 | 0.000996521 | -2.64 | down |
| | RCN3 | exon (ENST00000270645.7, exon 1 of 7) | 267 | 0.015963982 | -1.93 | down |
| | WNT3A | exon (ENST00000284523.1, exon 1 of 4) | 144 | 0.007349871 | -2.47 | down |
| | HRASLS5 | exon (ENST00000301790.4, exon 1 of 6) | 186 | 0.008114229 | -2.35 | down |
| | CCDC8 | exon (ENST00000307522.4, exon 1 of 1) | 254 | 0.021311823 | -2.13 | down |
| | DEF6 | exon (ENST00000316637.6, exon 7 of 11) | 20520 | 0.002836985 | -1.19 | down |
| | PSAPL1 | exon (ENST00000319098.6, exon 1 of 1) | 4304 | 0.000181007 | -3.11 | down |
| | GPR62 | exon (ENST00000322241.5, exon 1 of 1) | 1023 | 0.015472082 | -4.30 | down |
| | C11orf65 | exon (ENST00000323468.9, exon 8 of 8) | -5329 | 0.027419126 | -1.30 | down |
| | HMG20B | exon (ENST00000333651.10, exon 8 of 10) | 4002 | 0.047601065 | -1.00 | down |
| | CAPS | exon (ENST00000339485.3, exon 2 of 2) | -1510 | 1.26422E-05 | -4.04 | down |
| | IFT27 | exon (ENST00000340630.9, exon 5 of 7) | 12156 | 0.000165931 | -2.66 | down |
| | CKB | exon (ENST00000348956.6, exon 1 of 8) | 148 | 1.79983E-05 | -2.56 | down |
| | PLEKHA4 | exon (ENST00000355496.9, exon 8 of 17) | 9515 | 0.004077529 | -1.35 | down |
| | ITPRIPL1 | exon (ENST00000361124.5, exon 1 of 1) | 4084 | 2.18949E-05 | -2.68 | down |
| | ABCA4 | exon (ENST00000370225.3, exon 33 of 50) | 41336 | 0.037652708 | -1.21 | down |
| | LDOC1 | exon (ENST00000370526.3, exon 1 of 1) | 266 | 0.00679574 | -2.83 | down |
| | CFP | exon (ENST00000376983.7, exon 5 of 7) | -7695 | 0.006550587 | -2.66 | down |
| | ZBTB48 | exon (ENST00000377674.8, exon 8 of 11) | 8052 | 0.013524327 | -1.59 | down |
| | SERP2 | exon (ENST00000379179.7, exon 1 of 3) | 106 | 0.03630176 | -1.72 | down |
| | SPEF1 | exon (ENST00000379756.3, exon 7 of 7) | 3460 | 0.000514229 | -3.48 | down |
| | SLC47A1 | exon (ENST00000395585.5, exon 1 of 19) | 116 | 0.048438318 | -3.72 | down |
| | RBM38 | exon (ENST00000417346.1, exon 1 of 2) | 386 | 0.002178803 | -1.53 | down |
| | ISOC2 | exon (ENST00000438389.6, exon 5 of 5) | 8401 | 0.002990006 | -2.68 | down |
| | MMP23B | exon (ENST00000505820.6, exon 20 of 20) | -1643 | 0.02507483 | -4.87 | down |
| | NDUFA9 | exon (ENST00000527518.1, exon 4 of 4) | 16196 | 0.035511362 | -1.61 | down |
| | MYO15B | exon (ENST00000610510.4, exon 1 of 63) | 1077 | 0.010998191 | -1.93 | down |
| | PRR5 | exon (ENST00000617066.4, exon 1 of 8) | 128 | 0.048949757 | -1.49 | down |
| | TPRG1 | exon (ENST00000617246.4, exon 11 of 11) | -65461 | 0.02411273 | -1.53 | down |
| | PRODH | exon (ENST00000638240.1, exon 6 of 6) | -10702 | 0.040648883 | -2.72 | down |
| | BMP4 | exon (ENST00000245451.8, exon 1 of 4) | 257 | 0.004487366 | 1.89 | up |
| | EFHD1 | exon (ENST00000264059.7, exon 1 of 4) | 614 | 0.022659204 | 3.07 | up |
| | GAL | exon (ENST00000265643.3, exon 1 of 6) | 225 | 0.039827589 | 4.59 | up |
| | MYOM3 | exon (ENST00000270800.1, exon 7 of 7) | -8239 | 7.24585E-05 | 2.42 | up |
| | CXCL8 | exon (ENST00000307407.7, exon 1 of 4) | 131 | 0.001728359 | 3.12 | up |
| | SPOCK2 | exon (ENST00000317376.8, exon 2 of 12) | 228 | 1.51222E-15 | 5.48 | up |
| | SLC39A14 | exon (ENST00000356766.10, exon 23 of 23) | -11881 | 0.000272043 | 1.87 | up |
| | DKK3 | exon (ENST00000396505.6, exon 2 of 8) | 625 | 3.17259E-07 | 2.19 | up |
| | NAT8L | exon (ENST00000423729.2, exon 3 of 3) | 4783 | 0.002674438 | 5.83 | up |
| | CPAMD8 | exon (ENST00000443236.5, exon 1 of 42) | 242 | 0.000416259 | 1.96 | up |
| | BIRC3 | exon (ENST00000615299.4, exon 3 of 10) | 7144 | 0.000404694 | 2.52 | up |

**Supplementary Table 6.** List of genes with decreased chromatin accessibility at intron region and their expressions at differentiated state

| Gene expression linked to peaks down in TO | Gene ID | Peak Annotation | Distance to TSS | p-value | Fold Change (log2) | TO vs. Non-TO |
|---|---|---|---|---|---|---|
| Differentiated state | FOXP1 | intron (ENST00000475937.5, intron 10 of 19) | 31578 | 0.039143 | -1.61 | down |
| | FOXP1 | intron (ENST00000475937.5, intron 11 of 19) | 57529 | | | down |
| | FOXP1 | intron (ENST00000475937.5, intron 2 of 19) | 55389 | | | down |
| | FOXP1 | intron (ENST00000475937.5, intron 2 of 19) | 59619 | | | down |
| | FOXP1 | intron (ENST00000475937.5, intron 5 of 19) | 2804 | | | down |
| | FOXP1 | intron (ENST00000475937.5, intron 5 of 19) | 9227 | | | down |
| | FOXP1 | intron (ENST00000647725.1, intron 6 of 25) | -13861 | | | down |
| | ZBTB7C | intron (ENST00000590800.5, intron 1 of 4) | 25014 | 0.045913 | -1.28 | down |
| | ZBTB7C | intron (ENST00000590800.5, intron 3 of 4) | 63961 | | | down |
| | ZBTB7C | intron (ENST00000590800.5, intron 3 of 4) | 3689 | | | down |
| | ZBTB7C | intron (ENST00000590800.5, intron 3 of 4) | -2207 | | | down |
| | ZBTB7C | intron (ENST00000590800.5, intron 3 of 4) | 17744 | | | down |
| | LGR6 | intron (ENST00000367278.7, intron 1 of 17) | -3719 | 0.0123916 | -3.85 | down |
| | LGR6 | intron (ENST00000367278.7, intron 3 of 17) | 15253 | | | down |
| | LGR6 | intron (ENST00000439764.2, intron 1 of 15) | 8298 | | | down |
| | LGR6 | intron (ENST00000439764.2, intron 1 of 15) | 399 | | | down |
| | IL2RB | intron (ENST00000216223.9, intron 1 of 9) | 4082 | 0.0232101 | -3.29 | down |
| | IL2RB | intron (ENST00000216223.9, intron 2 of 9) | 6112 | | | down |
| | IL2RB | intron (ENST00000216223.9, intron 9 of 9) | 18308 | | | down |
| | PRODH | intron (ENST00000334029.6, intron 1 of 13) | 1866 | 0.0406489 | -2.72 | down |
| | PRODH | intron (ENST00000334029.6, intron 2 of 13) | 6505 | | | down |
| | PRODH | intron (ENST00000640084.1, intron 2 of 3) | -2236 | | | down |
| | GRM4 | intron (ENST00000374177.7, intron 1 of 8) | 760 | 0.0106985 | -2.27 | down |
| | GRM4 | intron (ENST00000374177.7, intron 1 of 8) | -4412 | | | down |
| | GRM4 | intron (ENST00000538487.6, intron 1 of 10) | 643 | | | down |
| | RPL37A | intron (ENST00000446558.5, intron 3 of 3) | 41227 | 0.0014269 | -2.25 | down |
| | RPL37A | intron (ENST00000446558.5, intron 3 of 3) | 52776 | | | down |
| | RPL37A | intron (ENST00000446558.5, intron 3 of 3) | 34741 | | | down |
| | SERPINF1 | intron (ENST00000254722.8, intron 3 of 7) | 8366 | 0.0032762 | -1.65 | down |
| | SERPINF1 | intron (ENST00000305513.11, intron 5 of 10) | 31351 | | | down |
| | TRPV4 | intron (ENST00000261740.6, intron 1 of 15) | -6104 | 0.022084 | -1.12 | down |
| | TRPV4 | intron (ENST00000261740.6, intron 1 of 15) | -4403 | | | down |
| | KRT8 | intron (ENST00000293308.11, intron 1 of 8) | 1519 | 0.010438 | -1.46 | down |
| | KRT8 | intron (ENST00000552551.5, intron 2 of 8) | 2845 | | | down |
| | CYP2S1 | intron (ENST00000310054.8, intron 1 of 8) | 495 | 0.0072782 | -1.34 | down |
| | CYP2S1 | intron (ENST00000310054.8, intron 2 of 8) | 2754 | | | down |
| | ELN | intron (ENST00000320492.11, intron 4 of 28) | 11549 | 0.0008747 | -3.69 | down |
| | ELN | intron (ENST00000414324.5, intron 1 of 30) | 4279 | | | down |
| | NTRK2 | intron (ENST00000323115.8, intron 11 of 16) | 181509 | 0.0115815 | -2.62 | down |
| | NTRK2 | intron (ENST00000395882.5, intron 6 of 12) | 41203 | | | down |
| | PLA2G4E | intron (ENST00000399518.3, intron 1 of 19) | 15962 | 0.0028415 | -2.39 | down |
| | PLA2G4E | intron (ENST00000399518.3, intron 1 of 19) | 19624 | | | down |
| | UMODL1 | intron (ENST00000400424.6, intron 1 of 22) | 1292 | 0.0143468 | -2.40 | down |
| | UMODL1 | intron (ENST00000400424.6, intron 2 of 22) | 7841 | | | down |
| | MRVI1 | intron (ENST00000541483.5, intron 1 of 19) | -2547 | 0.0013676 | -2.29 | down |
| | MRVI1 | intron (ENST00000541483.5, intron 1 of 19) | -7299 | | | down |
| | FAIM2 | intron (ENST00000550890.5, intron 4 of 10) | 5835 | 2.782E-05 | -4.25 | down |
| | FAIM2 | intron (ENST00000550890.5, intron 9 of 10) | 13946 | | | down |
| | DMRT3 | intron (ENST00000569227.1, intron 3 of 4) | -63286 | 0.0089286 | -3.33 | down |
| | DMRT3 | intron (ENST00000569227.1, intron 4 of 4) | -16794 | | | down |
| | ELFN2 | intron (ENST00000613079.4, intron 1 of 2) | 7579 | 0.0341975 | -1.00 | down |
| | ELFN2 | intron (ENST00000613079.4, intron 1 of 2) | 660 | | | down |
| | STPG1 | intron (ENST00000003583.12, intron 3 of 7) | 33895 | 0.0009024 | -1.14 | down |
| | GALK1 | intron (ENST00000200181.7, intron 26 of 39) | 17176 | 0.0351854 | -1.82 | down |
| | SLC22A4 | intron (ENST00000200652.3, intron 4 of 9) | 28033 | 0.0237632 | -1.31 | down |
| | FOXN1 | intron (ENST00000226247.2, intron 3 of 7) | 4134 | 1.679E-05 | -3.37 | down |
| | RPL10A | intron (ENST00000229769.2, intron 8 of 9) | -6987 | 7.965E-05 | -1.42 | down |

Continue on the next page…

| | | | | | | | |
|---|---|---|---|---|---|---|---|
| **Differentiated state** | MAPRE3 | intron (ENST00000233121.6, intron 1 of 6) | 699 | 0.0030833 | -1.12 | down |
| | QTRT1 | intron (ENST00000250237.9, intron 3 of 9) | 5310 | 0.0270079 | -1.25 | down |
| | EEF1AKMT1 | intron (ENST00000255305.10, intron 9 of 22) | -40441 | 0.0086936 | -1.32 | down |
| | HRASLS2 | intron (ENST00000255695.1, intron 1 of 3) | 2568 | 1.667E-06 | -3.08 | down |
| | KCNH3 | intron (ENST00000257981.6, intron 7 of 14) | 8919 | 0.0050047 | -2.28 | down |
| | PSMB7 | intron (ENST00000259457.7, intron 5 of 7) | 15632 | 0.0419781 | -1.39 | down |
| | GMPR | intron (ENST00000259727.4, intron 1 of 8) | 4771 | 0.0227813 | -1.04 | down |
| | CYSTM1 | intron (ENST00000261811.5, intron 1 of 2) | 7274 | 0.0008925 | -1.30 | down |
| | TRMU | intron (ENST00000262738.7, intron 15 of 34) | 55388 | 0.0371333 | -1.63 | down |
| | RAB36 | intron (ENST00000263116.6, intron 2 of 10) | 2464 | 0.0017616 | -2.26 | down |
| | CLDN5 | intron (ENST00000263201.5, intron 13 of 18) | 13385 | 0.0012544 | -3.74 | down |
| | CALR3 | intron (ENST00000269881.7, intron 3 of 8) | 7301 | 0.0486552 | -4.91 | down |
| | WDR66 | intron (ENST00000288912.8, intron 8 of 21) | 31493 | 2.801E-06 | -1.57 | down |
| | PPP1R16A | intron (ENST00000292539.8, intron 1 of 10) | -2063 | 0.0003827 | -2.08 | down |
| | POC1A | intron (ENST00000296484.6, intron 9 of 10) | 47230 | 0.0002618 | -2.62 | down |
| | MAP1A | intron (ENST00000300231.5, intron 1 of 5) | 770 | 0.0182915 | -2.34 | down |
| | TKFC | intron (ENST00000301764.11, intron 5 of 26) | -6962 | 0.0004774 | -1.30 | down |
| | SPATA24 | intron (ENST00000302060.9, intron 7 of 7) | -11275 | 0.0045507 | -1.79 | down |
| | ABR | intron (ENST00000302538.9, intron 2 of 22) | -2175 | 0.0099194 | -2.78 | down |
| | ASPSCR1 | intron (ENST00000306739.8, intron 4 of 15) | 14172 | 0.0305452 | -1.66 | down |
| | SCNN1B | intron (ENST00000307331.9, intron 2 of 13) | 20841 | 0.010799 | -1.48 | down |
| | LKAAEAR1 | intron (ENST00000308906.6, intron 1 of 1) | 604 | 6.696E-06 | -4.77 | down |
| | SOBP | intron (ENST00000317357.9, intron 1 of 6) | 2860 | 0.024268 | -1.88 | down |
| | HYAL1 | intron (ENST00000320295.12, intron 1 of 5) | 2096 | 0.028551 | -1.74 | down |
| | CCDC34 | intron (ENST00000328697.10, intron 1 of 5) | 5598 | 5.178E-05 | -1.68 | down |
| | BMP8A | intron (ENST00000331593.5, intron 1 of 6) | 9538 | 0.0188536 | -1.70 | down |
| | BRAT1 | intron (ENST00000340611.8, intron 6 of 13) | 13465 | 0.0249898 | -1.27 | down |
| | CCDC30 | intron (ENST00000340612.5, intron 1 of 20) | 4072 | 0.0492074 | -1.47 | down |
| | SERPINB4 | intron (ENST00000341074.9, intron 4 of 7) | 2800 | 0.0088926 | -1.18 | down |
| | SSBP4 | intron (ENST00000348495.10, intron 4 of 16) | -4182 | 0.0004209 | -2.04 | down |
| | SLC47A2 | intron (ENST00000350657.9, intron 8 of 17) | 9434 | 0.0218463 | -1.22 | down |
| | SPTBN4 | intron (ENST00000352632.7, intron 3 of 35) | 20826 | 0.0025147 | -2.55 | down |
| | NR2E3 | intron (ENST00000355327.7, intron 8 of 17) | -115506 | 0.0140028 | -2.69 | down |
| | BOC | intron (ENST00000355385.7, intron 4 of 19) | 47646 | 0.001236 | -1.28 | down |
| | DTNBP1 | intron (ENST00000355917.7, intron 6 of 8) | 8179 | 0.034845 | -1.42 | down |
| | OSCP1 | intron (ENST00000356637.9, intron 1 of 10) | 1616 | 3.272E-05 | -2.65 | down |
| | ASTN2 | intron (ENST00000361209.6, intron 16 of 21) | 45326 | 0.0115199 | -1.17 | down |
| | UBE2T | intron (ENST00000367278.7, intron 6 of 17) | 49495 | 1.795E-05 | -1.46 | down |
| | MYB | intron (ENST00000367814.8, intron 1 of 14) | 4036 | 0.0162834 | -1.89 | down |
| | SEMA4A | intron (ENST00000368284.5, intron 6 of 12) | 6945 | 0.0138373 | -1.09 | down |
| | PMVK | intron (ENST00000368467.3, intron 2 of 4) | 4697 | 0.0062004 | -1.86 | down |
| | PSMD4 | intron (ENST00000368884.7, intron 1 of 9) | 4778 | 0.0171525 | -1.47 | down |
| | BEND5 | intron (ENST00000371833.3, intron 4 of 5) | 35188 | 0.042724 | -2.14 | down |
| | CEL | intron (ENST00000372080.6, intron 7 of 10) | 5709 | 2.152E-05 | -1.49 | down |
| | RSPH9 | intron (ENST00000372165.8, intron 1 of 5) | 352 | 4.689E-05 | -3.68 | down |
| | CCDC24 | intron (ENST00000372318.7, intron 5 of 8) | 2645 | 0.0007492 | -2.10 | down |
| | ANGPTL2 | intron (ENST00000373425.7, intron 1 of 4) | 10000 | 0.0006579 | -4.14 | down |
| | ADGRB2 | intron (ENST00000373655.6, intron 1 of 32) | 975 | 0.019904 | -1.53 | down |
| | PIGU | intron (ENST00000374820.6, intron 5 of 10) | 52376 | 0.0239794 | -1.62 | down |
| | CROCC | intron (ENST00000375541.9, intron 3 of 36) | 3667 | 0.0005887 | -2.04 | down |

Continue on the next page…

| | | | | | | |
|---|---|---|---|---|---|---|
| **Differentiated state** | AQP7 | intron (ENST00000377425.8, intron 2 of 6) | 2743 | 0.0079073 | -2.17 | down |
| | FOLR1 | intron (ENST00000378140.3, intron 2 of 2) | -8258 | 0.0279488 | -1.69 | down |
| | NWD1 | intron (ENST00000379808.7, intron 1 of 16) | 2986 | 0.0212596 | -2.32 | down |
| | CCDC57 | intron (ENST00000392347.5, intron 14 of 16) | 18421 | 0.000774 | -1.81 | down |
| | ANKRD39 | intron (ENST00000393537.4, intron 1 of 3) | 2914 | 0.0448068 | -1.52 | down |
| | RAB17 | intron (ENST00000409822.1, intron 1 of 4) | 8317 | 0.0391141 | -1.23 | down |
| | CDCA7 | intron (ENST00000410019.3, intron 1 of 7) | 597 | 0.0108253 | -3.76 | down |
| | FBXO15 | intron (ENST00000419743.6, intron 1 of 9) | 5908 | 0.000598 | -2.90 | down |
| | PIMREG | intron (ENST00000421306.7, intron 10 of 18) | 26985 | 8.542E-09 | -4.52 | down |
| | NT5C3B | intron (ENST00000435506.6, intron 1 of 8) | 162 | 0.0273074 | -1.60 | down |
| | ZNF487 | intron (ENST00000437590.2, intron 1 of 3) | 8320 | 1.418E-05 | -1.72 | down |
| | CYHR1 | intron (ENST00000438911.6, intron 2 of 4) | 6464 | 0.0452385 | -1.15 | down |
| | MPPED1 | intron (ENST00000443721.1, intron 1 of 6) | 12718 | 1.097E-05 | -5.53 | down |
| | IGFBP5 | intron (ENST00000447289.1, intron 1 of 3) | -15507 | 0.0188297 | -2.64 | down |
| | ATP5MF | intron (ENST00000449683.5, intron 1 of 3) | 5310 | 0.0073866 | -2.13 | down |
| | FAM166B | intron (ENST00000455600.1, intron 2 of 11) | 9869 | 0.0006031 | -2.90 | down |
| | FAM178B | intron (ENST00000490605.2, intron 12 of 16) | -19015 | 0.0033887 | -2.95 | down |
| | TXNRD2 | intron (ENST00000491939.6, intron 7 of 11) | 20186 | 0.0295864 | -1.48 | down |
| | EFNA3 | intron (ENST00000505139.1, intron 1 of 4) | -5374 | 0.0227855 | -1.89 | down |
| | TOP1MT | intron (ENST00000519148.5, intron 1 of 13) | 4700 | 0.0276524 | -1.06 | down |
| | NAALADL1 | intron (ENST00000526799.5, intron 2 of 7) | 549 | 5.671E-05 | -2.91 | down |
| | NR1H3 | intron (ENST00000529444.5, intron 5 of 8) | -4854 | 0.0012189 | -2.04 | down |
| | NPIPB6 | intron (ENST00000532254.1, intron 1 of 6) | 860 | 0.0059014 | -2.88 | down |
| | ANKRD65 | intron (ENST00000537107.5, intron 2 of 3) | 379 | 9.626E-05 | -2.45 | down |
| | KIF6 | intron (ENST00000538893.5, intron 12 of 21) | -23482 | 0.0011879 | -2.48 | down |
| | CNBD2 | intron (ENST00000538900.1, intron 7 of 10) | 24426 | 0.0138565 | -3.70 | down |
| | PTMS | intron (ENST00000539187.5, intron 1 of 7) | -2091 | 0.0014826 | -2.12 | down |
| | FAM186A | intron (ENST00000543111.5, intron 4 of 7) | 1621 | 0.0488133 | -2.15 | down |
| | SLC27A2 | intron (ENST00000544960.1, intron 1 of 10) | 2299 | 0.0456375 | -1.71 | down |
| | MESP1 | intron (ENST00000560294.5, intron 13 of 16) | 15970 | 0.0211654 | -3.07 | down |
| | LRRC49 | intron (ENST00000560369.5, intron 6 of 15) | 16913 | 0.0001149 | -1.74 | down |
| | LRRC36 | intron (ENST00000563189.5, intron 2 of 10) | 11863 | 0.0005613 | -3.32 | down |
| | SCX | intron (ENST00000569669.5, intron 3 of 15) | 6560 | 0.0073543 | -2.30 | down |
| | PKMYT1 | intron (ENST00000573944.5, intron 2 of 8) | 2557 | 4.303E-17 | -4.61 | down |
| | ZNF232 | intron (ENST00000574788.5, intron 4 of 37) | 164 | 0.0001396 | -1.41 | down |
| | CLUAP1 | intron (ENST00000576634.5, intron 5 of 11) | 4541 | 2.443E-05 | -1.83 | down |
| | MBD3 | intron (ENST00000585937.1, intron 2 of 6) | 2798 | 0.0107518 | -2.23 | down |
| | KCNJ16 | intron (ENST00000586462.1, intron 1 of 1) | 953 | 0.033686 | -3.92 | down |
| | TMEM161A | intron (ENST00000587583.6, intron 11 of 11) | 17815 | 0.0475359 | -1.21 | down |
| | FOXJ1 | intron (ENST00000590137.1, intron 6 of 7) | -6687 | 0.0031671 | -2.29 | down |
| | PRKCSH | intron (ENST00000591462.5, intron 10 of 18) | 10795 | 0.0499495 | -1.34 | down |
| | SPC24 | intron (ENST00000592540.5, intron 1 of 4) | 2678 | 1.877E-08 | -4.10 | down |
| | RFX2 | intron (ENST00000592546.5, intron 5 of 16) | 26743 | 0.0018349 | -1.92 | down |
| | KIF18B | intron (ENST00000593135.5, intron 1 of 15) | -4261 | 3.942E-10 | -4.30 | down |
| | PEX11G | intron (ENST00000593942.5, intron 1 of 6) | 2646 | 0.0005022 | -2.78 | down |
| | BCAT2 | intron (ENST00000599157.5, intron 4 of 7) | -8763 | 0.0007964 | -1.18 | down |
| | IL24 | intron (ENST00000611909.4, intron 2 of 4) | 1290 | 0.0081832 | -2.34 | down |
| | RAB44 | intron (ENST00000612677.5, intron 4 of 13) | 18839 | 0.0027756 | -2.84 | down |
| | SCARF2 | intron (ENST00000622235.4, intron 1 of 10) | 409 | 0.0004136 | -2.43 | down |
| | EFHC1 | intron (ENST00000636379.1, intron 2 of 9) | 3036 | 0.0054518 | -1.86 | down |
| | GAMT | intron (ENST00000640762.1, intron 1 of 5) | 493 | 0.0420347 | -1.58 | down |
| | TPRG1 | intron (ENST00000640853.1, intron 8 of 10) | -92361 | 0.0241127 | -1.53 | down |
| | DOK7 | intron (ENST00000643608.1, intron 2 of 7) | 7339 | 0.0004055 | -1.37 | down |

Supplementary table 2-6, Fisher's exact tests were applied to identify the significance.
