# Supplementary material for: Malfunction of airway basal stem cells plays a crucial role in pathophysiology of tracheobronchopathia osteoplastica
Source: Nat Commun. 2022 Mar 14;13:1309. doi: 10.1038/s41467-022-28903-7 (PMC8921516; doi:10.1038/s41467-022-28903-7)
Supplement: Supplementary file 3 — Reporting Summary [file 41467_2022_28903_MOESM3_ESM.pdf]

nature research

Corresponding author(s): Yue Hong, Tao Ren

Last updated by author(s): Jan 31, 2022

Reporting Summary

Nature Research wishes to improve the reproducibility of the work that we publish. This form provides structure for consistency and transparency in reporting. For further information on Nature Research policies, see our Editorial Policies and the Editorial Policy Checklist.

Statistics

For all statistical analyses, confirm that the following items are present in the figure legend, table legend, main text, or Methods section.

n/a	Confirmed	
☐	☒	The exact sample size (*n*) for each experimental group/condition, given as a discrete number and unit of measurement
☐	☒	A statement on whether measurements were taken from distinct samples or whether the same sample was measured repeatedly
☐	☒	The statistical test(s) used AND whether they are one- or two-sided<br>*Only common tests should be described solely by name; describe more complex techniques in the Methods section.*
☒	☐	A description of all covariates tested
☒	☐	A description of any assumptions or corrections, such as tests of normality and adjustment for multiple comparisons
☐	☒	A full description of the statistical parameters including central tendency (e.g. means) or other basic estimates (e.g. regression coefficient) AND variation (e.g. standard deviation) or associated estimates of uncertainty (e.g. confidence intervals)
☐	☒	For null hypothesis testing, the test statistic (e.g. *F*, *t*, *r*) with confidence intervals, effect sizes, degrees of freedom and *P* value noted<br>*Give P values as exact values whenever suitable.*
☒	☐	For Bayesian analysis, information on the choice of priors and Markov chain Monte Carlo settings
☒	☐	For hierarchical and complex designs, identification of the appropriate level for tests and full reporting of outcomes
☐	☒	Estimates of effect sizes (e.g. Cohen's *d*, Pearson's *r*), indicating how they were calculated

Our web collection on statistics for biologists contains articles on many of the points above.

Software and code

Policy information about availability of computer code

Data collection	For microscopy data collection, Olympus cellSens Imaging Software Standard, ZEN 2.6 lite and Leica Application Suite X Version 1.0 were used.<br>For real-time PCR data collection, QuantStudio 5 System was used.
Data analysis	For image analysis, Fiji (version 2.1.0/1.53c), HALO v3.0.311.293 were used.<br>For sequencing data processing and analysis, STAR v2.7.9a, HTSeq v0.6.0, DESeq2 v1.34.0, GSEA v4.1.0, BWA-MEM v2.2.1, MACS2 v2.2.7.1, deepTools 3.5.0, UpSetR v1.0.3, Bedtools v2.30.0, Bedtools multicov v2.30.0, HOMER v4.11, IGV 2.8.6 were used.<br>For statistical analysis, Microsoft Excel, GraphPad Prism 9 were used.

For manuscripts utilizing custom algorithms or software that are central to the research but not yet described in published literature, software must be made available to editors and reviewers. We strongly encourage code deposition in a community repository (e.g. GitHub). See the Nature Research guidelines for submitting code & software for further information.

Data

Policy information about availability of data

All manuscripts must include a data availability statement. This statement should provide the following information, where applicable:

- Accession codes, unique identifiers, or web links for publicly available datasets
- A list of figures that have associated raw data
- A description of any restrictions on data availability

RNA-Seq and ATAC-Seq data have been deposited and are openly available at the NCBI's Gene Expression Omnibus database under accession code GSE153390 (https://www.ncbi.nlm.nih.gov/geo/query/acc.cgi?acc=GSE153390). All other data supporting the findings of this study are available within this article and its Supplementary Information files and Source Data file. A reporting summary linked to this article is available as a Supplementary Information file. Source data are

provided with this paper.

Field-specific reporting

Please select the one below that is the best fit for your research. If you are not sure, read the appropriate sections before making your selection.

- [x] Life sciences
- [ ] Behavioural & social sciences
- [ ] Ecological, evolutionary & environmental sciences

For a reference copy of the document with all sections, see nature.com/documents/nr-reporting-summary-flat.pdf

Life sciences study design

All studies must disclose on these points even when the disclosure is negative.

Sample size	Tracheobronchopathia osteochondroplastica is an under-detected airway disease. Due to overlapping symptomology and lack of sufficient awareness, the condition is often missed in clinic. Most patients were identified incidentally during intubation, CT and bronchoscopy for other indications. We aimed to collect all eligible cases that could be identified during the period of this study.
Data exclusions	Five cases were excluded from the study, because of comorbidity (i.e. three accompanied with lung cancer, one accompanied with Chondritis and another one with Sarcoidosis and Bronchiectasis).
Replication	All experimental findings were verified by replication and/or repeated measurements. All attempts to reproduce data were successful. The number of replicates is defined in figure legends.
Randomization	Samples were divided into non-disease and disease groups based on patients' diagnosis.
Blinding	Investigators were blinded from clinical details (e.g. stage, type, history, etc.) until cell characterization and differentiation assessment had been completed.

Reporting for specific materials, systems and methods

We require information from authors about some types of materials, experimental systems and methods used in many studies. Here, indicate whether each material, system or method listed is relevant to your study. If you are not sure if a list item applies to your research, read the appropriate section before selecting a response.

Materials & experimental systems

n/a	Involved in the study	
[ ]	[x]	Antibodies
[ ]	[x]	Eukaryotic cell lines
[x]	[ ]	Palaeontology and archaeology
[ ]	[x]	Animals and other organisms
[ ]	[x]	Human research participants
[x]	[ ]	Clinical data
[x]	[ ]	Dual use research of concern

Methods

n/a	Involved in the study	
[x]	[ ]	ChIP-seq
[x]	[ ]	Flow cytometry
[x]	[ ]	MRI-based neuroimaging

Antibodies

Antibodies used

Antibodies	Supplier	Catalog No.	Clone No.	Dilution
p63	Abcam	ab735	4A4	1:100 (IF)
Human nucleoli	Abcam	ab190710	NM95	1:200 (IHC)
Muc5AC	Abcam	ab198294	EPR16904	1:250 (IF) 1:500 (IHC)
Involucrin	Sigma	HPA055211	n/a	1:500 (IHC)
Ki67	Abcam	ab15580	n/a	1:1000 (IF)
p75 NGFR	Abcam	ab52987	EP1039Y	1:50 (IF)
CK14	Proteintech	10143-1-AP	n/a	1:400 (IF)
Fibronectin	Proteintech	15613-1-AP	n/a	1:200 (IF)
CK10	Abcam	ab76318	EP1607IHCY	1:200 (IF)
CK5	Invitrogen	MA5-14473	EP1601Y	1:100 (IF)
Uteroglobin	Invitrogen	MA5-17170	3A8B8	1:200 (IF)
Foxj1	Novus Biologicals	NBP1-87928	n/a	1:200 (IF) 1:500 (IHC)
Acetylated Tubulin	Sigma	T7451	6-11B-1	1:30,000 (IF)
CK8	Novus Biologicals	NBP1-48281	OTI1B12	1:400 (IF)
BMP2	Abcam	ab214821	EPR20807	1:150 (IF)
E-Cadherin	Bio-Techne	AF648	n/a	1:200 (IF)

Phospho-Smad1/5	CST	#9516	41D10	1:500 (IF)
Phospho-Smad2	CST	#18338	E8F3R	1:500 (IF)
PTHLH	Affinity	DF6532	n/a	1:150 (IF)
Aggrecan	Abcam	ab3778	6-B-4	1:500 (IHC)
Osteocalcin	Abcam	ab13418	OC4-30	1:100 (IHC)
Donkey anti-Goat Alexa 488	Invitrogen	A32814		
Donkey anti-Rabbit Alexa 594	Invitrogen	A21207		
Goat anti-Rabbit Alexa 594	Abcam	ab150080		
Goat anti-Mouse Alexa 488	Abcam	ab150113		

Validation

Validation data / citations of all primary antibodies used in this study can be found on the manufacturer website by searching the antibody catalog number provided in methods section of our manuscript.

[Anti-p63 antibody [4A4] (ab735)] application: IHC-P; species reactivity: Human
https://www.abcam.com/p63-antibody-4a4-ab735.html

[Anti-Human Nucleoli antibody [NM95] (ab190710)] application: WB, IHC-P, Flow Cyt (Intra), ICC; species reactivity: Human
https://www.abcam.com/Human-Nucleoli-antibody-NM95-Nucleolar-Marker-ab190710.html

[Anti-Mucin 5AC antibody [EPR16904] (ab198294)] application: WB, IHC-P, ICC/IF; species reactivity: Human
https://www.abcam.com/mucin-5ac-antibody-epr16904-ab198294.html

[Anti-IVL antibody (HPA055211)] application: IHC, IF; species reactivity: Human
https://www.sigmaaldrich.cn/CN/zh/product/sigma/hpa055211

[Anti-Ki67 antibody (ab15580)] application: IHC-P, ICC; species reactivity: Mouse, Human
https://www.abcam.com/Ki67-antibody-ab15580.html

[Anti-p75 NGF Receptor antibody [EP1039Y] (ab52987)] application: WB, IP, IHC-P, ICC/IF, Flow Cyt (Intra); species reactivity: Mouse, Rat, Human
https://www.abcam.com/p75-NGF-Receptor-antibody-EP1039Y-ab52987.html

[Anti-Cytokeratin 14 antibody (10143-1-AP)] application: WB, IP, IHC, IF, FC (Intra); species reactivity: Human, Mouse, Rat
https://www.ptglab.com/products/KRT14-Antibody-10143-1-AP.htm

[Anti-Fibronectin antibody (15613-1-AP)] application: WB, IP, IF, FC; species reactivity: Human, Mouse, Rat
https://www.ptglab.com/Products/FN1-Antibody-15613-1-AP.htm

[Anti-Cytokeratin 10 antibody [EP1607IHCY] (ab76318)] application: ICC/IF, WB, IHC-P; species reactivity: Mouse, Rat, Human
https://www.abcam.com/cytokeratin-10-antibody-ep1607ihcy-cytoskeleton-marker-ab76318.html

[Anti-Cytokeratin 5 antibody [EP1601Y] (MA5-14473)] application: WB, IHC-P, ICC/IF; species reactivity: Human, Mouse
https://www.thermofisher.cn/cn/zh/antibody/product/Cytokeratin-5-Antibody-clone-EP1601Y-Monoclonal/MA5-14473

[Anti-Uteroglobin antibody [3A8B8] (MA5-17170)] application: WB, FC, ELISA; species reactivity: Human
https://www.thermofisher.cn/cn/zh/antibody/product/Uteroglobin-Antibody-clone-3A8B8-Monoclonal/MA5-17170?adobe_mc=MCMID%7C59062698389797485434394331790822104606%7CMCAID%3D2F53C7D58515EBD7-60000792AAD00F99%7CMCORGID%3D5B135A0C5370E6B40A490D44%40AdobeOrg%7CTS=1614293705

[Anti-FoxJ1/HFH4 antibody (NBP1-87928)] application: IHC, IHC-Fr, IHC-P; species reactivity: Human, Mouse
https://www.novusbio.com/products/foxj1-hfh4-antibody_nbp1-87928

[Anti-Acetylated Tubulin antibody [6-11B-1] (T7451)] application: WB, IHC, RIA, EM, DB; species reactivity: plant, hamster, rat, mouse, human, pig, monkey, frog, invertebrates, bovine, protista, chicken
https://www.sigmaaldrich.cn/CN/zh/product/sigma/t7451

[Anti-Cytokeratin 8 Antibody [OTI1B12] (NBP1-48281)] application: WB, ICC/IF, IHC, IHC-P; species reactivity: Human, Mouse, Rat, Canine, Monkey
https://www.novusbio.com/products/cytokeratin-8-antibody-oti1b12_nbp1-48281

[Anti-BMP2 antibody [EPR20807] (ab214821)] application: Flow Cyt (Intra), WB, ICC/IF; species reactivity: Mouse, Rat, Human
https://www.abcam.com/bmp2-antibody-epr20807-ab214821.html

[Anti-Human/Mouse E-Cadherin antibody (AF648)] application: CyTOF-ready, Dual RNAscope ISH-IHC, Flow Cyt, IHC, IF, WB; species reactivity: Human, Mouse
https://www.bio-techne.com/cn/p/antibodies/human-mouse-e-cadherin-antibody_af648

[Anti-Phospho-Smad1/5 (Ser463/465) [41D10] (#9516)] application: WB, IF, Flow Cyt; species reactivity: Human, Mouse, Rat

https://www.cellsignal.com/products/primary-antibodies/phospho-smad1-5-ser463-465-41d10-rabbit-mab/9516

[Anti-Phospho-SMAD2 (Ser465/Ser467) [E8F3R] (#18338)] application: WB, IP, IF, Flow Cyt, ChIP; species reactivity: Human, Mouse, Rat
https://www.cellsignal.com/products/primary-antibodies/phospho-smad2-ser465-ser467-e8f3r-rabbit-mab/18338

[Anti-PTHLH antibody (DF6532)] application: WB, IHC, IF/ICC, ELISA (peptide); species reactivity: Human, Mouse, Rat
http://www.affbiotech.com/goods-5334.html

[Anti-Aggrecan antibody [6-B-4] (ab3778)] application: WB, IHC-P; species reactivity: Human, Recombinant fragment
https://www.abcam.com/Aggrecan-antibody-6-B-4-ab3778.html

[Anti-Osteocalcin antibody [OC4-30] (ab13418)] application: WB, IHC-Fr, Sandwich ELISA, ICC/IF, IHC-P; species reactivity: Rat, Sheep, Rabbit, Goat, Cow, Dog, Human, Pig
https://www.abcam.com/Osteocalcin-antibody-OC4-30-ab13418.html

Eukaryotic cell lines

Policy information about cell lines

Cell line source(s)

Human bone marrow-derived multipotent stromal cells (MSC) were purchased from Fuyuan Biotechnology (Shanghai, China).

Authentication

Cells were authenticated to be CD45+/CD73+/CD90+/CD105+/CD166+/CD11b-/CD34-/HLA-DR- using flow cytometry by provider right before shipment, and verified by differentiation assay upon receiving.

Mycoplasma contamination

Cells were tested to be negative for mycoplasma contamination using TaKaRa PCR Mycoplasma Detection Set.

Commonly misidentified lines
(See ICLAC register)

No commonly misidentified cell line was used in this study.

Animals and other organisms

Policy information about studies involving animals; ARRIVE guidelines recommended for reporting animal research

Laboratory animals

Species	Strain	Cat. No.	Sex	Age
Mouse	NOD-Prkdcscidll2rgem1/Smoc	NM-NSG-001	Both	6-8 weeks (at the starting point of experiment)

from Shanghai Model Organisms, China, https://www.modelorg.com/en/portal/article/index/id/841/post_type/3.html.

Animals were housed in SPF facility with room temperature 20-26°C, relative humidity 30-70%, with 12-hour dark/light cycles.

Wild animals

This study did not involve wild animals.

Field-collected samples

This study did not involve samples collected from the field.

Ethics oversight

All experiments were conducted in accordance with the regulations of IACUC committee of Shanghai Sixth People's Hospital.

Note that full information on the approval of the study protocol must also be provided in the manuscript.

Human research participants

Policy information about studies involving human research participants

Population characteristics

This study recruited seven non-TO control donors and six TO patients that had been clinically and histopathologically diagnosed from the study period since 2018. Details of the covariate-related population characteristics of human research participants (such as age, gender, smoking history) were provided in Table 1.

Recruitment

We recruited all diagnosed patients with complete basic information and evidenced by symptomatic, CT, bronchoscopy and histological indications.

Ethics oversight

The study protocol was approved by the Ethics Committee of Shanghai Sixth People's Hospital, and informed consent was obtained from all individuals recruited for this study. The study design and conduct complied with all relevant regulations regarding the use of human study participants and was conducted in accordance with the criteria set by the Declaration of Helsinki. For recruited individuals, cytological examination, bronchoscopy and follow-up consultation were provided for free as a compensation.

Note that full information on the approval of the study protocol must also be provided in the manuscript.
